# Design of multi-epitope vaccine candidate based on OmpA, CarO and ZnuD proteins against multi-drug resistant *Acinetobacter baumannii*

**DOI:** 10.1016/j.heliyon.2024.e34690

**Published:** 2024-07-16

**Authors:** Batul Negahdari, Parisa Sarkoohi, Forozan Ghasemi nezhad, Behzad Shahbazi, Khadijeh Ahmadi

**Affiliations:** a Student Research Committee, Faculty of Pharmacy, Hormozgan University of Medical Sciences, Bandar Abbas, Iran; b Department of Pharmacology and Toxicology, Faculty of Pharmacy, Hormozgan University of Medical Sciences, Bandar Abbas, Iran; c School of Pharmacy, Semnan University of Medical Sciences, Semnan, Iran; d Nervous System Stem Cells Research Center, Semnan University of Medical Sciences, Semnan, Iran; e Infectious and Tropical Diseases Research Center, Hormozgan Health Institute, Hormozgan University of Medical Sciences, Bandar Abbas, Iran

**Keywords:** *Acinetobacter baumannii*, Epitope, Vaccine, Prediction, Molecular dynamics

## Abstract

*Acinetobacter baumannii* has been identified as a major cause of nosocomial infections. Acinetobacter infections are often difficult to treat with multidrug resistant phenotypes. One of the most effective ways to combat infectious diseases is through vaccination. In this study, an attempt was made to select the most protective and potent immunostimulatory epitopes based on the epitope-rich domains of the ZnuD, OmpA and CarO proteins of *Acinetobacter baumannii* to design a vaccine that can protect against this infection. After predicting the epitope of B- and T-cells, seven antigenic regions of three proteins CarO, ZnuD and OmpA, were selected. These regions were bound by a GGGS linker. The binding affinity and molecular interactions of the vaccine with the immune receptors TLR2 and TLR4 were studied using molecular docking analysis. This vaccine design was subjected to in silico immune simulations using C-ImmSim. The designed vaccine was highly antigenic, non-allergenic and stable. TLR2 and TLR4 were selected to analyze the ability of the modeled chimeric protein to interact with immune system receptors. The results showed strong interaction between the designed protein vaccine with TLR2 (−18.8 kcal mol-1) and TLR4 (−15.1 kcal mol-1). To verify the stability of the interactions and the structure of the designed protein, molecular dynamics (MD) simulations were performed for 200 ns. Various analyses using MD showed that the protein structure is stable alone and in interaction with TLR2 and TLR4. The ability of the vaccine candidate protein to stimulate the immune system to produce the necessary cytokines and antibodies against *Acinetobacter baumannii* was also demonstrated by the ability of the protein designed using the C-ImmSim web server to induce an immune response. Therefore, the designed protein vaccine may be a suitable candidate for in vivo as well as in vitro studies against *Acinetobacter baumannii* infections.

## 1 Introduction

*Acinetobacter baumannii (A. baumannii)* is a Gram-negative, obligate aerobic, lactose-negative bacterium that is increasingly recognized as an important cause of hospital-acquired infections such as bacteremia and ventilator-associated pneumonia, surgical site infections, secondary meningitis, and urinary tract infections, particularly in patients in the intensive care unit (ICU) patients. Treatment of Acinetobacter infections is often complicated by multi-drug resistant phenotypes such as resistance to broad-spectrum beta-lactams, aminoglycosides and fluoroquinolones [1,2].

Multidrug-resistant *A. baumannii* is the leading cause of ventilator-associated infections among all ESKAPE pathogens, which include *Enterococcus faecium, Staphylococcus aureus, Klebsiella pneumoniae, Acinetobacter baumannii, Pseudomonas aeruginosa, and Enterobacteremia species* [3]. Today, many efforts are being made to develop vaccines and drugs against infectious diseases [[4], [5], [6], [7], [8]]. In addition, *A. baumannii* secondary or nosocomial infections have been reported worldwide during the 2019 coronavirus outbreak [9]. A number of resistance mechanisms are known in *A. baumannii* against many classes of antibiotics. These include β-lactamases, multidrug efflux pumps, aminoglycoside-modifying enzymes, permeability defects and altered target sites. Different classes of antibiotics can be targeted by most of these resistance mechanisms [10]. In the 21st century, the emergence and rapid spread of resistant bacteria around the world has overshadowed the power of antibiotics, and vaccination is the safest and most effective alternative method against pathogenic infections [11]. Vaccination, especially in high-risk groups, may be an effective prevention method for this pathogen [12]. Vaccination is generally considered to be one of the most effective methods of preventing infectious diseases. All vaccines work by presenting a foreign antigen to the immune system in order to induce an immune response. The active agent of the vaccine may be the whole inactivated or weakened pathogen, or purified parts of the pathogen that are highly immunogenic [13]. Traditional vaccinology requires a large amount of specialized biomolecular equipment, trained laboratory personnel, and appropriate animal models, followed by long intervals between experiments. Advances in biological databases and computational methods have been driven by advances in biomedical informatics coupled with new genome sequencing technologies. This continuous flow of information will increase our knowledge of host-pathogen interactions. It will also aid in vaccine research against infectious and non-infectious diseases. Recent advances in bioinformatics, immunoinformatics, and vaccinology have ushered in a new era of vaccine science with a more contemporary, powerful, and realistic approach to the design and development of the next generation of potent immunogens. Computational tools and techniques that are highly effective, economical, accurate, robust, and safe for use in humans are directly relevant to vaccine research [14]. Since both types of B cells and T cells have a role in the fight against *A. baumannii* infection, the prediction of B cell and T cell epitopes may be important in the design of a vaccine against *A. baumannii* in order to stimulate both arms of the immune system [15].

Several virulence factors are expressed by *A. baumannii* that lead to different diseases. These factors include purine proteins, efflux pumps, outer membrane vesicles, metal acquisition systems, secretion systems, phospholipases, and capsular polysaccharides [10]. OMPs are important immunogenic proteins in bacteria that confer properties such as immune evasion, stress tolerance, and antibiotic/antimicrobial resistance [16]. While several factors may be involved in the pathogenic potential of *A. baumannii*, one particular factor, OmpA, is significantly involved in the pathogenic potential of the pathogen, as well as complement resistance and biofilm formation [17]. In a study, significant reductions in *A. baumannii* bacterial loads were observed in the spleen, liver, and lungs of the immunized mouse groups following exposure to selected loops of BauA and OmpA as vaccine candidates [18]. The results also showed that OmpA can be used for the production of monoclonal antibodies against *A. baumannii* [19]. Carbapenem resistance in *A. baumannii* is one of our major challenges. The loss of an outer membrane protein called CarO (Carbapenem Resistant Outer Membrane Protein) is associated with carbapenem resistance in *A. baumannii* [20]. It is mainly the alteration or loss of the purine region in the outer membrane that leads to the development of carbapenem-resistant *A. baumannii*. This protein has been the target of drug discovery efforts [21]. CarO was first reported in *A. baumannii* isolates susceptible to imipenem, which acquired resistance after the loss of the 29 kDa protein [16]. The use of specific antibodies against this protein under laboratory conditions has a bacteriostatic or bactericidal effect [22]. As a result, this protein can be introduced as a target antigen for vaccines.

In the interaction between pathogen and host, metals such as zinc are essential elements. They play an important role in the physiology and pathogenicity of *A. baumannii,* as cofactors of many DNA-binding enzymes and proteins [23]. Pathogenic bacteria, including *A. baumannii*, require zinc metal as a nutrient for survival, which they must obtain from their host [24]. During the last decade, due to the increasing importance of *A. baumannii* in nosocomial infections, especially in intensive care and burn units, many studies have been conducted on this bacterium in different parts of the world. These studies have shown that there is a high prevalence of antibiotic resistance in *A. baumannii* [[25], [26], [27]]. In this study, we attempted to design a multi-epitope protein targeting different mechanisms by using these three proteins that play important roles in the survival and function of *A. baumannii*.

In the present study, we used reverse vaccinology to find epitope-rich domains of the ZnuD, OmpA and CarO proteins. These proteins are essential for the function and survival of *A. baumannii*. After designing the vaccine candidate, we used computational methods to analyze the interaction of the designed chimeric protein with TLR2 and TLR4. This evaluation measures the potential biological activity of the proposed protein. Finally, the effect of the candidate protein vaccine on the induction of immune system responses, including cytokines and immunoglobulins, was analyzed using in silico immune response simulations.

## 2 Materials and methods

### 2.1 Retrieval of the ZnuD, OmpA, and CarO protein sequences

The sequences of *A. baumannii* OmpA, CarO and ZnuD proteins were extracted from the UniProtKB (https://www.uniprot.org/). An easy-to-use interface to locate the desired protein and learn about protein information is available on the UniProt website [28].

### 2.2 T cell epitope prediction

Understanding how the body's immune system responds to different stimuli is critical to developing effective vaccines against bacterial infections. A sequence-based screening server called RANKPEP 1D has been used to identify T cell epitopes. Using a position-specific scoring matrix (PSSM), this site predicts immunodominant peptide interactions with MHC molecules [29]. Using the IEDB server (http://tools.immuneepitope.org/mhcii/), we used bioinformatics tools to predict HTL epitopes. Our selection criteria for vaccine design included epitopes with an IC50 value of less than 50 and the lowest percentile ranking as T cell epitopes. Eight specific HLA types (DRB1*0401, DRB1*0301, DRB1*1301, DRB1*0801, DRB1*1101, DRB1*0701, DRB1*0101, and DRB1*1501) were considered for predicting HLA class II epitopes on a global scale. These HLA types are known to encompass the genetic diversity of over 95 % of the human population worldwide [30].

### 2.3 Prediction of linear Bcell epitopes

B-cell epitopes, which are involved in the induction of the humoral immune response and the formation of antibodies that neutralize antigens during infection, are important in vaccine design [4]. Three popular databases, BepiPred, Kolaskar & Tongaonkar Antigenicity, and BCPred, were used to predict linear B cell epitopes. Finally, the epitopes with the highest scores were selected from the protected regions of the protein. Along with additional information on the host organism, immunological exposures, and inducible immune responses, the IEDB database collects studies that identify and define epitopes and epitope-specific immune receptors [31].

### 2.4 Selection of epitope-rich regions and vaccine candidates

To identify epitope-rich regions, we scored projected B-cell and T-cell epitopes. We selected portions of proteins that had high scores for both B-cell and T-cell epitopes, and these regions were then used to generate proteins. A combination of many epitopes was created. Finally, the regions of the OmpA, CarO, and ZnuD proteins with high concentrations of B-cell and T-cell epitopes were selected. These regions were then attached to various linkers, resulting in the vaccine sequence.

### 2.5 Physical and chemical characteristics of the final designed structure

The Protparam tools from the server (http://expasy.org/tools/protparam.html) were used to investigate the physical and chemical properties of the final designed structure. This included analysis of the number of amino acids, aliphatic index, molecular weight, instability index, isoelectric point (PI), estimated half-life, amino acid composition, number of charged amino acids, and grand average hydropathicity [32].

Physical and chemical properties are a reflection of a protein's functional and structural characteristics. The comparative study of physicochemical properties is important to know the role of a protein. It also helps to discover its molecular evolution [33].

### 2.6 Investigation of allergenicity, toxicity, solubility and antigenicity of the designed protein

The VaxiJen v2.0 server was used to evaluate the antigen of the proposed vaccine candidate. VaxiJen categorizes antigens using automated cross-covariance (ACC), a method that converts protein sequences into standardized vectors that represent important amino acid features [34]. Allergens are tiny antigens that typically trigger an immune response, including IgE antibodies. There are two categories of allergen prediction in bioinformatics. The first employs sequence similarity as a search criterion and complies with the FAO/WHO Codex Alimentarius guidelines. The second technique relies on finding linear patterns connected to preserved sensitivity. AllerTOP is a unique service that uses important physical and chemical properties of proteins to accurately predict allergens using in silico methods. The allergenicity of the proposed protein was evaluated using AllerTOP (http://www.ddg-pharmfac.net/AllerTOP/) [35]. In addition, the protein-sol server (https://protein-sol.manchester.ac.uk/) was used to estimate the trend of protein solubility. This website uses publicly available information on the solubility of *Escherichia coli* proteins in a cell-free expression system to calculate 35 sequence-based characteristics [36]. Also, the toxicity of the vaccine candidate was predicted by the Toxinpred (https://webs.iiitd.edu.in/raghava/toxinpred/) server [37].

### 2.7 Secondary and tertiary structure prediction

Using the Garnier-Osguthorpe-Robson (GOR) server, the secondary structure of the candidate protein was predicted from the amino acid sequence. Probability parameters empirically derived from known protein tertiary structures determined by X-ray crystallography are the basis of GOR [38]. The tertiary structure was modeled using the I-TASSER (https://zhanggroup.org/I-TASSER/) web server [39].

### 2.8 Evaluation of the tertiary structure of designed protein

The MolProbity (http://molprobity.biochem.duke.edu) [40], ProSA-web (https://prosa.services.came.sbg.ac.at/prosa.php) [41] and SAVES (https://saves.mbi.ucla.edu) [42] servers were used to evaluate predicted 3D structures. One of the most important problems is to identify errors in theoretical and experimental models of potential protein structures. The planned structures should be similar to the structure of naturally occurring proteins so that they can be produced in cells. The third structure was tested with ProSA-web to search for errors in the generated 3D models. The structure is evaluated using the energy plot and the Z-score. Z-scores outside the range of natural proteins indicate a 3D structure problem [41]. MolProbity is a widely used tertiary structure evaluation server for proteins, nucleic acids, and complexes. It is commonly used to verify the quality of three-dimensional structures of macromolecules. The web server analyzes several critical factors, including MolProbity score, Collision score, and Ramachandran diagram [43]. Ramachandran maps are important theoretical tools for predicting the allowed conformational space of amino acids in proteins. This map includes phi and psi angles. For this study, we used the Ramachandran plot from PROCHECK on the SAVES web server to determine the torsion angles of the amino acids in the chimeric protein. This analysis helped us to assess whether the residues in the remote regions were permissible and advantageous [42]. The ERRAT program finds errors based on the statistics of unbonded atom-atom interactions in the reported high-resolution structures [44]. The SAVES web server was used to calculate the ERRAT (https://saves.mbi.ucla.edu) and Verify 3D (https://saves.mbi.ucla.edu) scores by comparing them to statistics from highly refined structures.

### 2.9 Conformational B cell epitope prediction

The tertiary structure of the protein is necessary for discontinuous B-cell epitope prediction due to the critical nature of interactions between antibodies and antigenic epitopes. Using the ElliPro server, we were able to predict the structural epitopes of the potential vaccine protein. The Web server uses a customized iteration of Thornton's technique, MODELLER software, and Jmol to predict antibody epitopes. The ElliPro server uses a predetermined protein tertiary structure to predict B cell structural epitopes [45].

### 2.10 Investigating the interaction between recombinant protein with immune system receptors

The crystal structures of the TLR2 and TLR4 were obtained from the Protein Data Bank (PDB) database (https://www.rcsb.org) using the PDB IDs 53di and 7mlm, respectively. Water molecules and ligands were removed from both structures. Then, the Dock prep tool of the UCSF Chimera program (Chimera 1.5.3) was used to prepare ligand and receptor structures by adding hydrogen atoms and charges [46].

TLR2 and TLR4 are critical immune system receptors in the fight against *A. baumannii*. Important amino acids Tyr326, Leu317, Phe325, Ile319, Phe349, Val348, Phe322, Leu324, and Pro352 are located in the active site of the TLR2 [47]. Lys263, Gln339, Lys341, Arg380, Ser386, Ser413, Arg434, and are also essential residues in the TLR4 binding site [48].

Interactions of the designed protein with TLR2 and TLR4 of the immune system were evaluated using the Cluspro2 server (https://cluspro.bu.edu/home.php) [49]. For docking of proposed protein with TLR-2 and TLR-4, the HADDOCK 2.2 web server was also utilized. The HADDOCK service utilizes biochemical and/or biophysical data on interactions, such as chemical shift perturbation data obtained from NMR titration studies or mutagenesis data [50]. Residues involved in interacting between the chimeric protein and receptors were visualized using LigPlot + v. 4.5.3 [51]. The PRODIGY web service has also been used to estimate the affinity and binding energy of biological complexes. Finding biological interfaces and predicting binding affinities in complexes are the main areas of study for this site [52]. In addition, HawkDock was used to predict and analyze protein-protein complexes based on the computational MM/GBSA approach [53].

### 2.11 Molecular dynamics simulation

Based on the highest amount of hydrogen and hydrophobic bonds and the lowest free energy, the strongest vaccine-receptor complexes were selected for the next analysis. Molecular dynamics simulations were used to evaluate the structure of the produced protein and the stability of the complexes. The OPLS-AA force field and GROMACS 2018 software package [54] were utilized for this purpose. One nanometer separated the structure from each edge of a triaxial box. We used Gromax software to obtain the placement parameters of the protein structure. GROMACS software was used to analyze the positional properties of the protein structure. The unbound protein structure and complexes were then placed in a simulation box. The box was filled with TIP3P type water molecules. The total charge can be changed by the ligand-receptor interaction, and the total charge of the system can be changed from zero by the addition of water molecules. Chlorine ions neutralize a positive charge of the system and sodium ions neutralize a negative charge of the system throughout the simulation process. It was divided into two parts to minimize the energy of the complexes in the simulation. First, the systems underwent NVT (constant number of particles, volume, and temperature) equilibration at 300 K and 100 ps. The systems were then tuned to achieve the desired pressure and temperature. A Parrinello-Rahman barostat was used to calibrate NPT (constant particle number, pressure, and temperature) at 300 K, 1.0 bar, and 100 ps. The Ewald mesh particle approach was used to handle long-range electrostatic charges, with a no-effect distance of 10 Å [55]. van der Waals interactions were calculated based on a 1 nm distance. The length of the covalent bonds was constrained using a linear constraint approach. After applying the required adjustments, a 200 ns simulation was performed on the selected complexes using the molecular docking technique and the designed protein. To determine the stability and changes in the protein structure during the simulation, the output trajectories were subjected to several analyses, including principal component analysis (PCA), radius of gyration (RG), root mean square fluctuations (RMSF), and free energy landscapes (FELs).

### 2.12 Simulation of the immune system

To evaluate the immunogenicity and immunological response profile of the vaccine, an immune simulation study was conducted. The C-ImmSim simulation server (http://150.146.2.1/C-IMMSIM/index.php) was used to induce an immune response against the proposed protein. The simulation was run with default settings with time steps of 1, 84, and 168. Three doses of immunization were given automatically. The simulation was run with a 10 μL simulation volume, 1000 simulation steps, and a vaccination chosen without LPS and with an adjuvant [56].

### 2.13 Cloning and optimization

To improve the production of recombinant proteins, the instrument (JCat) was used to optimize codons for nucleotide sequences in *E. coli* (K12 strain). The percentage of GC content and CAI were measured. CAI is the primary index often used to make predictions about gene expression levels. It is a measure of the extent to which the coding sequence accurately represents the use of codons in an organism [57]. The optimized protein nucleotide sequences were cloned into the pET28a vector using the SnapGene program to assess double digestion.

## 3 Results

### 3.1 Genome extraction of carO, ZnuD and OmpA proteins

The Uniprotkb database was used to extract the protein sequences for ZnuD (accession number: WP_001984799.1), Omp38 (accession number: Q6RYW5), and CarO (accession number: A0A1L7FTI1).

### 3.2 Prediction of B-Cell epitopes

As shown in Supplementary Table S1, BCPred, Bepipred, and IEDB servers (Kolaskar and Tongaonkar) were used to predict B-cell epitopes for all three proteins: carO, ZnuD, and OmpA.

### 3.3 Prediction of T-Cell epitopes

T cell epitope predictions were also performed using the Rankpep and IEDB databases. The MHCII alleles DRB1*0101, *1301, *1101, *0401, *0701, *0301, *0801, and *1501 were selected to form an allelic group that would encompass the majority of human genetic backgrounds. Supplementary Table S2 shows the epitopes that were considered most significant based on their scores.

### 3.4 Selection of epitope-rich regions

To select the domains that will construct the vaccine candidate, the regions of these three proteins with the highest epitope abundance are considered the target domain for vaccine design. Under these conditions, seven epitope-rich domains from these three proteins were finally selected as vaccine candidates, containing many B-cell and T-cell epitopes (Table 1).

**Table 1:** Seven epitope rich domains selected from three proteins CarO, ZnuD and OmpA.

Antigen	position	Antigenic determinant
**CarO**	22–85 117–160	DEAVVHDSYAFDKNQLIPVGVRAEVGTTGYGGALLWQANPYVGLALGYNGGDISWRDDVSVNGT YIAAGAAYLDNDYDLAKRIGNGGTLTIDGNTYQQAAAGQEGGVR
**OmpA**	113–192 230–350	KNYDSKIKPYVLLGAGHYKYDFDGVNRGTRGTSEEGTLGNAGVGAFWRLNDALSLRTEARATYNADEEFWNYTALAGLNV LRVFFDTNKSNIKDQYKPEIAKVAEKLSEYPNATARIEGHTDNTGPRKLNERLSLARANSVKSALVNEYNVDASRLSTQGFAWDQPIADNKTKEGRAMNRRVFATITGSRTVVVQPGQEAA
**ZnuD**	420–500 121–255 530–600	IITEPAWWGGNPDLKPEESVSYELGFDQKLNHGFNVYGSVYQTKVDNLMVSSAATNFVFYNIDKATLTGAELGLKWSLDNW TATVAGQNIHLFDTTDIKQIEILRGPASVQYGTDAIGGVIQLISKTPTQNKIFTTIEAGEKNTYKSILGIDLAQDGYYAQIRGQRFETDGDQIISNDDRKAGFDQKGYSAKVGVDKEQYALSAEIKENKGTGDFF MNIGWDNGVYGFNTAFVAKGKAKDIQDVPGYTTLDFNAYWQMSPN

### 3.5 Protein design using selected domains and different linkers

**Fig. 1:**
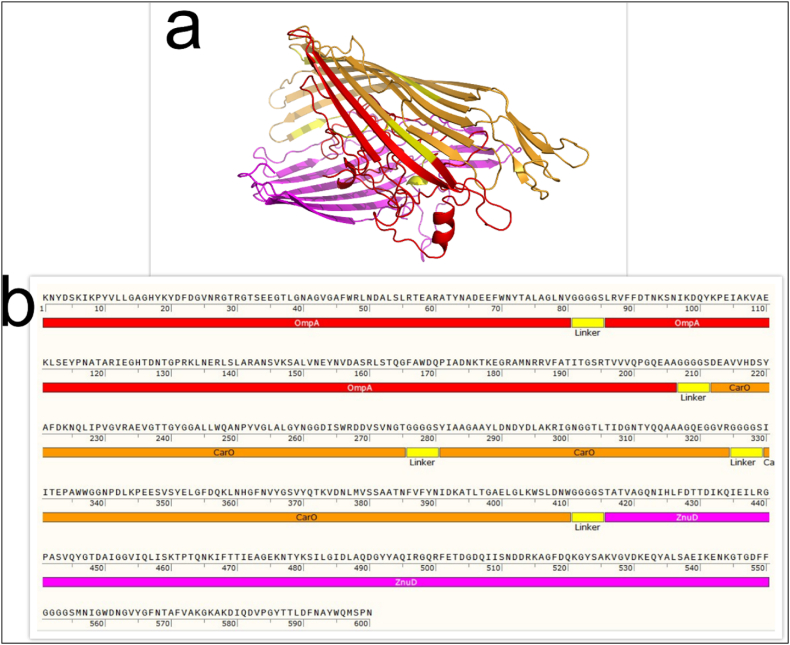
(a) The tertiary structure of the designed protein using the I-TASSER web server. (b) Schematic representation of the final multi-epitope protein construct generated using SnapGene.

A total of 12 protein sequences were generated by integrating specific domains with different linkers such as EAAAK and GGGGS. The 12 generated sequences were analyzed based on their physicochemical properties, secondary structure, antigenicity, allergenicity, solubility and tertiary structure. As shown in Fig. 1a–b, the most suitable protein was finally presented as a potential vaccine.

### 3.6 Physical and chemical properties of designed structures

The Protscale and Protparam tools (http://expasy.org/tools/protparam.html) were used to determine the chemical and physical properties of the developed structures, such as hydrophobicity, hydrophilicity, number of charged amino acids, molecular weight, and number of amino acids. The physicochemical properties of the vaccine candidate were evaluated using a ProtParam evaluation tool. This analysis showed that the final vaccine candidate protein we produced had a molecular weight of 64296.85 Da and 600 amino acids. The theoretical isoelectric point (PI) was found to be 5.10. An instability score of less than 40 indicates that the protein used to elicit an immune response is extremely stable. The instability score of our chimeric protein was 26.41. This indicates that it is stable. The aliphatic index of the designed chimeric protein was 71.75, indicating that it is stable at different temperatures.

### 3.7 Evaluation of antigenicity, allergenicity, toxicity and solubility

The antigenicity of the proposed protein was calculated using the VaxiJen v2.0 server. The results indicated that the vaccine candidate had a strong potential to boost the immune system. The predicted antigenicity of the selected protein was 0.9047. The allergenicity of the proposed structure was checked using the Evaller web server. The developed protein was not allergenic. In addition, the solubility of the chimeric protein was determined by employing the Protein-Sol server, which showed that the protein was well soluble. A protein we have chosen is 0.477 solubility. The developed protein was submitted to the ToxinPred server and the results indicated that the vaccine candidate was not toxin as shown in Supplementary Table S3.

### 3.8 Secondary and tertiary structure prediction and validation

**Fig. 2:**
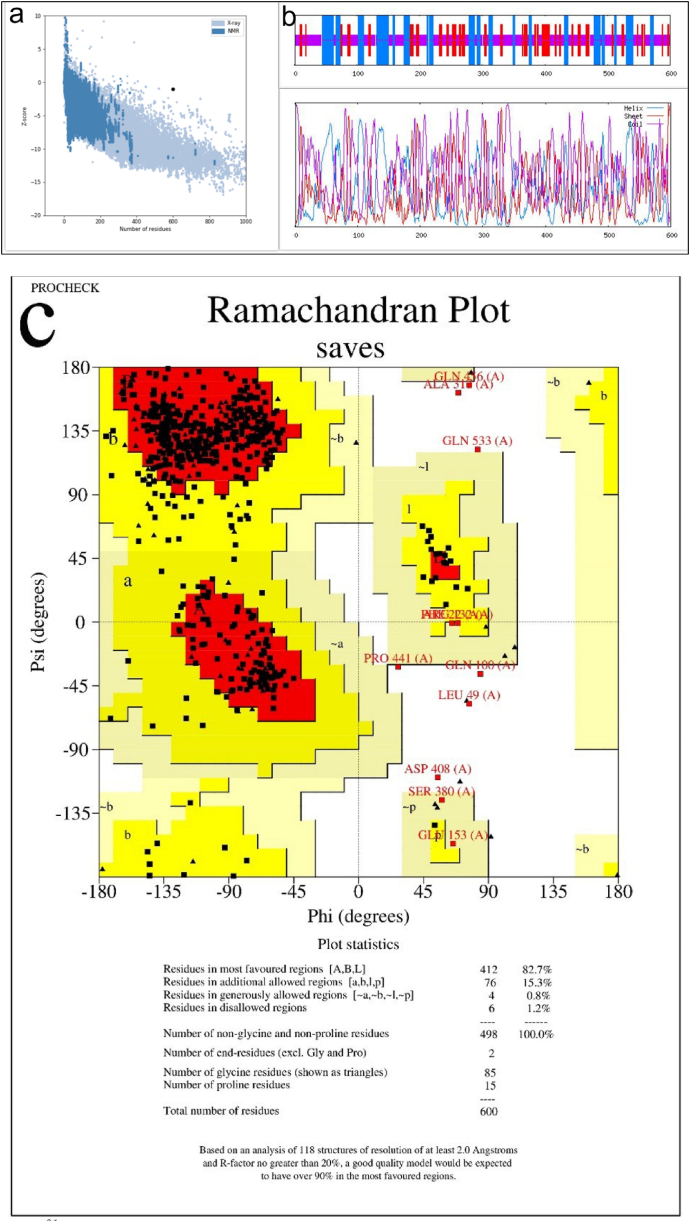

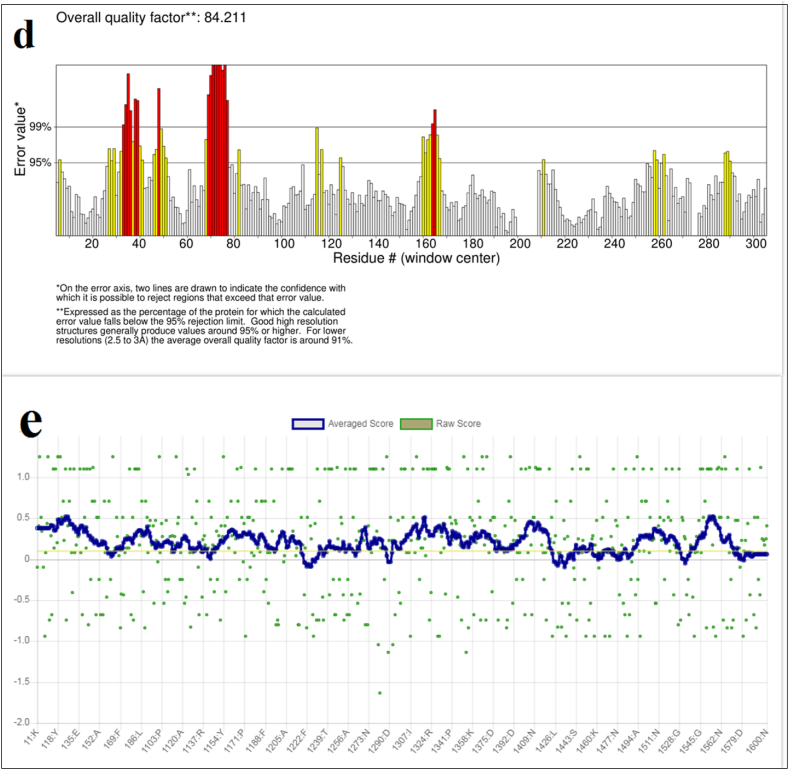
Evaluation and validation of the predicted secondary and tertiary structure of the designed chimeric protein. (a) Secondary structure of the designed protein. Alpha-helix (blue), beta-strand (red), and random coils (purple). (b) Evaluation of the tertiary structure of a protein modeled with ProSA-web. NMR (blue) and X-ray spectroscopy (light blue). (c) Validation of the tertiary structure of the protein by Ramachandran plot. Ramachandran plots show the residues that are in the most favored (red), additionally allowed (yellow), generously allowed (light yellow), and not allowed (white) regions. (d) The ERRAT score of the vaccine candidate indicates the acceptable quality of the structure. (e) Validation of the tertiary structure of the protein by Verify 3D.

The second structure of the projected protein was evaluated using the GOR software. Fig. 2a shows that of the 600 amino acids, 158 (33/26 %) are alpha helices, 128 (33/21 %) are extended strands, and 314 (33/52 %) are random coils. The I-TASSER service predicted tertiary structures for the generated protein sequences. After validation of all structures, the optimal structure was selected. The predicted tertiary structures were evaluated using MolProbity, ProSA-web and SAVES servers. Structural similarities of new proteins to the best known structures of related proteins were evaluated using the MolProbity service (http://molprobity.biochem.duke.edu/index.php). The MolProbity analysis and the Clash score were used to evaluate the protein structure analysis. The SAVES server was also used to confirm the Ramachandran plot and to evaluate the distribution of amino acids in the favored, allowed and disallowed sectors (https://saves.mbi.ucla.edu/).

According to the MolProbity analysis, this protein has a Clash score of 8.77 (78 % best among all structures) for its structure. The MolProbity score was 2.02, placing it in the 78 % of all structures. ProSA-web used an energy plot and Z-score to examine a three-dimensional model of the vaccine candidate. The Z-score of the selected protein was −0.96, which was within the range of 10 to −20 of the original protein structure, as shown in Fig. 2b [41]. In the Ramachandran plot analysis of the desired protein, 98.8 % of the amino acids were found in the desired and allowed regions and 1.2 % in the unallowed regions, indicating that the predicted structure of the protein was plausible. A low G-factor is strongly correlated with a low probability of structural occurrence. Increasing the number of residues in the restricted regions decreases the G-factor value. Values less than −0.5 are considered rare, while values less than −1.0 are considered extremely rare. Structures with a value greater than −0.5 are considered acceptable models. The structure we created, which has a G-factor of −0.18, is considered acceptable. This can be seen in Fig. 2c and Table 2. The ERRAT analysis identifies flawed regions in protein structures based on the presence of atoms that deviate from the expected distribution, allowing them to be distinguished from the correct distributions. The ERRAT value of the projected structure in this study was 84.211 %, indicating that the quality of the structure is satisfactory (Fig. 2d). Verify 3D reports that the protein design contains 80.00 % of residues with an average 3D-1D score of at least 0.1 (Fig. 2e), indicating that the predicted tertiary structure is valid.

**Table 2:** Ramachandran plot statistics of a designed vaccine candidate calculated with PROCHECK.

Regions	Residues	Percentage
Most favored regions	412	82.7 %
Additional regions allowed	76	15.3 %
Generously allowed regions	4	0.8 %
Disallowed regions	6	1.2 %
Non-glycine and non-proline residues	498	100 %
End-residues (excl. Gly and Pro)	2	
Glycine residues	85	
Proline residues	15	
Total number of residues	600	
**G-Factors**		**Average Score**
Dihedrals		−0.03
Covalent		−0.44
Overall		−0.18

### 3.9 Prediction of conformational B-cell epitopes

**Fig. 3:**
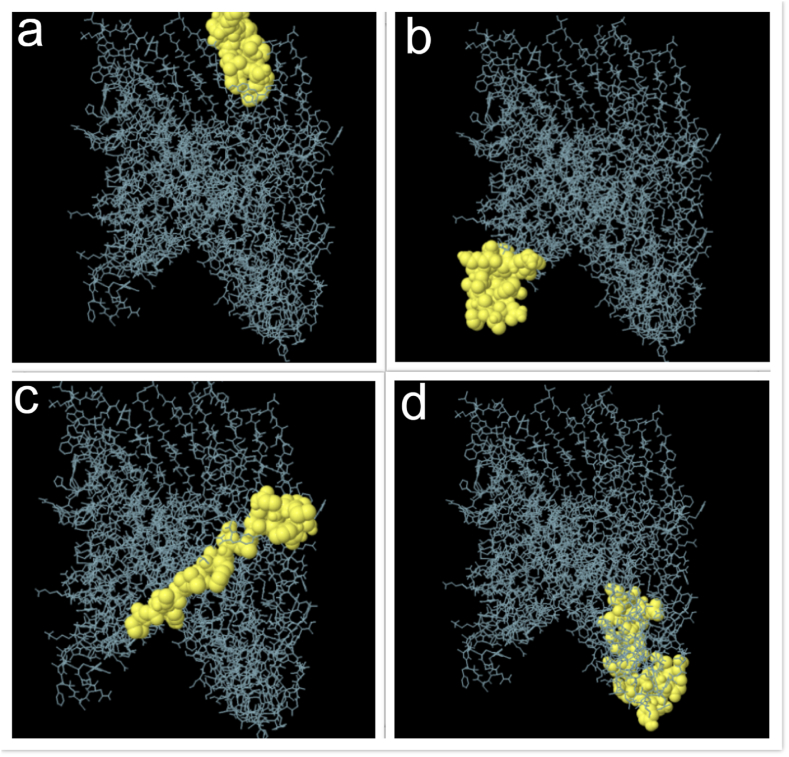
The most potent vaccine candidate conformational epitopes designed using the Ellipro server. a) 13 residues (AA 218–230) with residue score 0.788; b) 22 residues (AA 558–579) with residue score 0.782; c) 17 residues (AA 584–600) with residue score 0.765; d) 27 residues (AA 295–321) with residue score 0.763.

Analysis of the 3D structure of the vaccine candidate using ElliPro Server revealed conformational epitope regions (Fig. 3 a-d). Predicted conformational B cell epitopes ranged from 0.763 to 0.788 (Table 3).

**Table 3:** Prediction of B-Cell conformational epitopes by the Ellipro web server.

No.	Start	End	Peptide	Number of residues	Score
1	218	230	DSYAFDKNQLIPV	13	0.788
2	558	579	IGWDNGVYGFNTAFVAKGKAKD	22	0.782
3	584	600	PGYTTLDFNAYWQMSPN	17	0.765
4	295	321	LAKRIGNGGTLTIDGNTYQQAAAGQEG	27	0.763

### 3.10 Protein–protein molecular docking

**Fig. 4:**
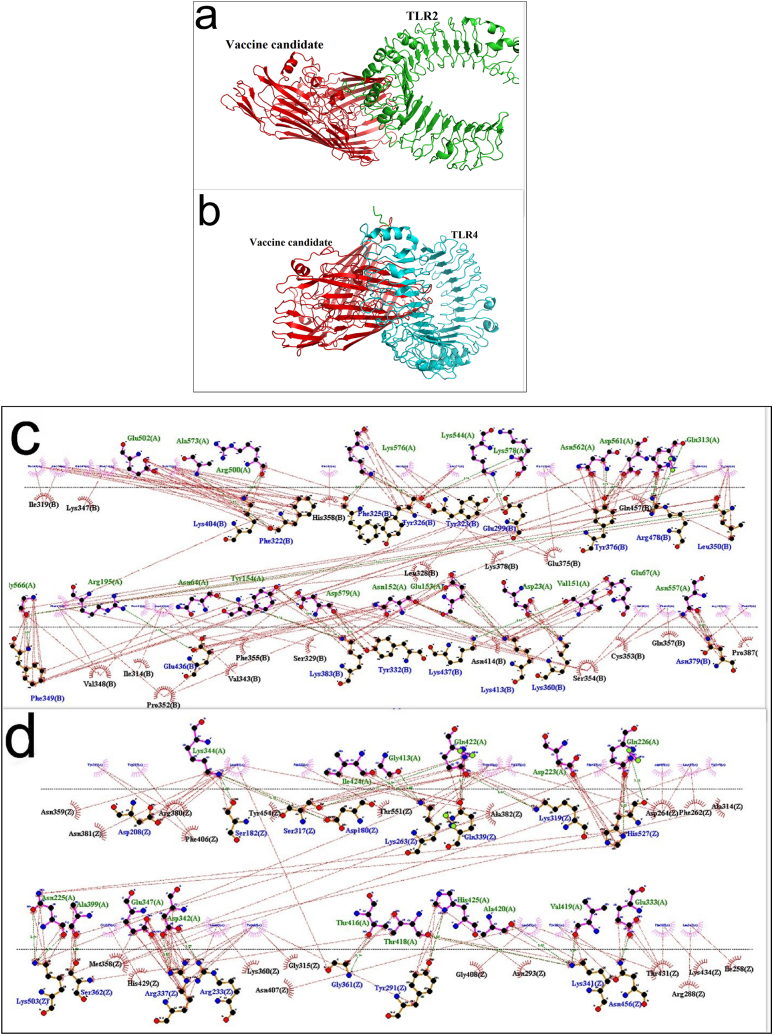
(a) Cartoon illustration of the interaction of the chimeric protein with TLR2. (b) Cartoon illustration of the interaction of the chimeric protein with TLR4. (C) Results of the analysis of the residues involved in the interaction between the chimeric protein and TLR2 using LIGPLOT. (D) Results of the analysis of the residues involved in the interaction between the chimeric protein and TLR4 using LIGPLOT. * The dark green line represents the hydrophobic interaction with the receptor (black) and the hydrophobic interaction with the receptor (blue).

Molecular docking was performed using Cluspro 2.0 and HADDOCK servers to evaluate the ligand-receptor binding between chimeric protein with TLR2 (Fig. 4a) and TLR4 (Fig. 4b). Appropriate interactions were selected by parameters such as number of clusters, weighted score and HADDOCK score. The LIGPLOT program was also used to visualize residues involved in hydrogen and hydrophobic bonds between the chimeric protein with TLR2 (Fig. 4c) and TLR4 (Fig. 4d). Finally, we considered the lowest energy, the most negative weighted score, the lowest affinity (Kd), and the highest number of amino acids involved in hydrogen bonding and hydrophobicity were considered as essential standards for selecting the strongest complexes. Results revealed strong interactions between vaccine candidate with TLR4 and TLR2 (Table 4). PyMOL and LIGPLOT programs were used to observe the interactions between TLR4 and TLR2 with the designed vaccine candidate (Fig. 4). We considered the lowest energy, the most negative weight result, the lowest Kd for affinity, and the highest number of amino acids involved in hydrogen bonding and hydrophobicity as essential standards for selecting the strongest complexes. Molecular docking results using Cluspro 2.0 and HADDOCK servers showed a strong interaction between the chimeric protein with both TLR4 and TLR2 (Table 4). The designed vaccine candidate formed a strong interaction with the key amino acids of the active site of the immune system receptors. This binding includes the essential amino acids Lys263, Lys341, Arg383, and Gln339 for TLR4 and Ile319, Phe322, Phe325, Tyr326, Val348, Phe349 and Pro352 for TLR2. In addition, the binding free energy of the complexes obtained using the MMGBSA analyzed using the Hawdock server for the protein-TLR2 and protein-TLR4 complexes was negative, indicating a stronger interaction between both complexes (Table 4).

**Table 4:** Analysis of the results of the molecular docking between the chimeric protein with TLR4 and TLR2 using Cluspro 2.0, HADDOCK and PRODIGY web servers.

Complex	ΔG (kcal mol-1)	Weighted score	HADDOCK score	Number of hydrogen bonds	number of hydrophobic bonds	PRODIGY (Kd)	MM/GBSA
**Vaccine candidate–TLR4**	−15.1	−1171.4	−130.7±6.6	20	17	8.2e-12	−35.1
**Vaccine candidate–TLR2**	−18.8	−1491.3	−202.7±5.5	21	18	1.6e-14	−70.44

### 3.11 Molecular dynamics simulation

**Fig. 5:**
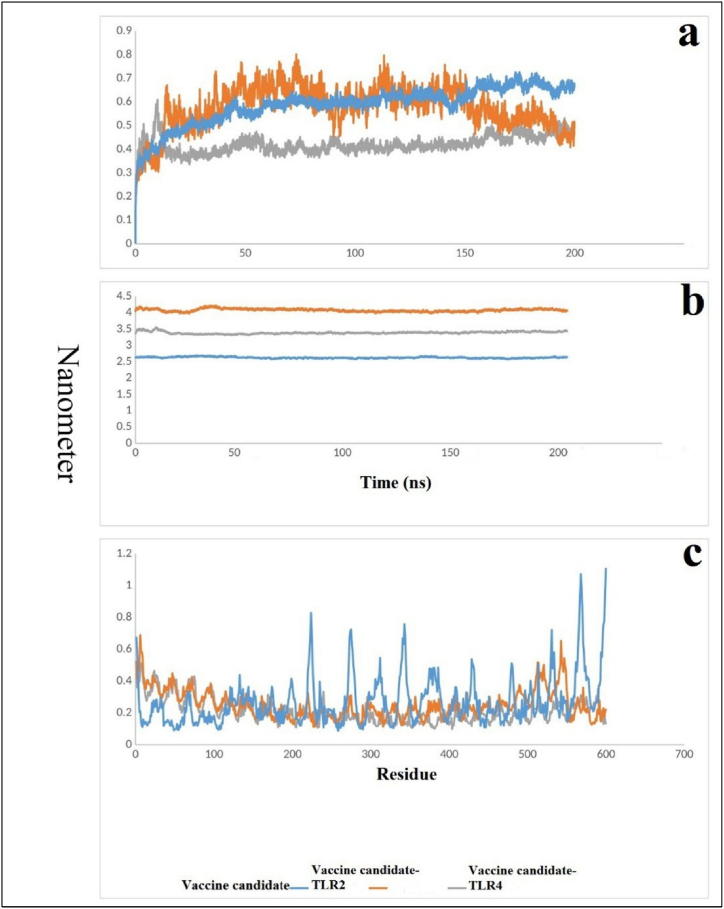
(a) Results of RMSD analysis for unbound chimeric protein, chimeric protein-TLR2 and chimeric protein-TLR4 complexes during simulation (ns). (B) Results of Rg analysis for unbound chimeric protein, chimeric protein-TLR2 and chimeric protein-TLR4 complexes during simulation (ns). (c) Results of RMSF analysis for unbound chimeric protein, chimeric protein-TLR2 and chimeric protein-TLR4 complexes during simulation (ns).

For a maximum of 200 ns, molecular dynamics simulations were run to confirm the stability of the unbound protein structure and protein receptor complexes. During the simulation, more stability and less volatility are shown by a lower RMSD. According to an analysis of the simulation data, the average RMSD of the developed protein was 0.58 nm, stabilizing at around 50 ns (Fig. 5a). This stability is preserved for the 200 ns of the simulation. Protein TLR4 and TLR2 complexes stabilized at around 20 ns as well. Although there were some minor variations in the protein TLR2 complex during the simulation, they were less than 0.3 nm, which suggests that the complex remained stable (Fig. 5a). The most stable structure during the experiment was that of the protein TLR4 complex, with an average RMSD of 0.42 nm. As seen in Fig. 5a, these findings suggest that the protein's interaction with immune system receptors is stable. We computed the parameter Rg during the MD simulation, which assesses the density change rate. With the help of this variable, we may examine the protein's general dimensions. Proteins and complexes are more stable the more stable the protein compression is during the simulation. Once the compression changes of the proposed protein were evaluated, both alone and in interaction with TLR4 and TLR2, during the simulation, the Rg analysis revealed that all three constructions were stable (Fig. 5b).

The structure's movement and flexibility were evaluated using RMSF calculations of the amino acids to check the changes in the backbone atoms of the unbonded protein and its interaction with TLR4 and TLR2. In this analysis, the average change of each residue during the simulation was plotted. As shown in Fig. 5c, compared to the designed protein, the RMSF values for most of the amino acids in the protein-TLR4 and protein-TLR2 complexes show small variations (less than 3. nm). These results indicate that the designed protein becomes more stable when interacting with the immune receptors.

**Fig. 6:**
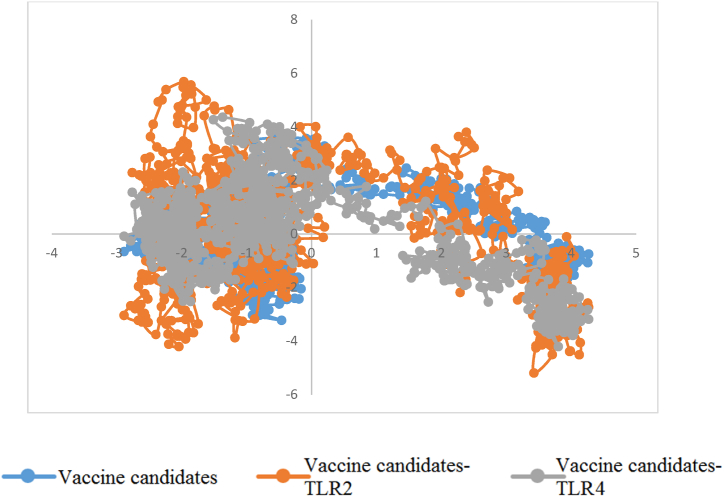
Results of conformational sampling in principal component analysis. Conformational sampling of the designed protein, protein-TLR2 and protein-TLR4 complexes is shown in the 2D projection of the trajectories.

**Fig. 7:**
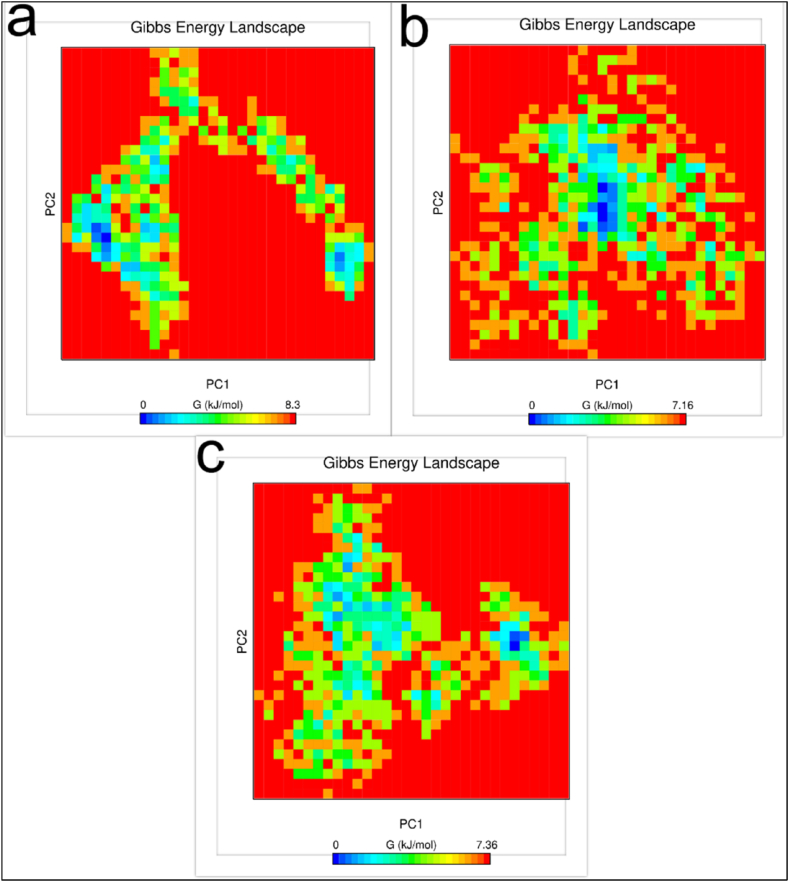
Results of Gibbs energy evaluation during the 200 ns simulation of the designed protein (a), the TLR2-protein complex (b) and the TLR4-protein complex (c).

**Fig. 8:**
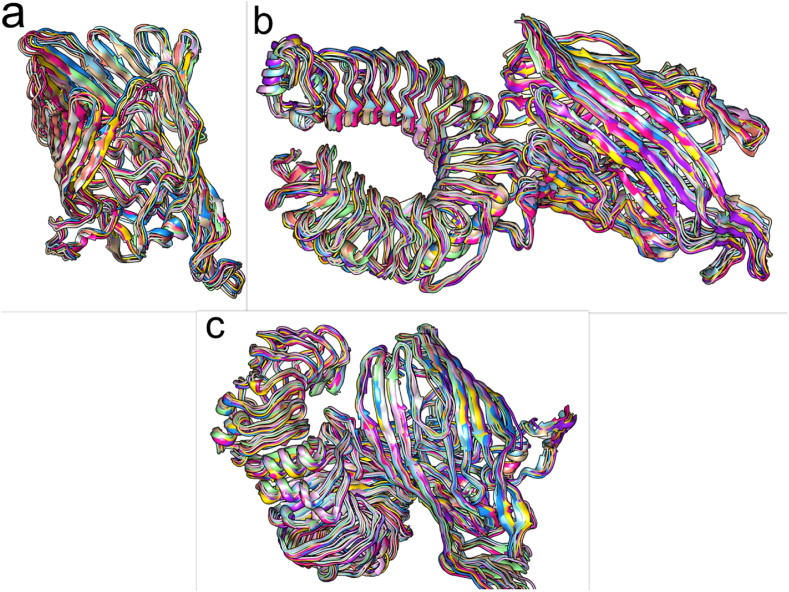
Snapshots of 0, 20, 40, 60, 80, 100, 120, 140, 160, 180, and 200 ns of MD simulation of the designed protein and ligand-receptor complexes. a) The designed protein, b) protein-TLR2, c) protein-TLR4 complexes. 0: light brown, 20: light blue, 40: light purple, 60: light green, 80: pink, 100: gray, 120: purple, 140: yellow, 160: blue, 180: purple, and 200: medium blue. During the simulation, the vaccine interaction site with the receptors was stable.

The major movements of atom pairs related to essential biological processes are computed by principal component analysis (PCA) using covariance matrices of Cα atoms. Chimeric protein-TLR2, chimeric protein-TLR4, and their first two principal components (PC1 and PC2) were created by projecting the trajectories onto the corresponding eigenvectors. Fig. 6 displays the three structures' PCA. The chimeric protein-TLR2 and chimeric protein-TLR4 complexes were found to occupy the majority of the shared essential subspace, as indicated by Fig. 6. The three structures were shown to have similar conformational subspaces in the EV plots. The stability of the complexes and potential vaccination in the simulation is demonstrated by the sampling of both systems. Additionally, the chimeric protein (Fig. 7a) exhibited a bigger global energy minimum (8.3 kJ/mol) than the chimeric protein-TLR2 (Fig. 7b) and chimeric protein-TLR4 (Fig. 7c) complexes (7.16 and 7.36 kJ/mol, respectively) (Fig. 7). These findings were supported by the FELs of the first and second PCA. This suggests that the chimeric protein has undergone more structural changes inside the designated region. These findings supported the RMSD, Rg, and RMSF value assessments in confirming a decrease in the complexes' overall flexibility. During the simulation, snapshots at 0, 20, 40, 60, 80, 100, 120, 140, 160, 180, and 200 ns intervals showed that the vaccine structure and receptor interaction site were constant (Fig. 8a–c).

### 3.12 Immune simulation

**Fig. 9:**
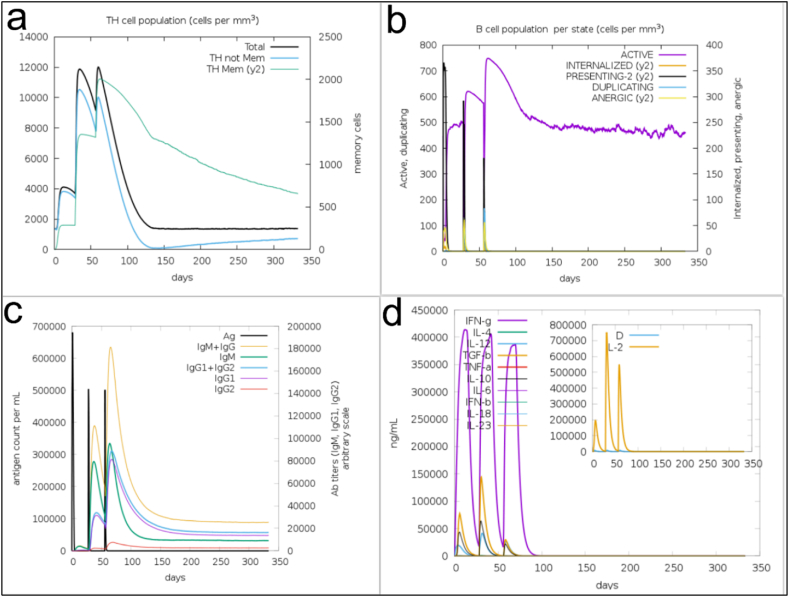
Results of the evaluation of the in silico immune simulation against the designed protein antigen using the C-ImmSim web server. The simulations are shown after three injections at step 1, step 84 and step 168. (A) TH cell population. (b) B cell population. (c) Antigen and immunoglobulin. (d) Cytokine production.

To model the immune system's reaction to the proposed vaccine candidate, the C-ImmSim server was utilized. Fig. 6 depicts the simulation of the host's immunological response to the protein used as a candidate for the vaccination. In this assessment, the TH cell and B cell populations, cytokine production, antigen, and immunoglobulin characteristics were all looked at. Elevated IgM levels signify the primary immune response of the host. Furthermore, elevated levels of the TH cell population (Fig. 9a), B cell population (Fig. 9b), and IgG1, IgG2 (Fig. 9c) suggest a subsequent response to the designed protein as antigen. Additionally, there was a notable rise in the concentrations of cytokines and interleukins following vaccination, particularly interferon γ (Fig. 9d). The analysis of these findings indicates that the vaccine candidate can activate the immune system to generate cytokines and antibodies targeting *A. baumannii*.

### 3.13 Codon optimization and in silico cloning of the designed candidate vaccine

**Fig. 10:**
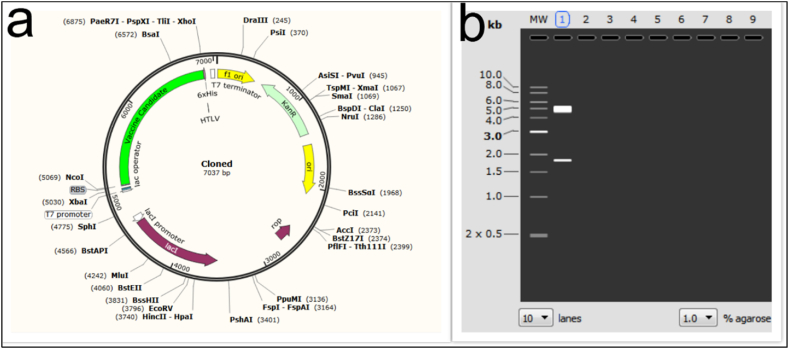
(a) The designed protein gene is cloned into pET-28a. The green part is the insertion site of the vaccine; (b) Double digestion with *Nco*I and *Xho*I enzymes to confirm cloning of the designed protein.

We optimized the codons using the JCat program. After codon optimization, the proposed structure had a sequence length of 1806 nucleotides. Before optimization, the nucleotide sequence had a GC content of 66.44 % and a codon compatibility index of 42.0 %. 1 and 52.27 %, respectively, after codon optimization. The sequence of the optimized vaccine candidate in pET-28a (+) was clonable in pET-28a (+) as demonstrated by the SnapGene software simulation (Fig. 10a). The presence of the pET-28a(+) vector (5231 bp) and the vaccine candidate (1806 bp) was verified by double digestion using the enzymes *Nco*I and *Xho*I (Fig. 10b).

## 4 Discussion

Infectious diseases are the second leading cause of death worldwide. In recent decades, *A. baumannii* has been increasingly implicated in nosocomial outbreaks as a cause of ventilator-associated pneumonia, bacteremia, and urinary tract infections, particularly in intensive care units [58]. The resistance of many pathogenic bacteria to antibiotics has led to interest in the production of vaccines to prevent these diseases [59].

Infection with *A. baumannii* is associated with increased morbidity and mortality and prolonged hospitalization [60]. Many investigators have explored routes to develop vaccines against *A. baumannii*, including inactivated whole cells, outer membrane vesicles, multiple bacterial antigens, rOmpA, and (1–6)-poly-N-acetyl-d-glucosamine (PNAG) [61]. No vaccine has been approved to provide effective protective immunity against this bacterium, despite all efforts.

Several innate immune cells have been identified as important factors in the defense against *A. baumannii*, including monocytes, macrophages, dendritic cells, and natural killer cells. Among these, neutrophils have emerged as a key immune cell essential for infection control [15]. One of the most important immune strategies used by immune cells to recognize this bacterium is pattern recognition receptors, particularly TLRs. Based on this strategy, bactericidal mechanisms generated by these receptors, including oxidative burst, increase cytokine and chemokine production against bacteria [15,62]. TLR-2 and TLR-4 are the two major TLRs that recognize PAMPs during bacterial infections and have been extensively studied and evaluated in *A. baumannii* infection [15,63,64]. Today, several proteins of *A. baumannii* are being introduced and investigated as vaccine candidates in many studies [8,65]. Dey and colleagues developed a multi-epitope subunit vaccine combining linear B lymphocytes (LBL), cytotoxic T lymphocytes (CTL) and helper T lymphocytes (HTL) from the protective LPS assembly proteins (LptE and LptD). The results showed that the their vaccine candidate can induce both B- and T-cell responses and elicit potent primary, secondary and tertiary immune responses [11]. The OmpA protein seems to be one of the best protein candidates for vaccine development due to its high immunogenicity in mice and its wide distribution as a virulence factor among many different strains of *A. baumannii* [66]. OmpA plays an important role in the pathogenic potential of the pathogen and is the most abundant surface protein in *A. baumannii* and also plays a role in biofilm formation. Clinical *A. baumannii* isolates with overexpression of OmpA show increased morbidity and even mortality in patients. Recently, the presence of OmpA in *A. baumannii* has been implicated in resistance to colistin, one of the last antibiotics effective against the bacterium [16]. One of the major challenges we face is carbapenem resistance in *A. baumannii*. The loss of an outer membrane protein called CarO (Carbapenem Resistance Outer Membrane Protein) is associated with carbapenem resistance in Acinetobacter baumannii. This is a membrane purine protein of *A. baumannii*. CarO is essential for the absorption of l-ornithine. l-ornithine reduces the susceptibility of *A. baumannii* to imipenem [67]. The Znu system is one of the transport systems responsible for Zn uptake in many Gram-negative bacteria. ZnuABC systems have been described in Gram-negative bacteria. ZnuD, which binds Zn with high affinity, is the best characterized Zn transporter in the outer membrane. This receptor is required for zinc uptake from the host cell and may be a good candidate for vaccine development [68,69].

Considering the importance of OmpA, ZnuD and CarO proteins in *A. baumannii* function, in this study we decided to design a vaccine consisting of domains rich in B and T cell epitopes that will ultimately protect these necessary and important proteins in case of human exposure to *A. baumannii*. The bacterium is targeted by the immune system and destroyed.

In the present study, the epitope prediction tools Bcpred, Rankpep, Bepipred and Ellipro were used to analyze OmpA, ZnuD and CarO proteins. Based on immunoinformatics analysis, 7 epitope-rich domains from these three proteins were finally selected containing B-cell and T-cell epitopes and linked with GGGGS linkers. The domains selected were regions of the proteins that were rich in epitopes common to both B cells and T cells. Ideally, epitope vaccines should contain B-cell epitopes that induce protective antibody responses, but also essential T-cell epitopes that stimulate Th responses. After the protein sequence was designed as a vaccine candidate, the physical and chemical parameters of the proteins, secondary and tertiary structure, solubility, allergenicity and antigenicity were analyzed. The I-TASSER server is used in various bioinformatics studies for the design of recombinant proteins. It uses the combined structure modeling methods including Threatening, Comparative Modeling and ab initio to predict the tertiary structure. The ProSA-web, SAVES, ERRAT, and MolPrabity servers are used to evaluate and validate the predicted structures [[70], [71], [72]]. MolProbity is a third-party structure validation server that greatly enhances the power and sensitivity of optimal hydrogen placement and all-atom contact analysis by providing a broad spectrum-based assessment of model quality for proteins and nucleic acids. And covalent geometry relies on torsion angle measurements. X-ray crystallography provides a wealth of biologically relevant molecular data in the form of three-dimensional atomic structures of proteins, nucleic acids, and large complexes in a variety of forms and states [73]. The results of our evaluations have shown that the recombinant protein obtained in the present study has the characteristics of a natural structure and is similar to proteins found in nature. Based on the Ramachandran curve, its structure has the desired stability. Today, bioinformatics studies are used for various purposes, including vaccine design and determining the characteristics of new genes. In this context, computational methods for predicting the formation of recombinant proteins and protein-protein reactions facilitate the analysis and design of recombinant proteins as vaccine candidates [74]. Further analysis of the designed protein sequence using VaxiJen and Allertop web servers showed that the desired protein is a strong antigen and is not an allergen for the human body. The physicochemical properties and tertiary structure of the designed protein were evaluated. Next, it was determined how the vaccine candidate interacted with the immune system's TLR2 and TLR4, which are crucial in the defense against *A. baumannii* infection. The proposed protein has an efficient binding to the TLR2 and TLR4 active sites, and it may be able to activate innate immune receptors to cause an active immune response, according to the analysis of the molecular interaction conducted with the Cluspro server. Using MD simulations up to 200 ns, the stability of the suggested protein structure was also examined both on its own and in conjunction with TLR2 and TLR4. The intended unbound protein and both protein-receptor complexes were stable throughout the simulation, according to an analysis of the RMSD data. Throughout the simulation, the structure of the protein-TLR4 combination was the most stable. To more precisely assess the stability of the structures, we assessed the degree of compression change during the MD simulation using an Rg analysis. Rg is commonly defined as the root mean square distance between an atom set and its common center of mass. The findings show that the chimeric protein effectively interacts with immune system receptors, especially TLR4. It is probably going to trigger a powerful defense against *A. baumannii* [75]. This variable so enables us to investigate the protein's general dimensions. The stability of the protein compression during the simulation provides a more compelling example of the complex's and the protein's stability. The findings of the simulation indicate that the fold modifications of the suggested protein do not vary when it is unbound or in a complex with TLR4 or TLR2. By examining the RMSF of amino acid residues, a structure's mobility and flexibility may be evaluated [76]. We also decide to carry out an RMSF analysis in order to look at the modifications to the backbone atoms of the chimeric protein-TLR4 and chimeric protein-TLR2 complexes as well as the modified protein. The RMSF values for most amino acids in the chimeric protein-TLR4 and chimeric protein-TLR2 complexes are somewhat different from the intended protein. The data clearly show that the protein design has resulted in an increased level of stability during interactions with the immune system receptors. According to the simulation performed with the C-ImmSim server, the produced vaccine candidate has the ability to induce the necessary immune response to fight *A. baumannii*. This is achieved by increasing the production of antibodies and cytokines in the body. While there are advantages to using immunoinformatics for vaccine development, there are also obstacles to overcome. One such challenge is the lack of a universally accepted, standardized method for accurately measuring vaccine efficacy using computational approaches. The development of numerical standards or metrics for evaluation may warrant funding for further investigation [77]. To date, there has been little discussion of the barriers *to* in silico vaccine development. Whether a multi-epitope vaccine initiated in silico can ultimately be approved by the FDA needs to be evaluated more carefully. Computational studies alone are purely theoretical. Finally, immunoinformatics methods offer many opportunities for new directions in healthcare. The diversity of existing work demonstrates its versatility [78]. The role of informatics in medical innovation has expanded. This is demonstrated by the abundance of genomic and proteomic data. Therefore, newer criteria should be considered to more accurately evaluate which designed structure is worthy of entering the laboratory [79]. According to the bioinformatics analysis of the vaccine design, this candidate is expected to be effective against *A. baumannii*. Immunoinformatics/computational methods generated raw data for research purposes. The consistency and sophistication of computer algorithms limit the accuracy of immunoinformatics predictions. Therefore, in vitro and in vivo experiments are needed to confirm the real potential of the designed vaccine candidate against *A. baumannii*.

## 5 Conclusion

In summary, we have analyzed the three *A. baumannii* proteins, OmpA, ZnuD and CarO, for the best immunogenic domains to induce a robust immune response. Finally, a protein vaccine candidate was created by selecting seven domains rich in B- and T-cell epitopes and linking them to the GGGGS linker. The immune system receptors TLR2 and TLR4, which are critical for defense against *A. baumannii*, significantly interact with the stabilized protein structure produced as a vaccine candidate. Effective defense against *A. baumannii* is provided by our engineered protein that is immunogenic and elicits the correct possible response.

## Data availability statement

Data included in article/supplementary material/referenced in article.

## CRediT authorship contribution statement

**Batul Negahdari:** Writing – original draft, Software, Methodology. **Parisa Sarkoohi:** Writing – review & editing, Project administration, Investigation, Conceptualization. **Forozan Ghasemi nezhad:** Software, Methodology. **Behzad Shahbazi:** Writing – review & editing, Writing – original draft, Software, Methodology, Investigation, Formal analysis. **Khadijeh Ahmadi:** Writing – review & editing, Writing – original draft, Software, Project administration, Methodology, Investigation, Formal analysis, Conceptualization.

## Declaration of competing interest

The authors declare that they have no known competing financial interests or personal relationships that could have appeared to influence the work reported in this paper.
