# Supplementary material for: Design of multi-epitope vaccine candidate based on OmpA, CarO and ZnuD proteins against multi-drug resistant Acinetobacter baumannii
Source: Heliyon. 2024 Jul 16;10(14):e34690. doi: 10.1016/j.heliyon.2024.e34690 (PMC11324976; doi:10.1016/j.heliyon.2024.e34690)
Supplement: Multimedia component 1 [file mmc1.docx]

**Table S1.** Selected Linear B cell Epitopes from *Acinetobacter baumannii* based on OmpA, CarO and ZnuD proteins

| **BCPred score** | **epitope** | **Position** | **Antigen** | **Server** |
| --- | --- | --- | --- | --- |
| 0.96  0.96  0.91  0.90  0.90 | ACINETACTERAMANN  YLDNDYGLERRAGQNA  VGTTGYGGAISYNANP  FGELGAYYTGNPTVNL  VGTTQINGTMKYKNDI | 31  177  106  239  205 | **CarO** | BCPred |
|  |  |  | **OmpA** |  |
| 0.94  0.92  0.91  0.90  0.89  0.89 | IQLISKTPTQNKIFTT  TIEAGEKNTYKSILGI  DVPGYTTLDFNAYWQM  ETDQDAPNRPRQNFNM  GGYGQTASVFIRGASA  GQIITEPAWWGGNPDL | 160  175  556  515  92  418 | **ZnuD** |  |

|  | **Antigen** | **Start position** | **End position** | **Peptide** |
| --- | --- | --- | --- | --- |
| IEDB (Kolaskar and Tongaonkar) | **CarO** | 4  34  62  110  135  160  181  199  4  22  35  53  114  164  182  4  22  37  53  114  164  182  4  22  37  53  114  164  182  4  23  37  53  114  168  186  198  4  22  37  53  114  170  188  200  4  22  32  53  114  149  170  188  200  4  23  56  95  113  169  187  199  218  4  23  56  95  113  169  187  199  218  4  22  37  53  114  164  182  4  23  37  53  114  168  186  198  16  77  112  133  151  4  22  37  53  114  149  170  188  200 | 16  47  70  121  141  173  187  206  16  30  43  68  124  170  188  17  30  45  68  123  170  188  16  30  43  68  123  170  188  17  30  43  68  123  174  192  207  17  30  45  68  123  178  194  211  16  30  45  68  123  154  176  194  211  16  29  68  100  125  175  193  209  223  16  29  68  100  125  175  193  209  223  17  30  43  68  123  170  188  17  30  43  68  123  174  192  207  31  89  117  139  157  17  30  43  68  123  154  176  194  212 | VRILLSAGILTAI  ATFSFLKPASVRAE  NPYVGVTLG  WFYVATGVAYLD  SFKVDGA  NDIVPYLGVGFSPA  FGELGAY  DAASLALI  LRVLVTTTALFAA  DEAVVHDSY  NQLIPVGAR  GALLWQANPYVGLALG  QGLYVAAGAAY  APYLGFG  FGEVGAY  LRVLVTTTALLAAG  DEAVVHDSY  LIPVGVRAE  GALLWQANPYVGLALG  QGLYIAAGAA  APYLGFG  FGEVGAY  LRVLVTTTALFAA  DEAVVHDSY  LIPVGAR  GALLWQANPYVGLALG  QGLYVAAGAA  APYLGFG  FGEVGAY  LRVLVTTTALLAAG  EAVVHDSY  LIPVGAR  GALLWQANPYVGLALG  QGLYVAAGAA  APYLGFG  FGEVGAY  TVKLVSSGSA  LRVLVTTTALLAAG  DEAVVHDSY  LIPVGVRAE  GALLWQANPYVGLALG  QGLYIAAGAA  APYVGLGFA  FGEVGAY  TVQLTQYNLAPV  LRVLVTTTALFAA  DEAVVHDSY  FDQKQLLPVGVRAE  GALLWQANPYVGLALG  QGLYVAAGAA  QQAVAG  APYVGFG  FGEVGAY  KVQLTQYNLAPV  LKVLAVATALSAS  DQVVQSG  LWTANPYVGLALG  LAYLNA  AQGLYVAAGVGYV  APYLGFG  FGEVGAY  SVQLVSSHPKL  GQAVID  LKVLAVATALSAS  DQVVQSG  LWTANPYVGLALG  LAYLNA  AQGLYVAAGVGYV  APYLGFG  FGEVGAY  SVQLVSSHPKL  GQAVID  LRVLVTTTALLAAG  DEAVVHDSY  LIPVGAR  GALLWQANPYVGLALG  QGLYVAAGAA  APYLGFG  FGEVGAY  YVFLVTTTALLAAG  EAVVHDSY  LIPVGAR  GALLWQANPYVGLALG  QGLYVAAGAA  APYLGFG  FGEVGAY  TVKLVSSGSA  GALLWQANPYVGLALG  QGLYIAAGAAYLD  QQAVPG  APYLGFG  FGEVGAY  LRVLVTTTALLAAG  DEAVVHDSY  LIPVGAR  GALLWQANPYVGLALG  QGLYIAAGAA  QQAVPG  APYLGFG  FGEVGAY  KVELTQYNLAPVT |
|  | **OmpA** | 4  56  118  187  247  290  338 | 34  68  132  220  257  298  346 | SRIALATMLVAAPLAAANAGVTVTPLLLGYT  QDDLFVGAALGIE  KIKPYVLLGAGHYKY  LAGLNVVLGGHLKPAAPVVEVAPVEPTPVAPQPQ  PEIAKVAEKLS  VKSALVNEY  SRTVVVQPG |
|  | **ZnuD** | 11  39  53  73  97  108  124  139  156  185  229  256  296  325  339  358  394  413  436  452  466  474  504  543  554  598 | 30  47  65  83  104  114  132  152  165  201  244  263  308  331  344  367  407  420  444  462  472  480  510  549  560  607 | PTRLVGAIAIAMGCSPVIFA  LDPIVITAS  ASEVPARISVIDE  IADLPSLLRKE  TASVFIRG  GHTLFLQ  VAGQNIHLF  QIEILRGPASVQYG  IGGVIQLISK  KSILGIDLAQDGYYAQI  SAKVGVDKEQYALSAE  SYGAAQAY  NLIKAAYPSIVNT  QNILFGV  EKVVAY  GYYLQHQYQH  GRFLVTPTTSVYAN  RAPVVGQI  EESVSYELG  GFNVYGSVYQT  NLMVSSA  TNFVFYN  AEYAYIQ  TAFVAKG  IQDVPGY  GSYYIASGRL |

|  | **Antigen** | **Start position** | **End position** | **Peptide** |
| --- | --- | --- | --- | --- |
| **Bepipred** | **CarO** | 26  74  125  143  157  173  196  218  26  31  73  107  128  144  174  200  23  73  107  126  144  174  200  25  73  128  199  24  72  107  125  162  177  203  220  25  108  125  148  180  205  74  106  132  180  203  22  74  106  129  150  161  179  203  22  74  106  129  150  161  179  203  24  73  107  128  144  174  199  26  74  106  132  164  177  199  220  37  69  94  111  126  143  169  31  74  106  134  180  203 | 41  92  136  150  161  177  214  229  27  39  86  112  141  162  178  226  40  88  113  140  162  178  226  38  87  162  226  38  86  108  153  166  182  217  230  39  108  139  158  183  233  86  113  158  184  233  36  85  113  140  159  166  182  232  36  85  113  140  159  166  182  232  39  88  112  141  162  178  226  39  85  113  155  166  182  218  230  51  76  104  121  132  147  175  38  87  113  159  184  234 | RIVTDEGVATFSFLKP  GDISWSDDLKINGSKYDLD  GLERRAGQNASF  FANPVGTT  KYKND  AITNR  LNADAASLALIGDDGRTLG  RAEERKIENKGT  VH  AFDKNQLIP  DISWRDDLSINGTK  ASTNRW  DYDLTKRSQDGTIK  GNNYNFNGSVDGKLSYKND  KINKN  NGTFVNVNGADFDKDLRAEENKIRNDD  EAVVHDSYAFDKNQLIPV  DISWRDDVSVNGTKYD  ASTNRWA  DYDLTKRSQDGTI  GNNYSFNGSVDGKLSYKND  KINKN  NGTFVNANGADFDKDLRAEENKIRNDD  VVHDSYAFDKNQLI  DISWTDDVSVNGTKY  DYDLTKRSQDGTIKVNGNNYNFNGSVDGKLSYKND  SNGTFVNVNGADFDKDLRAEENEIRNDD  AVVHDSYAFDKNQLI  GDISWSDDVKVNGST  AS  LDNDYDLTRNVDATRSFRVNNQDFIAGAD  SYKND  PKINKN  SSGSAVTTGDQSLEE  NAEARKIANDD  VVHDSYAFDKNQLIP  S  LDNDYDLAKRIGNGG  YQQAAAGQEGG  KINK  QYNLAPVTGNTTLPQDAVDKEANKIRNDD  ISWTDDVSVNGTK  GASSNPWA  AKRIGNGETLSIDGKNYQQAVAGQEGG  KINKN  LTQYNLAPVNGNPTSAQDAVDKEENEIRNDD  DDQVVQSGYAFDKNQ  ISWSDDIKVNGS  GASQNVWA  YDLKRNIGTNAS  AGLNDNVSIK  SQQYDN  KITK  VSSHPKLIGDDGRTLGQAVIDEANKIENDD  DDQVVQSGYAFDKNQ  ISWSDDIKVNGS  GASQNVWA  YDLKRNIGTNAS  AGLNDNVSIK  SQQYDN  KITK  VSSHPKLIGDDGRTLGQAVIDEANKIENDD  AVVHDSYAFDKNQLIP  DISWRDDLSINGTKYD  ASTNRW  DYDLTKRSQDGTIK  GNNYNFNGSVDGKLSYKND  KINKN  SNGTFVNVNGADFDKDLRAEENKIRNDD  VHDSYAFDKNQLIP  ISWSDDVKVNGS  GASTNRWA  TRNVDATRSFRVNNQDFIAGADGV  KND  PKINKN  VKLVSSGSAVTTGDQSLEEA  NAEARKIANDD  ISWTDDVSVNGTKYD  GASTNPWA  LAKRIGNGDTL  YQQAVPGQEGG  MSYKNDI  KISKN  YNLAPVT  AFDKNQLI  ISWTDDVSVNGTKY  GASTNPWA  RIGNGDTLSIDGKNYQQAVPGQEGGV  KISKN  LTQYNLAPVTGNPTSAKDAVDKEANEIRNDNK |
|  | **OmpA** | 24  35  80  113  132  175  192  234  272  299  310  339 | 25  59  99  117  149  185  225  248  282  302  326  352 | VT  FQDSQHNNGGKDGNLTNGPELQDDL  NQVKGDVDGASAGAEYKQKQ  KNYDS  YDFDGVNRGTRGTSEEGT  YNADEEFWNYT  VVLGGHLKPAAPVVEVAPVEPTPVAPQPQELTED  FDTNKSNIKDQYKPE  NTGPRKLNERL  NVDA  FAWDQPIADNKTKEGRA  RTVVVQPGQEAAAP |
|  | **ZnuD** | 5  30  49  66  73  88  120  147  168  177  201  232  245  276  279  290  335  365  379  414  448  473  495  514  534  548  569  589 | 10  38  63  70  74  97  135  153  173  198  230  240  265  277  281  323  353  369  388  435  452  480  499  526  540  559  572  603 | MSKSFQ  AEDATNATQ  SAEKASEVPARISVI  KTIEQ  IA  IHQTGGYGQT  NTATVAGQNIHLFDTT  ASVQYGT  TQNKIF  EAGEKNTYKSILGIDLAQDGYY  IRGQRFETDGDQIISNDDRKAGFDQKGYSA  VGVDKEQYA  IKENKGTGDFFSYGAAQAYDF  LN  TNN  QFEDQYNLIKAAYPSIVNTKQQEVDSNIKWQFTP  KTTAEKVVAYNGDTGFDNS  YQHDG  EDNDQFGTHT  APVVGQIITEPAWWGGNPDLKP  KLNHG  ATNFVFYN  WSLDN  DETDQDAPNRPRQ  WDNGVYG  KGKAKDIQDVPG  WQMS  YKTAWNKEVGSYYIA |
