# Supplementary material for: Design of multi-epitope vaccine candidate based on OmpA, CarO and ZnuD proteins against multi-drug resistant Acinetobacter baumannii
Source: Heliyon. 2024 Jul 16;10(14):e34690. doi: 10.1016/j.heliyon.2024.e34690 (PMC11324976; doi:10.1016/j.heliyon.2024.e34690)
Supplement: Multimedia component 3 [file mmc3.docx]

**Table S3.** Toxicity evaluation results of designed protein epitopes using the Toxinpred web server.

| **Peptides Scanned from Original Protein** | | | | |  | | | | | | | | | | | | | | | | | | | | |
| --- | --- | --- | --- | --- | --- | --- | --- | --- | --- | --- | --- | --- | --- | --- | --- | --- | --- | --- | --- | --- | --- | --- | --- | --- | --- |
| [**Peptide Sequence**](https://webs.iiitd.edu.in/raghava/toxinpred/prot_submitfreq_S.php?ran=87500) | [**SVM score**](https://webs.iiitd.edu.in/raghava/toxinpred/prot_submitfreq_S.php?ran=87500) | [**Prediction**](https://webs.iiitd.edu.in/raghava/toxinpred/prot_submitfreq_S.php?ran=87500) | [**Hydrophobicity**](https://webs.iiitd.edu.in/raghava/toxinpred/prot_submitfreq_S.php?ran=87500) | [**Hydropathicity**](https://webs.iiitd.edu.in/raghava/toxinpred/prot_submitfreq_S.php?ran=87500) | [**Hydrophilicity**](https://webs.iiitd.edu.in/raghava/toxinpred/prot_submitfreq_S.php?ran=87500) | [**Charge**](https://webs.iiitd.edu.in/raghava/toxinpred/prot_submitfreq_S.php?ran=87500) | [**Mol wt**](https://webs.iiitd.edu.in/raghava/toxinpred/prot_submitfreq_S.php?ran=87500) |  |  |  |  |  |  |  |  |  |  |  |  |  |  |  |  |  |  |
| [DEAVVHDSYA](https://webs.iiitd.edu.in/raghava/toxinpred/pepsearch_S.php?seq=DEAVVHDSYA&thval=0.0) | -1.18 | Non-Toxin | -0.11 | -0.38 | 0.25 | -2.50 | 1105.25 |  |  |  |  |  |  |  |  |  |  |  |  |  |  |  |  |  |  |
| [EAVVHDSYAF](https://webs.iiitd.edu.in/raghava/toxinpred/pepsearch_S.php?seq=EAVVHDSYAF&thval=0.0) | -1.41 | Non-Toxin | 0.02 | 0.25 | -0.30 | -1.50 | 1137.34 |  |  |  |  |  |  |  |  |  |  |  |  |  |  |  |  |  |  |
| [AVVHDSYAFD](https://webs.iiitd.edu.in/raghava/toxinpred/pepsearch_S.php?seq=AVVHDSYAFD&thval=0.0) | -1.42 | Non-Toxin | 0.01 | 0.25 | -0.30 | -1.50 | 1123.31 |  |  |  |  |  |  |  |  |  |  |  |  |  |  |  |  |  |  |
| [VVHDSYAFDK](https://webs.iiitd.edu.in/raghava/toxinpred/pepsearch_S.php?seq=VVHDSYAFDK&thval=0.0) | -1.25 | Non-Toxin | -0.12 | -0.32 | 0.05 | -0.50 | 1180.41 |  |  |  |  |  |  |  |  |  |  |  |  |  |  |  |  |  |  |
| [VHDSYAFDKN](https://webs.iiitd.edu.in/raghava/toxinpred/pepsearch_S.php?seq=VHDSYAFDKN&thval=0.0) | -1.18 | Non-Toxin | -0.24 | -1.09 | 0.22 | -0.50 | 1195.38 |  |  |  |  |  |  |  |  |  |  |  |  |  |  |  |  |  |  |
| [HDSYAFDKNQ](https://webs.iiitd.edu.in/raghava/toxinpred/pepsearch_S.php?seq=HDSYAFDKNQ&thval=0.0) | -1.26 | Non-Toxin | -0.37 | -1.86 | 0.39 | -0.50 | 1224.38 |  |  |  |  |  |  |  |  |  |  |  |  |  |  |  |  |  |  |
| [DSYAFDKNQL](https://webs.iiitd.edu.in/raghava/toxinpred/pepsearch_S.php?seq=DSYAFDKNQL&thval=0.0) | -1.27 | Non-Toxin | -0.27 | -1.16 | 0.26 | -1.00 | 1200.40 |  |  |  |  |  |  |  |  |  |  |  |  |  |  |  |  |  |  |
| [SYAFDKNQLI](https://webs.iiitd.edu.in/raghava/toxinpred/pepsearch_S.php?seq=SYAFDKNQLI&thval=0.0) | -1.37 | Non-Toxin | -0.13 | -0.36 | -0.22 | 0.00 | 1198.48 |  |  |  |  |  |  |  |  |  |  |  |  |  |  |  |  |  |  |
| [YAFDKNQLIP](https://webs.iiitd.edu.in/raghava/toxinpred/pepsearch_S.php?seq=YAFDKNQLIP&thval=0.0) | -1.29 | Non-Toxin | -0.11 | -0.44 | -0.25 | 0.00 | 1208.52 |  |  |  |  |  |  |  |  |  |  |  |  |  |  |  |  |  |  |
| [AFDKNQLIPV](https://webs.iiitd.edu.in/raghava/toxinpred/pepsearch_S.php?seq=AFDKNQLIPV&thval=0.0) | -1.26 | Non-Toxin | -0.06 | 0.11 | -0.17 | 0.00 | 1144.48 |  |  |  |  |  |  |  |  |  |  |  |  |  |  |  |  |  |  |
| [FDKNQLIPVG](https://webs.iiitd.edu.in/raghava/toxinpred/pepsearch_S.php?seq=FDKNQLIPVG&thval=0.0) | -1.31 | Non-Toxin | -0.06 | -0.11 | -0.12 | 0.00 | 1130.46 |  |  |  |  |  |  |  |  |  |  |  |  |  |  |  |  |  |  |
| [DKNQLIPVGV](https://webs.iiitd.edu.in/raghava/toxinpred/pepsearch_S.php?seq=DKNQLIPVGV&thval=0.0) | -1.41 | Non-Toxin | -0.07 | 0.03 | -0.02 | 0.00 | 1082.42 |  |  |  |  |  |  |  |  |  |  |  |  |  |  |  |  |  |  |
| [KNQLIPVGVR](https://webs.iiitd.edu.in/raghava/toxinpred/pepsearch_S.php?seq=KNQLIPVGVR&thval=0.0) | -1.48 | Non-Toxin | -0.18 | -0.07 | -0.02 | 2.00 | 1123.52 |  |  |  |  |  |  |  |  |  |  |  |  |  |  |  |  |  |  |
| [NQLIPVGVRA](https://webs.iiitd.edu.in/raghava/toxinpred/pepsearch_S.php?seq=NQLIPVGVRA&thval=0.0) | -1.42 | Non-Toxin | -0.04 | 0.50 | -0.37 | 1.00 | 1066.42 |  |  |  |  |  |  |  |  |  |  |  |  |  |  |  |  |  |  |
| [QLIPVGVRAE](https://webs.iiitd.edu.in/raghava/toxinpred/pepsearch_S.php?seq=QLIPVGVRAE&thval=0.0) | -1.24 | Non-Toxin | -0.04 | 0.50 | -0.09 | 0.00 | 1081.43 |  |  |  |  |  |  |  |  |  |  |  |  |  |  |  |  |  |  |
| [LIPVGVRAEV](https://webs.iiitd.edu.in/raghava/toxinpred/pepsearch_S.php?seq=LIPVGVRAEV&thval=0.0) | -1.17 | Non-Toxin | 0.08 | 1.27 | -0.26 | 0.00 | 1052.43 |  |  |  |  |  |  |  |  |  |  |  |  |  |  |  |  |  |  |
| [IPVGVRAEVG](https://webs.iiitd.edu.in/raghava/toxinpred/pepsearch_S.php?seq=IPVGVRAEVG&thval=0.0) | -1.28 | Non-Toxin | 0.05 | 0.85 | -0.08 | 0.00 | 996.32 |  |  |  |  |  |  |  |  |  |  |  |  |  |  |  |  |  |  |
| [PVGVRAEVGT](https://webs.iiitd.edu.in/raghava/toxinpred/pepsearch_S.php?seq=PVGVRAEVGT&thval=0.0) | -1.21 | Non-Toxin | -0.04 | 0.33 | 0.06 | 0.00 | 984.26 |  |  |  |  |  |  |  |  |  |  |  |  |  |  |  |  |  |  |
| [VGVRAEVGTT](https://webs.iiitd.edu.in/raghava/toxinpred/pepsearch_S.php?seq=VGVRAEVGTT&thval=0.0) | -0.91 | Non-Toxin | -0.05 | 0.42 | 0.02 | 0.00 | 988.25 |  |  |  |  |  |  |  |  |  |  |  |  |  |  |  |  |  |  |
| [GVRAEVGTTG](https://webs.iiitd.edu.in/raghava/toxinpred/pepsearch_S.php?seq=GVRAEVGTTG&thval=0.0) | -0.85 | Non-Toxin | -0.09 | -0.04 | 0.17 | 0.00 | 946.17 |  |  |  |  |  |  |  |  |  |  |  |  |  |  |  |  |  |  |
| [VRAEVGTTGY](https://webs.iiitd.edu.in/raghava/toxinpred/pepsearch_S.php?seq=VRAEVGTTGY&thval=0.0) | -0.56 | Non-Toxin | -0.11 | -0.13 | -0.06 | 0.00 | 1052.29 |  |  |  |  |  |  |  |  |  |  |  |  |  |  |  |  |  |  |
| [RAEVGTTGYG](https://webs.iiitd.edu.in/raghava/toxinpred/pepsearch_S.php?seq=RAEVGTTGYG&thval=0.0) | -0.48 | Non-Toxin | -0.14 | -0.59 | 0.09 | 0.00 | 1010.21 |  |  |  |  |  |  |  |  |  |  |  |  |  |  |  |  |  |  |
| [AEVGTTGYGG](https://webs.iiitd.edu.in/raghava/toxinpred/pepsearch_S.php?seq=AEVGTTGYGG&thval=0.0) | -0.55 | Non-Toxin | 0.05 | -0.18 | -0.21 | -1.00 | 911.08 |  |  |  |  |  |  |  |  |  |  |  |  |  |  |  |  |  |  |
| [EVGTTGYGGA](https://webs.iiitd.edu.in/raghava/toxinpred/pepsearch_S.php?seq=EVGTTGYGGA&thval=0.0) | -0.64 | Non-Toxin | 0.05 | -0.18 | -0.21 | -1.00 | 911.08 |  |  |  |  |  |  |  |  |  |  |  |  |  |  |  |  |  |  |
| [VGTTGYGGAL](https://webs.iiitd.edu.in/raghava/toxinpred/pepsearch_S.php?seq=VGTTGYGGAL&thval=0.0) | -0.43 | Non-Toxin | 0.16 | 0.55 | -0.69 | 0.00 | 895.13 |  |  |  |  |  |  |  |  |  |  |  |  |  |  |  |  |  |  |
| [GTTGYGGALL](https://webs.iiitd.edu.in/raghava/toxinpred/pepsearch_S.php?seq=GTTGYGGALL&thval=0.0) | -0.38 | Non-Toxin | 0.16 | 0.51 | -0.72 | 0.00 | 909.16 |  |  |  |  |  |  |  |  |  |  |  |  |  |  |  |  |  |  |
| [TTGYGGALLW](https://webs.iiitd.edu.in/raghava/toxinpred/pepsearch_S.php?seq=TTGYGGALLW&thval=0.0) | -0.43 | Non-Toxin | 0.18 | 0.46 | -1.06 | 0.00 | 1038.32 |  |  |  |  |  |  |  |  |  |  |  |  |  |  |  |  |  |  |
| [TGYGGALLWQ](https://webs.iiitd.edu.in/raghava/toxinpred/pepsearch_S.php?seq=TGYGGALLWQ&thval=0.0) | -0.50 | Non-Toxin | 0.13 | 0.18 | -1.00 | 0.00 | 1065.35 |  |  |  |  |  |  |  |  |  |  |  |  |  |  |  |  |  |  |
| [GYGGALLWQA](https://webs.iiitd.edu.in/raghava/toxinpred/pepsearch_S.php?seq=GYGGALLWQA&thval=0.0) | -0.67 | Non-Toxin | 0.17 | 0.43 | -1.01 | 0.00 | 1035.32 |  |  |  |  |  |  |  |  |  |  |  |  |  |  |  |  |  |  |
| [YGGALLWQAN](https://webs.iiitd.edu.in/raghava/toxinpred/pepsearch_S.php?seq=YGGALLWQAN&thval=0.0) | -0.74 | Non-Toxin | 0.09 | 0.12 | -0.99 | 0.00 | 1092.37 |  |  |  |  |  |  |  |  |  |  |  |  |  |  |  |  |  |  |
| [GGALLWQANP](https://webs.iiitd.edu.in/raghava/toxinpred/pepsearch_S.php?seq=GGALLWQANP&thval=0.0) | -0.57 | Non-Toxin | 0.09 | 0.09 | -0.76 | 0.00 | 1026.31 |  |  |  |  |  |  |  |  |  |  |  |  |  |  |  |  |  |  |
| [GALLWQANPY](https://webs.iiitd.edu.in/raghava/toxinpred/pepsearch_S.php?seq=GALLWQANPY&thval=0.0) | -0.50 | Non-Toxin | 0.07 | -0.00 | -0.99 | 0.00 | 1132.43 |  |  |  |  |  |  |  |  |  |  |  |  |  |  |  |  |  |  |
| [ALLWQANPYV](https://webs.iiitd.edu.in/raghava/toxinpred/pepsearch_S.php?seq=ALLWQANPYV&thval=0.0) | -0.61 | Non-Toxin | 0.11 | 0.46 | -1.14 | 0.00 | 1174.51 |  |  |  |  |  |  |  |  |  |  |  |  |  |  |  |  |  |  |
| [LLWQANPYVG](https://webs.iiitd.edu.in/raghava/toxinpred/pepsearch_S.php?seq=LLWQANPYVG&thval=0.0) | -0.80 | Non-Toxin | 0.10 | 0.24 | -1.09 | 0.00 | 1160.49 |  |  |  |  |  |  |  |  |  |  |  |  |  |  |  |  |  |  |
| [LWQANPYVGL](https://webs.iiitd.edu.in/raghava/toxinpred/pepsearch_S.php?seq=LWQANPYVGL&thval=0.0) | -0.75 | Non-Toxin | 0.10 | 0.24 | -1.09 | 0.00 | 1160.49 |  |  |  |  |  |  |  |  |  |  |  |  |  |  |  |  |  |  |
| [WQANPYVGLA](https://webs.iiitd.edu.in/raghava/toxinpred/pepsearch_S.php?seq=WQANPYVGLA&thval=0.0) | -1.00 | Non-Toxin | 0.07 | 0.04 | -0.96 | 0.00 | 1118.40 |  |  |  |  |  |  |  |  |  |  |  |  |  |  |  |  |  |  |
| [QANPYVGLAL](https://webs.iiitd.edu.in/raghava/toxinpred/pepsearch_S.php?seq=QANPYVGLAL&thval=0.0) | -0.92 | Non-Toxin | 0.09 | 0.51 | -0.80 | 0.00 | 1045.35 |  |  |  |  |  |  |  |  |  |  |  |  |  |  |  |  |  |  |
| [ANPYVGLALG](https://webs.iiitd.edu.in/raghava/toxinpred/pepsearch_S.php?seq=ANPYVGLALG&thval=0.0) | -0.85 | Non-Toxin | 0.17 | 0.82 | -0.82 | 0.00 | 974.27 |  |  |  |  |  |  |  |  |  |  |  |  |  |  |  |  |  |  |
| [NPYVGLALGY](https://webs.iiitd.edu.in/raghava/toxinpred/pepsearch_S.php?seq=NPYVGLALGY&thval=0.0) | -0.86 | Non-Toxin | 0.15 | 0.51 | -1.00 | 0.00 | 1066.37 |  |  |  |  |  |  |  |  |  |  |  |  |  |  |  |  |  |  |
| [PYVGLALGYN](https://webs.iiitd.edu.in/raghava/toxinpred/pepsearch_S.php?seq=PYVGLALGYN&thval=0.0) | -1.19 | Non-Toxin | 0.15 | 0.51 | -1.00 | 0.00 | 1066.37 |  |  |  |  |  |  |  |  |  |  |  |  |  |  |  |  |  |  |
| [YVGLALGYNG](https://webs.iiitd.edu.in/raghava/toxinpred/pepsearch_S.php?seq=YVGLALGYNG&thval=0.0) | -1.07 | Non-Toxin | 0.17 | 0.63 | -1.00 | 0.00 | 1026.31 |  |  |  |  |  |  |  |  |  |  |  |  |  |  |  |  |  |  |
| [VGLALGYNGG](https://webs.iiitd.edu.in/raghava/toxinpred/pepsearch_S.php?seq=VGLALGYNGG&thval=0.0) | -0.92 | Non-Toxin | 0.19 | 0.72 | -0.77 | 0.00 | 920.19 |  |  |  |  |  |  |  |  |  |  |  |  |  |  |  |  |  |  |
| [GLALGYNGGD](https://webs.iiitd.edu.in/raghava/toxinpred/pepsearch_S.php?seq=GLALGYNGGD&thval=0.0) | -0.75 | Non-Toxin | 0.06 | -0.05 | -0.32 | -1.00 | 936.14 |  |  |  |  |  |  |  |  |  |  |  |  |  |  |  |  |  |  |
| [LALGYNGGDI](https://webs.iiitd.edu.in/raghava/toxinpred/pepsearch_S.php?seq=LALGYNGGDI&thval=0.0) | -0.83 | Non-Toxin | 0.12 | 0.44 | -0.50 | -1.00 | 992.25 |  |  |  |  |  |  |  |  |  |  |  |  |  |  |  |  |  |  |
| [ALGYNGGDIS](https://webs.iiitd.edu.in/raghava/toxinpred/pepsearch_S.php?seq=ALGYNGGDIS&thval=0.0) | -0.85 | Non-Toxin | 0.04 | -0.02 | -0.29 | -1.00 | 966.16 |  |  |  |  |  |  |  |  |  |  |  |  |  |  |  |  |  |  |
| [LGYNGGDISW](https://webs.iiitd.edu.in/raghava/toxinpred/pepsearch_S.php?seq=LGYNGGDISW&thval=0.0) | -1.01 | Non-Toxin | 0.05 | -0.29 | -0.58 | -1.00 | 1081.30 |  |  |  |  |  |  |  |  |  |  |  |  |  |  |  |  |  |  |
| [GYNGGDISWR](https://webs.iiitd.edu.in/raghava/toxinpred/pepsearch_S.php?seq=GYNGGDISWR&thval=0.0) | -0.81 | Non-Toxin | -0.18 | -1.12 | -0.10 | 0.00 | 1124.32 |  |  |  |  |  |  |  |  |  |  |  |  |  |  |  |  |  |  |
| [YNGGDISWRD](https://webs.iiitd.edu.in/raghava/toxinpred/pepsearch_S.php?seq=YNGGDISWRD&thval=0.0) | -1.05 | Non-Toxin | -0.27 | -1.43 | 0.20 | -1.00 | 1182.35 |  |  |  |  |  |  |  |  |  |  |  |  |  |  |  |  |  |  |
| [NGGDISWRDD](https://webs.iiitd.edu.in/raghava/toxinpred/pepsearch_S.php?seq=NGGDISWRDD&thval=0.0) | -1.03 | Non-Toxin | -0.34 | -1.65 | 0.73 | -2.00 | 1134.26 |  |  |  |  |  |  |  |  |  |  |  |  |  |  |  |  |  |  |
| [GGDISWRDDV](https://webs.iiitd.edu.in/raghava/toxinpred/pepsearch_S.php?seq=GGDISWRDDV&thval=0.0) | -1.17 | Non-Toxin | -0.22 | -0.88 | 0.56 | -2.00 | 1119.29 |  |  |  |  |  |  |  |  |  |  |  |  |  |  |  |  |  |  |
| [GDISWRDDVS](https://webs.iiitd.edu.in/raghava/toxinpred/pepsearch_S.php?seq=GDISWRDDVS&thval=0.0) | -1.19 | Non-Toxin | -0.26 | -0.92 | 0.59 | -2.00 | 1149.31 |  |  |  |  |  |  |  |  |  |  |  |  |  |  |  |  |  |  |
| [DISWRDDVSV](https://webs.iiitd.edu.in/raghava/toxinpred/pepsearch_S.php?seq=DISWRDDVSV&thval=0.0) | -1.16 | Non-Toxin | -0.23 | -0.46 | 0.44 | -2.00 | 1191.39 |  |  |  |  |  |  |  |  |  |  |  |  |  |  |  |  |  |  |
| [ISWRDDVSVN](https://webs.iiitd.edu.in/raghava/toxinpred/pepsearch_S.php?seq=ISWRDDVSVN&thval=0.0) | -1.18 | Non-Toxin | -0.22 | -0.46 | 0.16 | -1.00 | 1190.41 |  |  |  |  |  |  |  |  |  |  |  |  |  |  |  |  |  |  |
| [SWRDDVSVNG](https://webs.iiitd.edu.in/raghava/toxinpred/pepsearch_S.php?seq=SWRDDVSVNG&thval=0.0) | -1.06 | Non-Toxin | -0.27 | -0.95 | 0.34 | -1.00 | 1134.30 |  |  |  |  |  |  |  |  |  |  |  |  |  |  |  |  |  |  |
| [WRDDVSVNGT](https://webs.iiitd.edu.in/raghava/toxinpred/pepsearch_S.php?seq=WRDDVSVNGT&thval=0.0) | -0.88 | Non-Toxin | -0.27 | -0.94 | 0.27 | -1.00 | 1148.33 |  |  |  |  |  |  |  |  |  |  |  |  |  |  |  |  |  |  |
| [RDDVSVNGTY](https://webs.iiitd.edu.in/raghava/toxinpred/pepsearch_S.php?seq=RDDVSVNGTY&thval=0.0) | -0.88 | Non-Toxin | -0.30 | -0.98 | 0.38 | -1.00 | 1125.29 |  |  |  |  |  |  |  |  |  |  |  |  |  |  |  |  |  |  |
| [DDVSVNGTYI](https://webs.iiitd.edu.in/raghava/toxinpred/pepsearch_S.php?seq=DDVSVNGTYI&thval=0.0) | -0.75 | Non-Toxin | -0.05 | -0.08 | -0.10 | -2.00 | 1082.27 |  |  |  |  |  |  |  |  |  |  |  |  |  |  |  |  |  |  |
| [DVSVNGTYIA](https://webs.iiitd.edu.in/raghava/toxinpred/pepsearch_S.php?seq=DVSVNGTYIA&thval=0.0) | -0.70 | Non-Toxin | 0.04 | 0.45 | -0.45 | -1.00 | 1038.26 |  |  |  |  |  |  |  |  |  |  |  |  |  |  |  |  |  |  |
| [VSVNGTYIAA](https://webs.iiitd.edu.in/raghava/toxinpred/pepsearch_S.php?seq=VSVNGTYIAA&thval=0.0) | -0.75 | Non-Toxin | 0.14 | 0.98 | -0.80 | 0.00 | 994.25 |  |  |  |  |  |  |  |  |  |  |  |  |  |  |  |  |  |  |
| [SVNGTYIAAG](https://webs.iiitd.edu.in/raghava/toxinpred/pepsearch_S.php?seq=SVNGTYIAAG&thval=0.0) | -0.64 | Non-Toxin | 0.10 | 0.52 | -0.65 | 0.00 | 952.17 |  |  |  |  |  |  |  |  |  |  |  |  |  |  |  |  |  |  |
| [VNGTYIAAGA](https://webs.iiitd.edu.in/raghava/toxinpred/pepsearch_S.php?seq=VNGTYIAAGA&thval=0.0) | -0.63 | Non-Toxin | 0.15 | 0.78 | -0.73 | 0.00 | 936.17 |  |  |  |  |  |  |  |  |  |  |  |  |  |  |  |  |  |  |
| [NGTYIAAGAA](https://webs.iiitd.edu.in/raghava/toxinpred/pepsearch_S.php?seq=NGTYIAAGAA&thval=0.0) | -0.76 | Non-Toxin | 0.12 | 0.54 | -0.63 | 0.00 | 908.11 |  |  |  |  |  |  |  |  |  |  |  |  |  |  |  |  |  |  |
| [GTYIAAGAAY](https://webs.iiitd.edu.in/raghava/toxinpred/pepsearch_S.php?seq=GTYIAAGAAY&thval=0.0) | -0.79 | Non-Toxin | 0.19 | 0.76 | -0.88 | 0.00 | 957.18 |  |  |  |  |  |  |  |  |  |  |  |  |  |  |  |  |  |  |
| [TYIAAGAAYL](https://webs.iiitd.edu.in/raghava/toxinpred/pepsearch_S.php?seq=TYIAAGAAYL&thval=0.0) | -1.04 | Non-Toxin | 0.23 | 1.18 | -1.06 | 0.00 | 1013.29 |  |  |  |  |  |  |  |  |  |  |  |  |  |  |  |  |  |  |
| [YIAAGAAYLD](https://webs.iiitd.edu.in/raghava/toxinpred/pepsearch_S.php?seq=YIAAGAAYLD&thval=0.0) | -1.17 | Non-Toxin | 0.17 | 0.90 | -0.72 | -1.00 | 1027.27 |  |  |  |  |  |  |  |  |  |  |  |  |  |  |  |  |  |  |
| [IAAGAAYLDN](https://webs.iiitd.edu.in/raghava/toxinpred/pepsearch_S.php?seq=IAAGAAYLDN&thval=0.0) | -1.23 | Non-Toxin | 0.11 | 0.68 | -0.47 | -1.00 | 978.20 |  |  |  |  |  |  |  |  |  |  |  |  |  |  |  |  |  |  |
| [AAGAAYLDND](https://webs.iiitd.edu.in/raghava/toxinpred/pepsearch_S.php?seq=AAGAAYLDND&thval=0.0) | -1.21 | Non-Toxin | -0.04 | -0.12 | 0.01 | -2.00 | 980.12 |  |  |  |  |  |  |  |  |  |  |  |  |  |  |  |  |  |  |
| [AGAAYLDNDY](https://webs.iiitd.edu.in/raghava/toxinpred/pepsearch_S.php?seq=AGAAYLDNDY&thval=0.0) | -0.97 | Non-Toxin | -0.06 | -0.43 | -0.17 | -2.00 | 1072.22 |  |  |  |  |  |  |  |  |  |  |  |  |  |  |  |  |  |  |
| [GAAYLDNDYD](https://webs.iiitd.edu.in/raghava/toxinpred/pepsearch_S.php?seq=GAAYLDNDYD&thval=0.0) | -0.94 | Non-Toxin | -0.16 | -0.96 | 0.18 | -3.00 | 1116.23 |  |  |  |  |  |  |  |  |  |  |  |  |  |  |  |  |  |  |
| [AAYLDNDYDL](https://webs.iiitd.edu.in/raghava/toxinpred/pepsearch_S.php?seq=AAYLDNDYDL&thval=0.0) | -1.03 | Non-Toxin | -0.12 | -0.54 | 0.00 | -3.00 | 1172.34 |  |  |  |  |  |  |  |  |  |  |  |  |  |  |  |  |  |  |
| [AYLDNDYDLA](https://webs.iiitd.edu.in/raghava/toxinpred/pepsearch_S.php?seq=AYLDNDYDLA&thval=0.0) | -1.04 | Non-Toxin | -0.12 | -0.54 | 0.00 | -3.00 | 1172.34 |  |  |  |  |  |  |  |  |  |  |  |  |  |  |  |  |  |  |
| [YLDNDYDLAK](https://webs.iiitd.edu.in/raghava/toxinpred/pepsearch_S.php?seq=YLDNDYDLAK&thval=0.0) | -0.83 | Non-Toxin | -0.26 | -1.11 | 0.35 | -2.00 | 1229.44 |  |  |  |  |  |  |  |  |  |  |  |  |  |  |  |  |  |  |
| [LDNDYDLAKR](https://webs.iiitd.edu.in/raghava/toxinpred/pepsearch_S.php?seq=LDNDYDLAKR&thval=0.0) | -0.66 | Non-Toxin | -0.43 | -1.43 | 0.88 | -1.00 | 1222.45 |  |  |  |  |  |  |  |  |  |  |  |  |  |  |  |  |  |  |
| [DNDYDLAKRI](https://webs.iiitd.edu.in/raghava/toxinpred/pepsearch_S.php?seq=DNDYDLAKRI&thval=0.0) | -0.72 | Non-Toxin | -0.41 | -1.36 | 0.88 | -1.00 | 1222.45 |  |  |  |  |  |  |  |  |  |  |  |  |  |  |  |  |  |  |
| [NDYDLAKRIG](https://webs.iiitd.edu.in/raghava/toxinpred/pepsearch_S.php?seq=NDYDLAKRIG&thval=0.0) | -0.70 | Non-Toxin | -0.32 | -1.05 | 0.58 | 0.00 | 1164.42 |  |  |  |  |  |  |  |  |  |  |  |  |  |  |  |  |  |  |
| [DYDLAKRIGN](https://webs.iiitd.edu.in/raghava/toxinpred/pepsearch_S.php?seq=DYDLAKRIGN&thval=0.0) | -0.91 | Non-Toxin | -0.33 | -1.05 | 0.58 | 0.00 | 1164.42 |  |  |  |  |  |  |  |  |  |  |  |  |  |  |  |  |  |  |
| [YDLAKRIGNG](https://webs.iiitd.edu.in/raghava/toxinpred/pepsearch_S.php?seq=YDLAKRIGNG&thval=0.0) | -1.00 | Non-Toxin | -0.24 | -0.74 | 0.28 | 1.00 | 1106.39 |  |  |  |  |  |  |  |  |  |  |  |  |  |  |  |  |  |  |
| [DLAKRIGNGG](https://webs.iiitd.edu.in/raghava/toxinpred/pepsearch_S.php?seq=DLAKRIGNGG&thval=0.0) | -1.18 | Non-Toxin | -0.22 | -0.65 | 0.51 | 1.00 | 1000.27 |  |  |  |  |  |  |  |  |  |  |  |  |  |  |  |  |  |  |
| [LAKRIGNGGT](https://webs.iiitd.edu.in/raghava/toxinpred/pepsearch_S.php?seq=LAKRIGNGGT&thval=0.0) | -1.09 | Non-Toxin | -0.17 | -0.37 | 0.17 | 2.00 | 986.29 |  |  |  |  |  |  |  |  |  |  |  |  |  |  |  |  |  |  |
| [AKRIGNGGTL](https://webs.iiitd.edu.in/raghava/toxinpred/pepsearch_S.php?seq=AKRIGNGGTL&thval=0.0) | -1.11 | Non-Toxin | -0.17 | -0.37 | 0.17 | 2.00 | 986.29 |  |  |  |  |  |  |  |  |  |  |  |  |  |  |  |  |  |  |
| [KRIGNGGTLT](https://webs.iiitd.edu.in/raghava/toxinpred/pepsearch_S.php?seq=KRIGNGGTLT&thval=0.0) | -1.37 | Non-Toxin | -0.21 | -0.62 | 0.18 | 2.00 | 1016.32 |  |  |  |  |  |  |  |  |  |  |  |  |  |  |  |  |  |  |
| [RIGNGGTLTI](https://webs.iiitd.edu.in/raghava/toxinpred/pepsearch_S.php?seq=RIGNGGTLTI&thval=0.0) | -1.46 | Non-Toxin | -0.03 | 0.22 | -0.30 | 1.00 | 1001.31 |  |  |  |  |  |  |  |  |  |  |  |  |  |  |  |  |  |  |
| [IGNGGTLTID](https://webs.iiitd.edu.in/raghava/toxinpred/pepsearch_S.php?seq=IGNGGTLTID&thval=0.0) | -1.21 | Non-Toxin | 0.08 | 0.32 | -0.30 | -1.00 | 960.21 |  |  |  |  |  |  |  |  |  |  |  |  |  |  |  |  |  |  |
| [GNGGTLTIDG](https://webs.iiitd.edu.in/raghava/toxinpred/pepsearch_S.php?seq=GNGGTLTIDG&thval=0.0) | -1.08 | Non-Toxin | 0.02 | -0.17 | -0.12 | -1.00 | 904.10 |  |  |  |  |  |  |  |  |  |  |  |  |  |  |  |  |  |  |
| [NGGTLTIDGN](https://webs.iiitd.edu.in/raghava/toxinpred/pepsearch_S.php?seq=NGGTLTIDGN&thval=0.0) | -1.08 | Non-Toxin | -0.06 | -0.48 | -0.10 | -1.00 | 961.15 |  |  |  |  |  |  |  |  |  |  |  |  |  |  |  |  |  |  |
| [GGTLTIDGNT](https://webs.iiitd.edu.in/raghava/toxinpred/pepsearch_S.php?seq=GGTLTIDGNT&thval=0.0) | -1.13 | Non-Toxin | -0.02 | -0.20 | -0.16 | -1.00 | 948.15 |  |  |  |  |  |  |  |  |  |  |  |  |  |  |  |  |  |  |
| [GTLTIDGNTY](https://webs.iiitd.edu.in/raghava/toxinpred/pepsearch_S.php?seq=GTLTIDGNTY&thval=0.0) | -1.01 | Non-Toxin | -0.03 | -0.29 | -0.39 | -1.00 | 1054.27 |  |  |  |  |  |  |  |  |  |  |  |  |  |  |  |  |  |  |
| [TLTIDGNTYQ](https://webs.iiitd.edu.in/raghava/toxinpred/pepsearch_S.php?seq=TLTIDGNTYQ&thval=0.0) | -0.94 | Non-Toxin | -0.11 | -0.60 | -0.37 | -1.00 | 1125.35 |  |  |  |  |  |  |  |  |  |  |  |  |  |  |  |  |  |  |
| [LTIDGNTYQQ](https://webs.iiitd.edu.in/raghava/toxinpred/pepsearch_S.php?seq=LTIDGNTYQQ&thval=0.0) | -0.85 | Non-Toxin | -0.17 | -0.88 | -0.31 | -1.00 | 1152.38 |  |  |  |  |  |  |  |  |  |  |  |  |  |  |  |  |  |  |
| [TIDGNTYQQA](https://webs.iiitd.edu.in/raghava/toxinpred/pepsearch_S.php?seq=TIDGNTYQQA&thval=0.0) | -0.80 | Non-Toxin | -0.19 | -1.08 | -0.18 | -1.00 | 1110.29 |  |  |  |  |  |  |  |  |  |  |  |  |  |  |  |  |  |  |
| [IDGNTYQQAA](https://webs.iiitd.edu.in/raghava/toxinpred/pepsearch_S.php?seq=IDGNTYQQAA&thval=0.0) | -0.93 | Non-Toxin | -0.15 | -0.83 | -0.19 | -1.00 | 1080.26 |  |  |  |  |  |  |  |  |  |  |  |  |  |  |  |  |  |  |
| [DGNTYQQAAA](https://webs.iiitd.edu.in/raghava/toxinpred/pepsearch_S.php?seq=DGNTYQQAAA&thval=0.0) | -1.10 | Non-Toxin | -0.20 | -1.10 | -0.06 | -1.00 | 1038.17 |  |  |  |  |  |  |  |  |  |  |  |  |  |  |  |  |  |  |
| [GNTYQQAAAG](https://webs.iiitd.edu.in/raghava/toxinpred/pepsearch_S.php?seq=GNTYQQAAAG&thval=0.0) | -1.25 | Non-Toxin | -0.11 | -0.79 | -0.36 | 0.00 | 980.14 |  |  |  |  |  |  |  |  |  |  |  |  |  |  |  |  |  |  |
| [NTYQQAAAGQ](https://webs.iiitd.edu.in/raghava/toxinpred/pepsearch_S.php?seq=NTYQQAAAGQ&thval=0.0) | -1.13 | Non-Toxin | -0.20 | -1.10 | -0.34 | 0.00 | 1051.22 |  |  |  |  |  |  |  |  |  |  |  |  |  |  |  |  |  |  |
| [TYQQAAAGQE](https://webs.iiitd.edu.in/raghava/toxinpred/pepsearch_S.php?seq=TYQQAAAGQE&thval=0.0) | -1.13 | Non-Toxin | -0.19 | -1.10 | -0.06 | -1.00 | 1066.23 |  |  |  |  |  |  |  |  |  |  |  |  |  |  |  |  |  |  |
| [YQQAAAGQEG](https://webs.iiitd.edu.in/raghava/toxinpred/pepsearch_S.php?seq=YQQAAAGQEG&thval=0.0) | -1.12 | Non-Toxin | -0.16 | -1.07 | -0.02 | -1.00 | 1022.18 |  |  |  |  |  |  |  |  |  |  |  |  |  |  |  |  |  |  |
| [QQAAAGQEGG](https://webs.iiitd.edu.in/raghava/toxinpred/pepsearch_S.php?seq=QQAAAGQEGG&thval=0.0) | -1.25 | Non-Toxin | -0.15 | -0.98 | 0.21 | -1.00 | 916.06 |  |  |  |  |  |  |  |  |  |  |  |  |  |  |  |  |  |  |
| [QAAAGQEGGV](https://webs.iiitd.edu.in/raghava/toxinpred/pepsearch_S.php?seq=QAAAGQEGGV&thval=0.0) | -1.29 | Non-Toxin | -0.02 | -0.21 | 0.04 | -1.00 | 887.06 |  |  |  |  |  |  |  |  |  |  |  |  |  |  |  |  |  |  |
| [AAAGQEGGVR](https://webs.iiitd.edu.in/raghava/toxinpred/pepsearch_S.php?seq=AAAGQEGGVR&thval=0.0) | -1.22 | Non-Toxin | -0.13 | -0.31 | 0.32 | 0.00 | 915.11 |  |  |  |  |  |  |  |  |  |  |  |  |  |  |  |  |  |  |
|  |  |  |  |  |  |  |  |  |  |  |  |  |  |  |  |  |  |  |  |  |  |  |  |  |  |

| **Peptides Scanned from Original Protein** | | | | |  | | | | | | | | | | | | | | | | | | | | |
| --- | --- | --- | --- | --- | --- | --- | --- | --- | --- | --- | --- | --- | --- | --- | --- | --- | --- | --- | --- | --- | --- | --- | --- | --- | --- |
| [**Peptide Sequence**](https://webs.iiitd.edu.in/raghava/toxinpred/prot_submitfreq_S.php?ran=85160) | [**SVM score**](https://webs.iiitd.edu.in/raghava/toxinpred/prot_submitfreq_S.php?ran=85160) | [**Prediction**](https://webs.iiitd.edu.in/raghava/toxinpred/prot_submitfreq_S.php?ran=85160) | [**Hydrophobicity**](https://webs.iiitd.edu.in/raghava/toxinpred/prot_submitfreq_S.php?ran=85160) | [**Hydropathicity**](https://webs.iiitd.edu.in/raghava/toxinpred/prot_submitfreq_S.php?ran=85160) | [**Hydrophilicity**](https://webs.iiitd.edu.in/raghava/toxinpred/prot_submitfreq_S.php?ran=85160) | [**Charge**](https://webs.iiitd.edu.in/raghava/toxinpred/prot_submitfreq_S.php?ran=85160) | [**Mol wt**](https://webs.iiitd.edu.in/raghava/toxinpred/prot_submitfreq_S.php?ran=85160) |  |  |  |  |  |  |  |  |  |  |  |  |  |  |  |  |  |  |
| [KNYDSKIKPY](https://webs.iiitd.edu.in/raghava/toxinpred/pepsearch_S.php?seq=KNYDSKIKPY&thval=0.0) | -0.64 | Non-Toxin | -0.42 | -1.92 | 0.61 | 2.00 | 1255.57 |  |  |  |  |  |  |  |  |  |  |  |  |  |  |  |  |  |  |
| [NYDSKIKPYV](https://webs.iiitd.edu.in/raghava/toxinpred/pepsearch_S.php?seq=NYDSKIKPYV&thval=0.0) | -0.82 | Non-Toxin | -0.26 | -1.11 | 0.16 | 1.00 | 1226.53 |  |  |  |  |  |  |  |  |  |  |  |  |  |  |  |  |  |  |
| [YDSKIKPYVL](https://webs.iiitd.edu.in/raghava/toxinpred/pepsearch_S.php?seq=YDSKIKPYVL&thval=0.0) | -0.94 | Non-Toxin | -0.14 | -0.38 | -0.04 | 1.00 | 1225.59 |  |  |  |  |  |  |  |  |  |  |  |  |  |  |  |  |  |  |
| [DSKIKPYVLL](https://webs.iiitd.edu.in/raghava/toxinpred/pepsearch_S.php?seq=DSKIKPYVLL&thval=0.0) | -1.17 | Non-Toxin | -0.09 | 0.13 | 0.01 | 1.00 | 1175.58 |  |  |  |  |  |  |  |  |  |  |  |  |  |  |  |  |  |  |
| [SKIKPYVLLG](https://webs.iiitd.edu.in/raghava/toxinpred/pepsearch_S.php?seq=SKIKPYVLLG&thval=0.0) | -1.18 | Non-Toxin | -0.00 | 0.44 | -0.29 | 2.00 | 1117.55 |  |  |  |  |  |  |  |  |  |  |  |  |  |  |  |  |  |  |
| [KIKPYVLLGA](https://webs.iiitd.edu.in/raghava/toxinpred/pepsearch_S.php?seq=KIKPYVLLGA&thval=0.0) | -1.04 | Non-Toxin | 0.05 | 0.70 | -0.37 | 2.00 | 1101.55 |  |  |  |  |  |  |  |  |  |  |  |  |  |  |  |  |  |  |
| [IKPYVLLGAG](https://webs.iiitd.edu.in/raghava/toxinpred/pepsearch_S.php?seq=IKPYVLLGAG&thval=0.0) | -1.05 | Non-Toxin | 0.17 | 1.05 | -0.67 | 1.00 | 1030.43 |  |  |  |  |  |  |  |  |  |  |  |  |  |  |  |  |  |  |
| [KPYVLLGAGH](https://webs.iiitd.edu.in/raghava/toxinpred/pepsearch_S.php?seq=KPYVLLGAGH&thval=0.0) | -1.08 | Non-Toxin | 0.06 | 0.28 | -0.54 | 1.50 | 1054.41 |  |  |  |  |  |  |  |  |  |  |  |  |  |  |  |  |  |  |
| [PYVLLGAGHY](https://webs.iiitd.edu.in/raghava/toxinpred/pepsearch_S.php?seq=PYVLLGAGHY&thval=0.0) | -1.02 | Non-Toxin | 0.17 | 0.54 | -1.07 | 0.50 | 1089.41 |  |  |  |  |  |  |  |  |  |  |  |  |  |  |  |  |  |  |
| [YVLLGAGHYK](https://webs.iiitd.edu.in/raghava/toxinpred/pepsearch_S.php?seq=YVLLGAGHYK&thval=0.0) | -0.90 | Non-Toxin | 0.07 | 0.31 | -0.77 | 1.50 | 1120.47 |  |  |  |  |  |  |  |  |  |  |  |  |  |  |  |  |  |  |
| [VLLGAGHYKY](https://webs.iiitd.edu.in/raghava/toxinpred/pepsearch_S.php?seq=VLLGAGHYKY&thval=0.0) | -0.80 | Non-Toxin | 0.07 | 0.31 | -0.77 | 1.50 | 1120.47 |  |  |  |  |  |  |  |  |  |  |  |  |  |  |  |  |  |  |
| [LLGAGHYKYD](https://webs.iiitd.edu.in/raghava/toxinpred/pepsearch_S.php?seq=LLGAGHYKYD&thval=0.0) | -0.66 | Non-Toxin | -0.06 | -0.46 | -0.32 | 0.50 | 1136.42 |  |  |  |  |  |  |  |  |  |  |  |  |  |  |  |  |  |  |
| [LGAGHYKYDF](https://webs.iiitd.edu.in/raghava/toxinpred/pepsearch_S.php?seq=LGAGHYKYDF&thval=0.0) | -0.82 | Non-Toxin | -0.05 | -0.56 | -0.39 | 0.50 | 1170.43 |  |  |  |  |  |  |  |  |  |  |  |  |  |  |  |  |  |  |
| [GAGHYKYDFD](https://webs.iiitd.edu.in/raghava/toxinpred/pepsearch_S.php?seq=GAGHYKYDFD&thval=0.0) | -0.86 | Non-Toxin | -0.17 | -1.29 | 0.09 | -0.50 | 1172.35 |  |  |  |  |  |  |  |  |  |  |  |  |  |  |  |  |  |  |
| [AGHYKYDFDG](https://webs.iiitd.edu.in/raghava/toxinpred/pepsearch_S.php?seq=AGHYKYDFDG&thval=0.0) | -0.86 | Non-Toxin | -0.17 | -1.29 | 0.09 | -0.50 | 1172.35 |  |  |  |  |  |  |  |  |  |  |  |  |  |  |  |  |  |  |
| [GHYKYDFDGV](https://webs.iiitd.edu.in/raghava/toxinpred/pepsearch_S.php?seq=GHYKYDFDGV&thval=0.0) | -1.07 | Non-Toxin | -0.14 | -1.05 | -0.01 | -0.50 | 1200.41 |  |  |  |  |  |  |  |  |  |  |  |  |  |  |  |  |  |  |
| [HYKYDFDGVN](https://webs.iiitd.edu.in/raghava/toxinpred/pepsearch_S.php?seq=HYKYDFDGVN&thval=0.0) | -1.07 | Non-Toxin | -0.22 | -1.36 | 0.01 | -0.50 | 1257.46 |  |  |  |  |  |  |  |  |  |  |  |  |  |  |  |  |  |  |
| [YKYDFDGVNR](https://webs.iiitd.edu.in/raghava/toxinpred/pepsearch_S.php?seq=YKYDFDGVNR&thval=0.0) | -0.96 | Non-Toxin | -0.36 | -1.49 | 0.36 | 0.00 | 1276.50 |  |  |  |  |  |  |  |  |  |  |  |  |  |  |  |  |  |  |
| [KYDFDGVNRG](https://webs.iiitd.edu.in/raghava/toxinpred/pepsearch_S.php?seq=KYDFDGVNRG&thval=0.0) | -1.17 | Non-Toxin | -0.35 | -1.40 | 0.59 | 0.00 | 1170.38 |  |  |  |  |  |  |  |  |  |  |  |  |  |  |  |  |  |  |
| [YDFDGVNRGT](https://webs.iiitd.edu.in/raghava/toxinpred/pepsearch_S.php?seq=YDFDGVNRGT&thval=0.0) | -1.12 | Non-Toxin | -0.25 | -1.08 | 0.25 | -1.00 | 1143.31 |  |  |  |  |  |  |  |  |  |  |  |  |  |  |  |  |  |  |
| [DFDGVNRGTR](https://webs.iiitd.edu.in/raghava/toxinpred/pepsearch_S.php?seq=DFDGVNRGTR&thval=0.0) | -0.95 | Non-Toxin | -0.43 | -1.40 | 0.78 | 0.00 | 1136.32 |  |  |  |  |  |  |  |  |  |  |  |  |  |  |  |  |  |  |
| [FDGVNRGTRG](https://webs.iiitd.edu.in/raghava/toxinpred/pepsearch_S.php?seq=FDGVNRGTRG&thval=0.0) | -0.80 | Non-Toxin | -0.34 | -1.09 | 0.48 | 1.00 | 1078.29 |  |  |  |  |  |  |  |  |  |  |  |  |  |  |  |  |  |  |
| [DGVNRGTRGT](https://webs.iiitd.edu.in/raghava/toxinpred/pepsearch_S.php?seq=DGVNRGTRGT&thval=0.0) | -0.70 | Non-Toxin | -0.42 | -1.44 | 0.69 | 1.00 | 1032.22 |  |  |  |  |  |  |  |  |  |  |  |  |  |  |  |  |  |  |
| [GVNRGTRGTS](https://webs.iiitd.edu.in/raghava/toxinpred/pepsearch_S.php?seq=GVNRGTRGTS&thval=0.0) | -0.80 | Non-Toxin | -0.38 | -1.17 | 0.42 | 2.00 | 1004.21 |  |  |  |  |  |  |  |  |  |  |  |  |  |  |  |  |  |  |
| [VNRGTRGTSE](https://webs.iiitd.edu.in/raghava/toxinpred/pepsearch_S.php?seq=VNRGTRGTSE&thval=0.0) | -0.64 | Non-Toxin | -0.45 | -1.48 | 0.72 | 1.00 | 1076.27 |  |  |  |  |  |  |  |  |  |  |  |  |  |  |  |  |  |  |
| [NRGTRGTSEE](https://webs.iiitd.edu.in/raghava/toxinpred/pepsearch_S.php?seq=NRGTRGTSEE&thval=0.0) | -0.53 | Non-Toxin | -0.57 | -2.25 | 1.17 | 0.00 | 1106.25 |  |  |  |  |  |  |  |  |  |  |  |  |  |  |  |  |  |  |
| [RGTRGTSEEG](https://webs.iiitd.edu.in/raghava/toxinpred/pepsearch_S.php?seq=RGTRGTSEEG&thval=0.0) | -0.51 | Non-Toxin | -0.49 | -1.94 | 1.15 | 0.00 | 1049.20 |  |  |  |  |  |  |  |  |  |  |  |  |  |  |  |  |  |  |
| [GTRGTSEEGT](https://webs.iiitd.edu.in/raghava/toxinpred/pepsearch_S.php?seq=GTRGTSEEGT&thval=0.0) | -0.57 | Non-Toxin | -0.33 | -1.56 | 0.81 | -1.00 | 994.12 |  |  |  |  |  |  |  |  |  |  |  |  |  |  |  |  |  |  |
| [TRGTSEEGTL](https://webs.iiitd.edu.in/raghava/toxinpred/pepsearch_S.php?seq=TRGTSEEGTL&thval=0.0) | -0.58 | Non-Toxin | -0.30 | -1.14 | 0.63 | -1.00 | 1050.23 |  |  |  |  |  |  |  |  |  |  |  |  |  |  |  |  |  |  |
| [RGTSEEGTLG](https://webs.iiitd.edu.in/raghava/toxinpred/pepsearch_S.php?seq=RGTSEEGTLG&thval=0.0) | -0.84 | Non-Toxin | -0.26 | -1.11 | 0.67 | -1.00 | 1006.18 |  |  |  |  |  |  |  |  |  |  |  |  |  |  |  |  |  |  |
| [GTSEEGTLGN](https://webs.iiitd.edu.in/raghava/toxinpred/pepsearch_S.php?seq=GTSEEGTLGN&thval=0.0) | -0.90 | Non-Toxin | -0.15 | -1.01 | 0.39 | -2.00 | 964.10 |  |  |  |  |  |  |  |  |  |  |  |  |  |  |  |  |  |  |
| [TSEEGTLGNA](https://webs.iiitd.edu.in/raghava/toxinpred/pepsearch_S.php?seq=TSEEGTLGNA&thval=0.0) | -0.63 | Non-Toxin | -0.14 | -0.79 | 0.34 | -2.00 | 978.12 |  |  |  |  |  |  |  |  |  |  |  |  |  |  |  |  |  |  |
| [SEEGTLGNAG](https://webs.iiitd.edu.in/raghava/toxinpred/pepsearch_S.php?seq=SEEGTLGNAG&thval=0.0) | -0.56 | Non-Toxin | -0.11 | -0.76 | 0.38 | -2.00 | 934.07 |  |  |  |  |  |  |  |  |  |  |  |  |  |  |  |  |  |  |
| [EEGTLGNAGV](https://webs.iiitd.edu.in/raghava/toxinpred/pepsearch_S.php?seq=EEGTLGNAGV&thval=0.0) | -0.88 | Non-Toxin | -0.03 | -0.26 | 0.20 | -2.00 | 946.13 |  |  |  |  |  |  |  |  |  |  |  |  |  |  |  |  |  |  |
| [EGTLGNAGVG](https://webs.iiitd.edu.in/raghava/toxinpred/pepsearch_S.php?seq=EGTLGNAGVG&thval=0.0) | -1.06 | Non-Toxin | 0.05 | 0.05 | -0.10 | -1.00 | 874.07 |  |  |  |  |  |  |  |  |  |  |  |  |  |  |  |  |  |  |
| [GTLGNAGVGA](https://webs.iiitd.edu.in/raghava/toxinpred/pepsearch_S.php?seq=GTLGNAGVGA&thval=0.0) | -1.02 | Non-Toxin | 0.14 | 0.58 | -0.45 | 0.00 | 816.03 |  |  |  |  |  |  |  |  |  |  |  |  |  |  |  |  |  |  |
| [TLGNAGVGAF](https://webs.iiitd.edu.in/raghava/toxinpred/pepsearch_S.php?seq=TLGNAGVGAF&thval=0.0) | -1.28 | Non-Toxin | 0.18 | 0.90 | -0.70 | 0.00 | 906.15 |  |  |  |  |  |  |  |  |  |  |  |  |  |  |  |  |  |  |
| [LGNAGVGAFW](https://webs.iiitd.edu.in/raghava/toxinpred/pepsearch_S.php?seq=LGNAGVGAFW&thval=0.0) | -1.32 | Non-Toxin | 0.24 | 0.88 | -1.00 | 0.00 | 991.26 |  |  |  |  |  |  |  |  |  |  |  |  |  |  |  |  |  |  |
| [GNAGVGAFWR](https://webs.iiitd.edu.in/raghava/toxinpred/pepsearch_S.php?seq=GNAGVGAFWR&thval=0.0) | -1.15 | Non-Toxin | 0.01 | 0.05 | -0.52 | 1.00 | 1034.28 |  |  |  |  |  |  |  |  |  |  |  |  |  |  |  |  |  |  |
| [NAGVGAFWRL](https://webs.iiitd.edu.in/raghava/toxinpred/pepsearch_S.php?seq=NAGVGAFWRL&thval=0.0) | -1.04 | Non-Toxin | 0.05 | 0.47 | -0.70 | 1.00 | 1090.39 |  |  |  |  |  |  |  |  |  |  |  |  |  |  |  |  |  |  |
| [AGVGAFWRLN](https://webs.iiitd.edu.in/raghava/toxinpred/pepsearch_S.php?seq=AGVGAFWRLN&thval=0.0) | -1.25 | Non-Toxin | 0.05 | 0.47 | -0.70 | 1.00 | 1090.39 |  |  |  |  |  |  |  |  |  |  |  |  |  |  |  |  |  |  |
| [GVGAFWRLND](https://webs.iiitd.edu.in/raghava/toxinpred/pepsearch_S.php?seq=GVGAFWRLND&thval=0.0) | -1.24 | Non-Toxin | -0.05 | -0.06 | -0.35 | 0.00 | 1134.40 |  |  |  |  |  |  |  |  |  |  |  |  |  |  |  |  |  |  |
| [VGAFWRLNDA](https://webs.iiitd.edu.in/raghava/toxinpred/pepsearch_S.php?seq=VGAFWRLNDA&thval=0.0) | -1.08 | Non-Toxin | -0.04 | 0.16 | -0.40 | 0.00 | 1148.42 |  |  |  |  |  |  |  |  |  |  |  |  |  |  |  |  |  |  |
| [GAFWRLNDAL](https://webs.iiitd.edu.in/raghava/toxinpred/pepsearch_S.php?seq=GAFWRLNDAL&thval=0.0) | -1.00 | Non-Toxin | -0.04 | 0.12 | -0.43 | 0.00 | 1162.45 |  |  |  |  |  |  |  |  |  |  |  |  |  |  |  |  |  |  |
| [AFWRLNDALS](https://webs.iiitd.edu.in/raghava/toxinpred/pepsearch_S.php?seq=AFWRLNDALS&thval=0.0) | -1.02 | Non-Toxin | -0.08 | 0.08 | -0.40 | 0.00 | 1192.47 |  |  |  |  |  |  |  |  |  |  |  |  |  |  |  |  |  |  |
| [FWRLNDALSL](https://webs.iiitd.edu.in/raghava/toxinpred/pepsearch_S.php?seq=FWRLNDALSL&thval=0.0) | -0.96 | Non-Toxin | -0.06 | 0.28 | -0.53 | 0.00 | 1234.56 |  |  |  |  |  |  |  |  |  |  |  |  |  |  |  |  |  |  |
| [WRLNDALSLR](https://webs.iiitd.edu.in/raghava/toxinpred/pepsearch_S.php?seq=WRLNDALSLR&thval=0.0) | -0.95 | Non-Toxin | -0.29 | -0.45 | 0.02 | 1.00 | 1243.57 |  |  |  |  |  |  |  |  |  |  |  |  |  |  |  |  |  |  |
| [RLNDALSLRT](https://webs.iiitd.edu.in/raghava/toxinpred/pepsearch_S.php?seq=RLNDALSLRT&thval=0.0) | -1.04 | Non-Toxin | -0.35 | -0.43 | 0.32 | 1.00 | 1158.46 |  |  |  |  |  |  |  |  |  |  |  |  |  |  |  |  |  |  |
| [LNDALSLRTE](https://webs.iiitd.edu.in/raghava/toxinpred/pepsearch_S.php?seq=LNDALSLRTE&thval=0.0) | -1.07 | Non-Toxin | -0.23 | -0.33 | 0.32 | -1.00 | 1131.39 |  |  |  |  |  |  |  |  |  |  |  |  |  |  |  |  |  |  |
| [NDALSLRTEA](https://webs.iiitd.edu.in/raghava/toxinpred/pepsearch_S.php?seq=NDALSLRTEA&thval=0.0) | -1.11 | Non-Toxin | -0.26 | -0.53 | 0.45 | -1.00 | 1089.30 |  |  |  |  |  |  |  |  |  |  |  |  |  |  |  |  |  |  |
| [DALSLRTEAR](https://webs.iiitd.edu.in/raghava/toxinpred/pepsearch_S.php?seq=DALSLRTEAR&thval=0.0) | -1.26 | Non-Toxin | -0.37 | -0.63 | 0.73 | 0.00 | 1131.38 |  |  |  |  |  |  |  |  |  |  |  |  |  |  |  |  |  |  |
| [ALSLRTEARA](https://webs.iiitd.edu.in/raghava/toxinpred/pepsearch_S.php?seq=ALSLRTEARA&thval=0.0) | -1.28 | Non-Toxin | -0.28 | -0.10 | 0.38 | 1.00 | 1087.37 |  |  |  |  |  |  |  |  |  |  |  |  |  |  |  |  |  |  |
| [LSLRTEARAT](https://webs.iiitd.edu.in/raghava/toxinpred/pepsearch_S.php?seq=LSLRTEARAT&thval=0.0) | -1.32 | Non-Toxin | -0.32 | -0.35 | 0.39 | 1.00 | 1117.40 |  |  |  |  |  |  |  |  |  |  |  |  |  |  |  |  |  |  |
| [SLRTEARATY](https://webs.iiitd.edu.in/raghava/toxinpred/pepsearch_S.php?seq=SLRTEARATY&thval=0.0) | -1.14 | Non-Toxin | -0.37 | -0.86 | 0.34 | 1.00 | 1167.41 |  |  |  |  |  |  |  |  |  |  |  |  |  |  |  |  |  |  |
| [LRTEARATYN](https://webs.iiitd.edu.in/raghava/toxinpred/pepsearch_S.php?seq=LRTEARATYN&thval=0.0) | -1.17 | Non-Toxin | -0.41 | -1.13 | 0.33 | 1.00 | 1194.44 |  |  |  |  |  |  |  |  |  |  |  |  |  |  |  |  |  |  |
| [RTEARATYNA](https://webs.iiitd.edu.in/raghava/toxinpred/pepsearch_S.php?seq=RTEARATYNA&thval=0.0) | -0.85 | Non-Toxin | -0.44 | -1.33 | 0.46 | 1.00 | 1152.35 |  |  |  |  |  |  |  |  |  |  |  |  |  |  |  |  |  |  |
| [TEARATYNAD](https://webs.iiitd.edu.in/raghava/toxinpred/pepsearch_S.php?seq=TEARATYNAD&thval=0.0) | -0.76 | Non-Toxin | -0.33 | -1.23 | 0.46 | -1.00 | 1111.25 |  |  |  |  |  |  |  |  |  |  |  |  |  |  |  |  |  |  |
| [EARATYNADE](https://webs.iiitd.edu.in/raghava/toxinpred/pepsearch_S.php?seq=EARATYNADE&thval=0.0) | -0.63 | Non-Toxin | -0.38 | -1.51 | 0.80 | -2.00 | 1139.26 |  |  |  |  |  |  |  |  |  |  |  |  |  |  |  |  |  |  |
| [ARATYNADEE](https://webs.iiitd.edu.in/raghava/toxinpred/pepsearch_S.php?seq=ARATYNADEE&thval=0.0) | -0.40 | Non-Toxin | -0.38 | -1.51 | 0.80 | -2.00 | 1139.26 |  |  |  |  |  |  |  |  |  |  |  |  |  |  |  |  |  |  |
| [RATYNADEEF](https://webs.iiitd.edu.in/raghava/toxinpred/pepsearch_S.php?seq=RATYNADEEF&thval=0.0) | -0.28 | Non-Toxin | -0.34 | -1.41 | 0.60 | -2.00 | 1215.36 |  |  |  |  |  |  |  |  |  |  |  |  |  |  |  |  |  |  |
| [ATYNADEEFW](https://webs.iiitd.edu.in/raghava/toxinpred/pepsearch_S.php?seq=ATYNADEEFW&thval=0.0) | -0.44 | Non-Toxin | -0.13 | -1.05 | -0.04 | -3.00 | 1245.39 |  |  |  |  |  |  |  |  |  |  |  |  |  |  |  |  |  |  |
| [TYNADEEFWN](https://webs.iiitd.edu.in/raghava/toxinpred/pepsearch_S.php?seq=TYNADEEFWN&thval=0.0) | -0.61 | Non-Toxin | -0.22 | -1.58 | 0.03 | -3.00 | 1288.42 |  |  |  |  |  |  |  |  |  |  |  |  |  |  |  |  |  |  |
| [YNADEEFWNY](https://webs.iiitd.edu.in/raghava/toxinpred/pepsearch_S.php?seq=YNADEEFWNY&thval=0.0) | -0.59 | Non-Toxin | -0.20 | -1.64 | -0.16 | -3.00 | 1350.49 |  |  |  |  |  |  |  |  |  |  |  |  |  |  |  |  |  |  |
| [NADEEFWNYT](https://webs.iiitd.edu.in/raghava/toxinpred/pepsearch_S.php?seq=NADEEFWNYT&thval=0.0) | -0.55 | Non-Toxin | -0.22 | -1.58 | 0.03 | -3.00 | 1288.42 |  |  |  |  |  |  |  |  |  |  |  |  |  |  |  |  |  |  |
| [ADEEFWNYTA](https://webs.iiitd.edu.in/raghava/toxinpred/pepsearch_S.php?seq=ADEEFWNYTA&thval=0.0) | -0.91 | Non-Toxin | -0.13 | -1.05 | -0.04 | -3.00 | 1245.39 |  |  |  |  |  |  |  |  |  |  |  |  |  |  |  |  |  |  |
| [DEEFWNYTAL](https://webs.iiitd.edu.in/raghava/toxinpred/pepsearch_S.php?seq=DEEFWNYTAL&thval=0.0) | -0.94 | Non-Toxin | -0.10 | -0.85 | -0.17 | -3.00 | 1287.48 |  |  |  |  |  |  |  |  |  |  |  |  |  |  |  |  |  |  |
| [EEFWNYTALA](https://webs.iiitd.edu.in/raghava/toxinpred/pepsearch_S.php?seq=EEFWNYTALA&thval=0.0) | -1.15 | Non-Toxin | -0.00 | -0.32 | -0.52 | -2.00 | 1243.47 |  |  |  |  |  |  |  |  |  |  |  |  |  |  |  |  |  |  |
| [EFWNYTALAG](https://webs.iiitd.edu.in/raghava/toxinpred/pepsearch_S.php?seq=EFWNYTALAG&thval=0.0) | -1.24 | Non-Toxin | 0.08 | -0.01 | -0.82 | -1.00 | 1171.41 |  |  |  |  |  |  |  |  |  |  |  |  |  |  |  |  |  |  |
| [FWNYTALAGL](https://webs.iiitd.edu.in/raghava/toxinpred/pepsearch_S.php?seq=FWNYTALAGL&thval=0.0) | -1.24 | Non-Toxin | 0.19 | 0.72 | -1.30 | 0.00 | 1155.46 |  |  |  |  |  |  |  |  |  |  |  |  |  |  |  |  |  |  |
| [WNYTALAGLN](https://webs.iiitd.edu.in/raghava/toxinpred/pepsearch_S.php?seq=WNYTALAGLN&thval=0.0) | -1.24 | Non-Toxin | 0.07 | 0.09 | -1.03 | 0.00 | 1122.39 |  |  |  |  |  |  |  |  |  |  |  |  |  |  |  |  |  |  |
| [NYTALAGLNV](https://webs.iiitd.edu.in/raghava/toxinpred/pepsearch_S.php?seq=NYTALAGLNV&thval=0.0) | -1.11 | Non-Toxin | 0.08 | 0.60 | -0.84 | 0.00 | 1035.31 |  |  |  |  |  |  |  |  |  |  |  |  |  |  |  |  |  |  |

| **Peptides Scanned from Original Protein** | | | | |  | | | | | | | | | | | | | | | | | | | | |
| --- | --- | --- | --- | --- | --- | --- | --- | --- | --- | --- | --- | --- | --- | --- | --- | --- | --- | --- | --- | --- | --- | --- | --- | --- | --- |
| [**Peptide Sequence**](https://webs.iiitd.edu.in/raghava/toxinpred/prot_submitfreq_S.php?ran=81240) | [**SVM score**](https://webs.iiitd.edu.in/raghava/toxinpred/prot_submitfreq_S.php?ran=81240) | [**Prediction**](https://webs.iiitd.edu.in/raghava/toxinpred/prot_submitfreq_S.php?ran=81240) | [**Hydrophobicity**](https://webs.iiitd.edu.in/raghava/toxinpred/prot_submitfreq_S.php?ran=81240) | [**Hydropathicity**](https://webs.iiitd.edu.in/raghava/toxinpred/prot_submitfreq_S.php?ran=81240) | [**Hydrophilicity**](https://webs.iiitd.edu.in/raghava/toxinpred/prot_submitfreq_S.php?ran=81240) | [**Charge**](https://webs.iiitd.edu.in/raghava/toxinpred/prot_submitfreq_S.php?ran=81240) | [**Mol wt**](https://webs.iiitd.edu.in/raghava/toxinpred/prot_submitfreq_S.php?ran=81240) |  |  |  |  |  |  |  |  |  |  |  |  |  |  |  |  |  |  |
| [LRVFFDTNKS](https://webs.iiitd.edu.in/raghava/toxinpred/pepsearch_S.php?seq=LRVFFDTNKS&thval=0.0) | -0.94 | Non-Toxin | -0.24 | -0.33 | 0.08 | 1.00 | 1226.53 |  |  |  |  |  |  |  |  |  |  |  |  |  |  |  |  |  |  |
| [RVFFDTNKSN](https://webs.iiitd.edu.in/raghava/toxinpred/pepsearch_S.php?seq=RVFFDTNKSN&thval=0.0) | -0.91 | Non-Toxin | -0.35 | -1.06 | 0.28 | 1.00 | 1227.47 |  |  |  |  |  |  |  |  |  |  |  |  |  |  |  |  |  |  |
| [VFFDTNKSNI](https://webs.iiitd.edu.in/raghava/toxinpred/pepsearch_S.php?seq=VFFDTNKSNI&thval=0.0) | -0.88 | Non-Toxin | -0.11 | -0.16 | -0.20 | 0.00 | 1184.45 |  |  |  |  |  |  |  |  |  |  |  |  |  |  |  |  |  |  |
| [FFDTNKSNIK](https://webs.iiitd.edu.in/raghava/toxinpred/pepsearch_S.php?seq=FFDTNKSNIK&thval=0.0) | -0.87 | Non-Toxin | -0.27 | -0.97 | 0.25 | 1.00 | 1213.49 |  |  |  |  |  |  |  |  |  |  |  |  |  |  |  |  |  |  |
| [FDTNKSNIKD](https://webs.iiitd.edu.in/raghava/toxinpred/pepsearch_S.php?seq=FDTNKSNIKD&thval=0.0) | -1.00 | Non-Toxin | -0.40 | -1.60 | 0.80 | 0.00 | 1181.40 |  |  |  |  |  |  |  |  |  |  |  |  |  |  |  |  |  |  |
| [DTNKSNIKDQ](https://webs.iiitd.edu.in/raghava/toxinpred/pepsearch_S.php?seq=DTNKSNIKDQ&thval=0.0) | -0.79 | Non-Toxin | -0.53 | -2.23 | 1.07 | 0.00 | 1162.36 |  |  |  |  |  |  |  |  |  |  |  |  |  |  |  |  |  |  |
| [TNKSNIKDQY](https://webs.iiitd.edu.in/raghava/toxinpred/pepsearch_S.php?seq=TNKSNIKDQY&thval=0.0) | -0.85 | Non-Toxin | -0.46 | -2.01 | 0.54 | 1.00 | 1210.45 |  |  |  |  |  |  |  |  |  |  |  |  |  |  |  |  |  |  |
| [NKSNIKDQYK](https://webs.iiitd.edu.in/raghava/toxinpred/pepsearch_S.php?seq=NKSNIKDQYK&thval=0.0) | -0.70 | Non-Toxin | -0.55 | -2.33 | 0.88 | 2.00 | 1237.52 |  |  |  |  |  |  |  |  |  |  |  |  |  |  |  |  |  |  |
| [KSNIKDQYKP](https://webs.iiitd.edu.in/raghava/toxinpred/pepsearch_S.php?seq=KSNIKDQYKP&thval=0.0) | -0.83 | Non-Toxin | -0.49 | -2.14 | 0.86 | 2.00 | 1220.53 |  |  |  |  |  |  |  |  |  |  |  |  |  |  |  |  |  |  |
| [SNIKDQYKPE](https://webs.iiitd.edu.in/raghava/toxinpred/pepsearch_S.php?seq=SNIKDQYKPE&thval=0.0) | -0.88 | Non-Toxin | -0.44 | -2.10 | 0.86 | 0.00 | 1221.47 |  |  |  |  |  |  |  |  |  |  |  |  |  |  |  |  |  |  |
| [NIKDQYKPEI](https://webs.iiitd.edu.in/raghava/toxinpred/pepsearch_S.php?seq=NIKDQYKPEI&thval=0.0) | -0.97 | Non-Toxin | -0.35 | -1.57 | 0.65 | 0.00 | 1247.56 |  |  |  |  |  |  |  |  |  |  |  |  |  |  |  |  |  |  |
| [IKDQYKPEIA](https://webs.iiitd.edu.in/raghava/toxinpred/pepsearch_S.php?seq=IKDQYKPEIA&thval=0.0) | -0.86 | Non-Toxin | -0.26 | -1.04 | 0.58 | 0.00 | 1204.53 |  |  |  |  |  |  |  |  |  |  |  |  |  |  |  |  |  |  |
| [KDQYKPEIAK](https://webs.iiitd.edu.in/raghava/toxinpred/pepsearch_S.php?seq=KDQYKPEIAK&thval=0.0) | -0.60 | Non-Toxin | -0.44 | -1.88 | 1.06 | 1.00 | 1219.54 |  |  |  |  |  |  |  |  |  |  |  |  |  |  |  |  |  |  |
| [DQYKPEIAKV](https://webs.iiitd.edu.in/raghava/toxinpred/pepsearch_S.php?seq=DQYKPEIAKV&thval=0.0) | -0.47 | Non-Toxin | -0.28 | -1.07 | 0.61 | 0.00 | 1190.50 |  |  |  |  |  |  |  |  |  |  |  |  |  |  |  |  |  |  |
| [QYKPEIAKVA](https://webs.iiitd.edu.in/raghava/toxinpred/pepsearch_S.php?seq=QYKPEIAKVA&thval=0.0) | -0.61 | Non-Toxin | -0.18 | -0.54 | 0.26 | 1.00 | 1146.49 |  |  |  |  |  |  |  |  |  |  |  |  |  |  |  |  |  |  |
| [YKPEIAKVAE](https://webs.iiitd.edu.in/raghava/toxinpred/pepsearch_S.php?seq=YKPEIAKVAE&thval=0.0) | -0.48 | Non-Toxin | -0.17 | -0.54 | 0.54 | 0.00 | 1147.47 |  |  |  |  |  |  |  |  |  |  |  |  |  |  |  |  |  |  |
| [KPEIAKVAEK](https://webs.iiitd.edu.in/raghava/toxinpred/pepsearch_S.php?seq=KPEIAKVAEK&thval=0.0) | -0.76 | Non-Toxin | -0.28 | -0.80 | 1.07 | 1.00 | 1112.47 |  |  |  |  |  |  |  |  |  |  |  |  |  |  |  |  |  |  |
| [PEIAKVAEKL](https://webs.iiitd.edu.in/raghava/toxinpred/pepsearch_S.php?seq=PEIAKVAEKL&thval=0.0) | -0.77 | Non-Toxin | -0.12 | -0.03 | 0.59 | 0.00 | 1097.46 |  |  |  |  |  |  |  |  |  |  |  |  |  |  |  |  |  |  |
| [EIAKVAEKLS](https://webs.iiitd.edu.in/raghava/toxinpred/pepsearch_S.php?seq=EIAKVAEKLS&thval=0.0) | -0.83 | Non-Toxin | -0.14 | 0.05 | 0.62 | 0.00 | 1087.42 |  |  |  |  |  |  |  |  |  |  |  |  |  |  |  |  |  |  |
| [IAKVAEKLSE](https://webs.iiitd.edu.in/raghava/toxinpred/pepsearch_S.php?seq=IAKVAEKLSE&thval=0.0) | -0.87 | Non-Toxin | -0.14 | 0.05 | 0.62 | 0.00 | 1087.42 |  |  |  |  |  |  |  |  |  |  |  |  |  |  |  |  |  |  |
| [AKVAEKLSEY](https://webs.iiitd.edu.in/raghava/toxinpred/pepsearch_S.php?seq=AKVAEKLSEY&thval=0.0) | -0.64 | Non-Toxin | -0.21 | -0.53 | 0.57 | 0.00 | 1137.43 |  |  |  |  |  |  |  |  |  |  |  |  |  |  |  |  |  |  |
| [KVAEKLSEYP](https://webs.iiitd.edu.in/raghava/toxinpred/pepsearch_S.php?seq=KVAEKLSEYP&thval=0.0) | -0.72 | Non-Toxin | -0.24 | -0.87 | 0.62 | 0.00 | 1163.47 |  |  |  |  |  |  |  |  |  |  |  |  |  |  |  |  |  |  |
| [VAEKLSEYPN](https://webs.iiitd.edu.in/raghava/toxinpred/pepsearch_S.php?seq=VAEKLSEYPN&thval=0.0) | -0.78 | Non-Toxin | -0.20 | -0.83 | 0.34 | -1.00 | 1149.40 |  |  |  |  |  |  |  |  |  |  |  |  |  |  |  |  |  |  |
| [AEKLSEYPNA](https://webs.iiitd.edu.in/raghava/toxinpred/pepsearch_S.php?seq=AEKLSEYPNA&thval=0.0) | -0.42 | Non-Toxin | -0.23 | -1.07 | 0.44 | -1.00 | 1121.34 |  |  |  |  |  |  |  |  |  |  |  |  |  |  |  |  |  |  |
| [EKLSEYPNAT](https://webs.iiitd.edu.in/raghava/toxinpred/pepsearch_S.php?seq=EKLSEYPNAT&thval=0.0) | -0.41 | Non-Toxin | -0.27 | -1.32 | 0.45 | -1.00 | 1151.37 |  |  |  |  |  |  |  |  |  |  |  |  |  |  |  |  |  |  |
| [KLSEYPNATA](https://webs.iiitd.edu.in/raghava/toxinpred/pepsearch_S.php?seq=KLSEYPNATA&thval=0.0) | -0.25 | Non-Toxin | -0.18 | -0.79 | 0.10 | 0.00 | 1093.33 |  |  |  |  |  |  |  |  |  |  |  |  |  |  |  |  |  |  |
| [LSEYPNATAR](https://webs.iiitd.edu.in/raghava/toxinpred/pepsearch_S.php?seq=LSEYPNATAR&thval=0.0) | -0.39 | Non-Toxin | -0.25 | -0.85 | 0.10 | 0.00 | 1121.34 |  |  |  |  |  |  |  |  |  |  |  |  |  |  |  |  |  |  |
| [SEYPNATARI](https://webs.iiitd.edu.in/raghava/toxinpred/pepsearch_S.php?seq=SEYPNATARI&thval=0.0) | -0.55 | Non-Toxin | -0.23 | -0.78 | 0.10 | 0.00 | 1121.34 |  |  |  |  |  |  |  |  |  |  |  |  |  |  |  |  |  |  |
| [EYPNATARIE](https://webs.iiitd.edu.in/raghava/toxinpred/pepsearch_S.php?seq=EYPNATARIE&thval=0.0) | -0.61 | Non-Toxin | -0.26 | -1.05 | 0.37 | -1.00 | 1163.38 |  |  |  |  |  |  |  |  |  |  |  |  |  |  |  |  |  |  |
| [YPNATARIEG](https://webs.iiitd.edu.in/raghava/toxinpred/pepsearch_S.php?seq=YPNATARIEG&thval=0.0) | -0.56 | Non-Toxin | -0.19 | -0.74 | 0.07 | 0.00 | 1091.32 |  |  |  |  |  |  |  |  |  |  |  |  |  |  |  |  |  |  |
| [PNATARIEGH](https://webs.iiitd.edu.in/raghava/toxinpred/pepsearch_S.php?seq=PNATARIEGH&thval=0.0) | -0.94 | Non-Toxin | -0.23 | -0.93 | 0.25 | 0.50 | 1065.29 |  |  |  |  |  |  |  |  |  |  |  |  |  |  |  |  |  |  |
| [NATARIEGHT](https://webs.iiitd.edu.in/raghava/toxinpred/pepsearch_S.php?seq=NATARIEGHT&thval=0.0) | -0.75 | Non-Toxin | -0.24 | -0.84 | 0.21 | 0.50 | 1069.28 |  |  |  |  |  |  |  |  |  |  |  |  |  |  |  |  |  |  |
| [ATARIEGHTD](https://webs.iiitd.edu.in/raghava/toxinpred/pepsearch_S.php?seq=ATARIEGHTD&thval=0.0) | -0.99 | Non-Toxin | -0.25 | -0.84 | 0.49 | -0.50 | 1070.26 |  |  |  |  |  |  |  |  |  |  |  |  |  |  |  |  |  |  |
| [TARIEGHTDN](https://webs.iiitd.edu.in/raghava/toxinpred/pepsearch_S.php?seq=TARIEGHTDN&thval=0.0) | -1.01 | Non-Toxin | -0.34 | -1.37 | 0.56 | -0.50 | 1113.29 |  |  |  |  |  |  |  |  |  |  |  |  |  |  |  |  |  |  |
| [ARIEGHTDNT](https://webs.iiitd.edu.in/raghava/toxinpred/pepsearch_S.php?seq=ARIEGHTDNT&thval=0.0) | -0.86 | Non-Toxin | -0.34 | -1.37 | 0.56 | -0.50 | 1113.29 |  |  |  |  |  |  |  |  |  |  |  |  |  |  |  |  |  |  |
| [RIEGHTDNTG](https://webs.iiitd.edu.in/raghava/toxinpred/pepsearch_S.php?seq=RIEGHTDNTG&thval=0.0) | -0.72 | Non-Toxin | -0.35 | -1.59 | 0.61 | -0.50 | 1099.27 |  |  |  |  |  |  |  |  |  |  |  |  |  |  |  |  |  |  |
| [IEGHTDNTGP](https://webs.iiitd.edu.in/raghava/toxinpred/pepsearch_S.php?seq=IEGHTDNTGP&thval=0.0) | -0.41 | Non-Toxin | -0.18 | -1.30 | 0.31 | -1.50 | 1040.20 |  |  |  |  |  |  |  |  |  |  |  |  |  |  |  |  |  |  |
| [EGHTDNTGPR](https://webs.iiitd.edu.in/raghava/toxinpred/pepsearch_S.php?seq=EGHTDNTGPR&thval=0.0) | -0.68 | Non-Toxin | -0.42 | -2.20 | 0.79 | -0.50 | 1083.22 |  |  |  |  |  |  |  |  |  |  |  |  |  |  |  |  |  |  |
| [GHTDNTGPRK](https://webs.iiitd.edu.in/raghava/toxinpred/pepsearch_S.php?seq=GHTDNTGPRK&thval=0.0) | -0.68 | Non-Toxin | -0.47 | -2.24 | 0.79 | 1.50 | 1082.28 |  |  |  |  |  |  |  |  |  |  |  |  |  |  |  |  |  |  |
| [HTDNTGPRKL](https://webs.iiitd.edu.in/raghava/toxinpred/pepsearch_S.php?seq=HTDNTGPRKL&thval=0.0) | -0.59 | Non-Toxin | -0.44 | -1.82 | 0.61 | 1.50 | 1138.39 |  |  |  |  |  |  |  |  |  |  |  |  |  |  |  |  |  |  |
| [TDNTGPRKLN](https://webs.iiitd.edu.in/raghava/toxinpred/pepsearch_S.php?seq=TDNTGPRKLN&thval=0.0) | -0.90 | Non-Toxin | -0.46 | -1.85 | 0.68 | 1.00 | 1115.35 |  |  |  |  |  |  |  |  |  |  |  |  |  |  |  |  |  |  |
| [DNTGPRKLNE](https://webs.iiitd.edu.in/raghava/toxinpred/pepsearch_S.php?seq=DNTGPRKLNE&thval=0.0) | -0.87 | Non-Toxin | -0.50 | -2.13 | 1.02 | 0.00 | 1143.36 |  |  |  |  |  |  |  |  |  |  |  |  |  |  |  |  |  |  |
| [NTGPRKLNER](https://webs.iiitd.edu.in/raghava/toxinpred/pepsearch_S.php?seq=NTGPRKLNER&thval=0.0) | -1.30 | Non-Toxin | -0.61 | -2.23 | 1.02 | 2.00 | 1184.46 |  |  |  |  |  |  |  |  |  |  |  |  |  |  |  |  |  |  |
| [TGPRKLNERL](https://webs.iiitd.edu.in/raghava/toxinpred/pepsearch_S.php?seq=TGPRKLNERL&thval=0.0) | -1.38 | Non-Toxin | -0.49 | -1.50 | 0.82 | 2.00 | 1183.52 |  |  |  |  |  |  |  |  |  |  |  |  |  |  |  |  |  |  |
| [GPRKLNERLS](https://webs.iiitd.edu.in/raghava/toxinpred/pepsearch_S.php?seq=GPRKLNERLS&thval=0.0) | -1.41 | Non-Toxin | -0.50 | -1.51 | 0.89 | 2.00 | 1169.49 |  |  |  |  |  |  |  |  |  |  |  |  |  |  |  |  |  |  |
| [PRKLNERLSL](https://webs.iiitd.edu.in/raghava/toxinpred/pepsearch_S.php?seq=PRKLNERLSL&thval=0.0) | -1.57 | Non-Toxin | -0.46 | -1.09 | 0.71 | 2.00 | 1225.60 |  |  |  |  |  |  |  |  |  |  |  |  |  |  |  |  |  |  |
| [RKLNERLSLA](https://webs.iiitd.edu.in/raghava/toxinpred/pepsearch_S.php?seq=RKLNERLSLA&thval=0.0) | -1.40 | Non-Toxin | -0.43 | -0.75 | 0.66 | 2.00 | 1199.56 |  |  |  |  |  |  |  |  |  |  |  |  |  |  |  |  |  |  |
| [KLNERLSLAR](https://webs.iiitd.edu.in/raghava/toxinpred/pepsearch_S.php?seq=KLNERLSLAR&thval=0.0) | -1.56 | Non-Toxin | -0.43 | -0.75 | 0.66 | 2.00 | 1199.56 |  |  |  |  |  |  |  |  |  |  |  |  |  |  |  |  |  |  |
| [LNERLSLARA](https://webs.iiitd.edu.in/raghava/toxinpred/pepsearch_S.php?seq=LNERLSLARA&thval=0.0) | -1.51 | Non-Toxin | -0.30 | -0.18 | 0.31 | 1.00 | 1142.46 |  |  |  |  |  |  |  |  |  |  |  |  |  |  |  |  |  |  |
| [NERLSLARAN](https://webs.iiitd.edu.in/raghava/toxinpred/pepsearch_S.php?seq=NERLSLARAN&thval=0.0) | -1.37 | Non-Toxin | -0.41 | -0.91 | 0.51 | 1.00 | 1143.40 |  |  |  |  |  |  |  |  |  |  |  |  |  |  |  |  |  |  |
| [ERLSLARANS](https://webs.iiitd.edu.in/raghava/toxinpred/pepsearch_S.php?seq=ERLSLARANS&thval=0.0) | -1.23 | Non-Toxin | -0.37 | -0.64 | 0.52 | 1.00 | 1116.37 |  |  |  |  |  |  |  |  |  |  |  |  |  |  |  |  |  |  |
| [RLSLARANSV](https://webs.iiitd.edu.in/raghava/toxinpred/pepsearch_S.php?seq=RLSLARANSV&thval=0.0) | -0.91 | Non-Toxin | -0.26 | 0.13 | 0.07 | 2.00 | 1086.39 |  |  |  |  |  |  |  |  |  |  |  |  |  |  |  |  |  |  |
| [LSLARANSVK](https://webs.iiitd.edu.in/raghava/toxinpred/pepsearch_S.php?seq=LSLARANSVK&thval=0.0) | -0.88 | Non-Toxin | -0.19 | 0.19 | 0.07 | 2.00 | 1058.38 |  |  |  |  |  |  |  |  |  |  |  |  |  |  |  |  |  |  |
| [SLARANSVKS](https://webs.iiitd.edu.in/raghava/toxinpred/pepsearch_S.php?seq=SLARANSVKS&thval=0.0) | -0.73 | Non-Toxin | -0.27 | -0.27 | 0.28 | 2.00 | 1032.29 |  |  |  |  |  |  |  |  |  |  |  |  |  |  |  |  |  |  |
| [LARANSVKSA](https://webs.iiitd.edu.in/raghava/toxinpred/pepsearch_S.php?seq=LARANSVKSA&thval=0.0) | -0.87 | Non-Toxin | -0.22 | -0.01 | 0.20 | 2.00 | 1016.29 |  |  |  |  |  |  |  |  |  |  |  |  |  |  |  |  |  |  |
| [ARANSVKSAL](https://webs.iiitd.edu.in/raghava/toxinpred/pepsearch_S.php?seq=ARANSVKSAL&thval=0.0) | -0.64 | Non-Toxin | -0.22 | -0.01 | 0.20 | 2.00 | 1016.29 |  |  |  |  |  |  |  |  |  |  |  |  |  |  |  |  |  |  |
| [RANSVKSALV](https://webs.iiitd.edu.in/raghava/toxinpred/pepsearch_S.php?seq=RANSVKSALV&thval=0.0) | -0.66 | Non-Toxin | -0.19 | 0.23 | 0.10 | 2.00 | 1044.35 |  |  |  |  |  |  |  |  |  |  |  |  |  |  |  |  |  |  |
| [ANSVKSALVN](https://webs.iiitd.edu.in/raghava/toxinpred/pepsearch_S.php?seq=ANSVKSALVN&thval=0.0) | -0.65 | Non-Toxin | -0.08 | 0.33 | -0.18 | 1.00 | 1002.27 |  |  |  |  |  |  |  |  |  |  |  |  |  |  |  |  |  |  |
| [NSVKSALVNE](https://webs.iiitd.edu.in/raghava/toxinpred/pepsearch_S.php?seq=NSVKSALVNE&thval=0.0) | -0.80 | Non-Toxin | -0.17 | -0.20 | 0.17 | 0.00 | 1060.31 |  |  |  |  |  |  |  |  |  |  |  |  |  |  |  |  |  |  |
| [SVKSALVNEY](https://webs.iiitd.edu.in/raghava/toxinpred/pepsearch_S.php?seq=SVKSALVNEY&thval=0.0) | -0.86 | Non-Toxin | -0.10 | 0.02 | -0.08 | 0.00 | 1109.38 |  |  |  |  |  |  |  |  |  |  |  |  |  |  |  |  |  |  |
| [VKSALVNEYN](https://webs.iiitd.edu.in/raghava/toxinpred/pepsearch_S.php?seq=VKSALVNEYN&thval=0.0) | -0.95 | Non-Toxin | -0.14 | -0.25 | -0.09 | 0.00 | 1136.41 |  |  |  |  |  |  |  |  |  |  |  |  |  |  |  |  |  |  |
| [KSALVNEYNV](https://webs.iiitd.edu.in/raghava/toxinpred/pepsearch_S.php?seq=KSALVNEYNV&thval=0.0) | -0.99 | Non-Toxin | -0.14 | -0.25 | -0.09 | 0.00 | 1136.41 |  |  |  |  |  |  |  |  |  |  |  |  |  |  |  |  |  |  |
| [SALVNEYNVD](https://webs.iiitd.edu.in/raghava/toxinpred/pepsearch_S.php?seq=SALVNEYNVD&thval=0.0) | -0.85 | Non-Toxin | -0.10 | -0.21 | -0.09 | -2.00 | 1123.32 |  |  |  |  |  |  |  |  |  |  |  |  |  |  |  |  |  |  |
| [ALVNEYNVDA](https://webs.iiitd.edu.in/raghava/toxinpred/pepsearch_S.php?seq=ALVNEYNVDA&thval=0.0) | -0.87 | Non-Toxin | -0.05 | 0.05 | -0.17 | -2.00 | 1107.32 |  |  |  |  |  |  |  |  |  |  |  |  |  |  |  |  |  |  |
| [LVNEYNVDAS](https://webs.iiitd.edu.in/raghava/toxinpred/pepsearch_S.php?seq=LVNEYNVDAS&thval=0.0) | -0.87 | Non-Toxin | -0.10 | -0.21 | -0.09 | -2.00 | 1123.32 |  |  |  |  |  |  |  |  |  |  |  |  |  |  |  |  |  |  |
| [VNEYNVDASR](https://webs.iiitd.edu.in/raghava/toxinpred/pepsearch_S.php?seq=VNEYNVDASR&thval=0.0) | -0.70 | Non-Toxin | -0.33 | -1.04 | 0.39 | -1.00 | 1166.34 |  |  |  |  |  |  |  |  |  |  |  |  |  |  |  |  |  |  |
| [NEYNVDASRL](https://webs.iiitd.edu.in/raghava/toxinpred/pepsearch_S.php?seq=NEYNVDASRL&thval=0.0) | -0.55 | Non-Toxin | -0.33 | -1.08 | 0.36 | -1.00 | 1180.37 |  |  |  |  |  |  |  |  |  |  |  |  |  |  |  |  |  |  |
| [EYNVDASRLS](https://webs.iiitd.edu.in/raghava/toxinpred/pepsearch_S.php?seq=EYNVDASRLS&thval=0.0) | -0.56 | Non-Toxin | -0.29 | -0.81 | 0.37 | -1.00 | 1153.34 |  |  |  |  |  |  |  |  |  |  |  |  |  |  |  |  |  |  |
| [YNVDASRLST](https://webs.iiitd.edu.in/raghava/toxinpred/pepsearch_S.php?seq=YNVDASRLST&thval=0.0) | -0.71 | Non-Toxin | -0.25 | -0.53 | 0.03 | 0.00 | 1125.33 |  |  |  |  |  |  |  |  |  |  |  |  |  |  |  |  |  |  |
| [NVDASRLSTQ](https://webs.iiitd.edu.in/raghava/toxinpred/pepsearch_S.php?seq=NVDASRLSTQ&thval=0.0) | -0.95 | Non-Toxin | -0.32 | -0.75 | 0.28 | 0.00 | 1090.29 |  |  |  |  |  |  |  |  |  |  |  |  |  |  |  |  |  |  |
| [VDASRLSTQG](https://webs.iiitd.edu.in/raghava/toxinpred/pepsearch_S.php?seq=VDASRLSTQG&thval=0.0) | -1.06 | Non-Toxin | -0.24 | -0.44 | 0.26 | 0.00 | 1033.24 |  |  |  |  |  |  |  |  |  |  |  |  |  |  |  |  |  |  |
| [DASRLSTQGF](https://webs.iiitd.edu.in/raghava/toxinpred/pepsearch_S.php?seq=DASRLSTQGF&thval=0.0) | -1.13 | Non-Toxin | -0.23 | -0.58 | 0.16 | 0.00 | 1081.28 |  |  |  |  |  |  |  |  |  |  |  |  |  |  |  |  |  |  |
| [ASRLSTQGFA](https://webs.iiitd.edu.in/raghava/toxinpred/pepsearch_S.php?seq=ASRLSTQGFA&thval=0.0) | -1.19 | Non-Toxin | -0.14 | -0.05 | -0.19 | 1.00 | 1037.27 |  |  |  |  |  |  |  |  |  |  |  |  |  |  |  |  |  |  |
| [SRLSTQGFAW](https://webs.iiitd.edu.in/raghava/toxinpred/pepsearch_S.php?seq=SRLSTQGFAW&thval=0.0) | -1.09 | Non-Toxin | -0.12 | -0.32 | -0.48 | 1.00 | 1152.41 |  |  |  |  |  |  |  |  |  |  |  |  |  |  |  |  |  |  |
| [RLSTQGFAWD](https://webs.iiitd.edu.in/raghava/toxinpred/pepsearch_S.php?seq=RLSTQGFAWD&thval=0.0) | -1.09 | Non-Toxin | -0.17 | -0.59 | -0.21 | 0.00 | 1180.42 |  |  |  |  |  |  |  |  |  |  |  |  |  |  |  |  |  |  |
| [LSTQGFAWDQ](https://webs.iiitd.edu.in/raghava/toxinpred/pepsearch_S.php?seq=LSTQGFAWDQ&thval=0.0) | -0.93 | Non-Toxin | -0.06 | -0.49 | -0.49 | -1.00 | 1152.37 |  |  |  |  |  |  |  |  |  |  |  |  |  |  |  |  |  |  |
| [STQGFAWDQP](https://webs.iiitd.edu.in/raghava/toxinpred/pepsearch_S.php?seq=STQGFAWDQP&thval=0.0) | -1.15 | Non-Toxin | -0.12 | -1.03 | -0.31 | -1.00 | 1136.32 |  |  |  |  |  |  |  |  |  |  |  |  |  |  |  |  |  |  |
| [TQGFAWDQPI](https://webs.iiitd.edu.in/raghava/toxinpred/pepsearch_S.php?seq=TQGFAWDQPI&thval=0.0) | -1.23 | Non-Toxin | -0.02 | -0.50 | -0.52 | -1.00 | 1162.41 |  |  |  |  |  |  |  |  |  |  |  |  |  |  |  |  |  |  |
| [QGFAWDQPIA](https://webs.iiitd.edu.in/raghava/toxinpred/pepsearch_S.php?seq=QGFAWDQPIA&thval=0.0) | -1.02 | Non-Toxin | 0.02 | -0.25 | -0.53 | -1.00 | 1132.38 |  |  |  |  |  |  |  |  |  |  |  |  |  |  |  |  |  |  |
| [GFAWDQPIAD](https://webs.iiitd.edu.in/raghava/toxinpred/pepsearch_S.php?seq=GFAWDQPIAD&thval=0.0) | -0.81 | Non-Toxin | 0.02 | -0.25 | -0.25 | -2.00 | 1119.33 |  |  |  |  |  |  |  |  |  |  |  |  |  |  |  |  |  |  |
| [FAWDQPIADN](https://webs.iiitd.edu.in/raghava/toxinpred/pepsearch_S.php?seq=FAWDQPIADN&thval=0.0) | -0.66 | Non-Toxin | -0.06 | -0.56 | -0.23 | -2.00 | 1176.38 |  |  |  |  |  |  |  |  |  |  |  |  |  |  |  |  |  |  |
| [AWDQPIADNK](https://webs.iiitd.edu.in/raghava/toxinpred/pepsearch_S.php?seq=AWDQPIADNK&thval=0.0) | -0.40 | Non-Toxin | -0.23 | -1.23 | 0.32 | -1.00 | 1157.38 |  |  |  |  |  |  |  |  |  |  |  |  |  |  |  |  |  |  |
| [WDQPIADNKT](https://webs.iiitd.edu.in/raghava/toxinpred/pepsearch_S.php?seq=WDQPIADNKT&thval=0.0) | -0.37 | Non-Toxin | -0.28 | -1.48 | 0.33 | -1.00 | 1187.41 |  |  |  |  |  |  |  |  |  |  |  |  |  |  |  |  |  |  |
| [DQPIADNKTK](https://webs.iiitd.edu.in/raghava/toxinpred/pepsearch_S.php?seq=DQPIADNKTK&thval=0.0) | -0.90 | Non-Toxin | -0.42 | -1.78 | 0.97 | 0.00 | 1129.37 |  |  |  |  |  |  |  |  |  |  |  |  |  |  |  |  |  |  |
| [QPIADNKTKE](https://webs.iiitd.edu.in/raghava/toxinpred/pepsearch_S.php?seq=QPIADNKTKE&thval=0.0) | -0.92 | Non-Toxin | -0.41 | -1.78 | 0.97 | 0.00 | 1143.40 |  |  |  |  |  |  |  |  |  |  |  |  |  |  |  |  |  |  |
| [PIADNKTKEG](https://webs.iiitd.edu.in/raghava/toxinpred/pepsearch_S.php?seq=PIADNKTKEG&thval=0.0) | -0.69 | Non-Toxin | -0.33 | -1.47 | 0.95 | 0.00 | 1072.32 |  |  |  |  |  |  |  |  |  |  |  |  |  |  |  |  |  |  |
| [IADNKTKEGR](https://webs.iiitd.edu.in/raghava/toxinpred/pepsearch_S.php?seq=IADNKTKEGR&thval=0.0) | -0.47 | Non-Toxin | -0.50 | -1.76 | 1.25 | 1.00 | 1131.39 |  |  |  |  |  |  |  |  |  |  |  |  |  |  |  |  |  |  |
| [ADNKTKEGRA](https://webs.iiitd.edu.in/raghava/toxinpred/pepsearch_S.php?seq=ADNKTKEGRA&thval=0.0) | -0.38 | Non-Toxin | -0.55 | -2.03 | 1.38 | 1.00 | 1089.30 |  |  |  |  |  |  |  |  |  |  |  |  |  |  |  |  |  |  |
| [DNKTKEGRAM](https://webs.iiitd.edu.in/raghava/toxinpred/pepsearch_S.php?seq=DNKTKEGRAM&thval=0.0) | -0.46 | Non-Toxin | -0.55 | -2.02 | 1.30 | 1.00 | 1149.42 |  |  |  |  |  |  |  |  |  |  |  |  |  |  |  |  |  |  |
| [NKTKEGRAMN](https://webs.iiitd.edu.in/raghava/toxinpred/pepsearch_S.php?seq=NKTKEGRAMN&thval=0.0) | -0.53 | Non-Toxin | -0.54 | -2.02 | 1.02 | 2.00 | 1148.44 |  |  |  |  |  |  |  |  |  |  |  |  |  |  |  |  |  |  |
| [KTKEGRAMNR](https://webs.iiitd.edu.in/raghava/toxinpred/pepsearch_S.php?seq=KTKEGRAMNR&thval=0.0) | -0.68 | Non-Toxin | -0.65 | -2.12 | 1.30 | 3.00 | 1190.52 |  |  |  |  |  |  |  |  |  |  |  |  |  |  |  |  |  |  |
| [TKEGRAMNRR](https://webs.iiitd.edu.in/raghava/toxinpred/pepsearch_S.php?seq=TKEGRAMNRR&thval=0.0) | -0.86 | Non-Toxin | -0.72 | -2.18 | 1.30 | 3.00 | 1218.53 |  |  |  |  |  |  |  |  |  |  |  |  |  |  |  |  |  |  |
| [KEGRAMNRRV](https://webs.iiitd.edu.in/raghava/toxinpred/pepsearch_S.php?seq=KEGRAMNRRV&thval=0.0) | -0.80 | Non-Toxin | -0.64 | -1.69 | 1.19 | 3.00 | 1216.56 |  |  |  |  |  |  |  |  |  |  |  |  |  |  |  |  |  |  |
| [EGRAMNRRVF](https://webs.iiitd.edu.in/raghava/toxinpred/pepsearch_S.php?seq=EGRAMNRRVF&thval=0.0) | -0.81 | Non-Toxin | -0.47 | -1.02 | 0.64 | 2.00 | 1235.56 |  |  |  |  |  |  |  |  |  |  |  |  |  |  |  |  |  |  |
| [GRAMNRRVFA](https://webs.iiitd.edu.in/raghava/toxinpred/pepsearch_S.php?seq=GRAMNRRVFA&thval=0.0) | -0.88 | Non-Toxin | -0.38 | -0.49 | 0.29 | 3.00 | 1177.52 |  |  |  |  |  |  |  |  |  |  |  |  |  |  |  |  |  |  |
| [RAMNRRVFAT](https://webs.iiitd.edu.in/raghava/toxinpred/pepsearch_S.php?seq=RAMNRRVFAT&thval=0.0) | -0.94 | Non-Toxin | -0.42 | -0.52 | 0.25 | 3.00 | 1221.57 |  |  |  |  |  |  |  |  |  |  |  |  |  |  |  |  |  |  |
| [AMNRRVFATI](https://webs.iiitd.edu.in/raghava/toxinpred/pepsearch_S.php?seq=AMNRRVFATI&thval=0.0) | -1.02 | Non-Toxin | -0.17 | 0.38 | -0.23 | 2.00 | 1178.55 |  |  |  |  |  |  |  |  |  |  |  |  |  |  |  |  |  |  |
| [MNRRVFATIT](https://webs.iiitd.edu.in/raghava/toxinpred/pepsearch_S.php?seq=MNRRVFATIT&thval=0.0) | -1.28 | Non-Toxin | -0.21 | 0.13 | -0.22 | 2.00 | 1208.58 |  |  |  |  |  |  |  |  |  |  |  |  |  |  |  |  |  |  |
| [NRRVFATITG](https://webs.iiitd.edu.in/raghava/toxinpred/pepsearch_S.php?seq=NRRVFATITG&thval=0.0) | -1.29 | Non-Toxin | -0.22 | -0.10 | -0.09 | 2.00 | 1134.44 |  |  |  |  |  |  |  |  |  |  |  |  |  |  |  |  |  |  |
| [RRVFATITGS](https://webs.iiitd.edu.in/raghava/toxinpred/pepsearch_S.php?seq=RRVFATITGS&thval=0.0) | -1.32 | Non-Toxin | -0.19 | 0.17 | -0.08 | 2.00 | 1107.41 |  |  |  |  |  |  |  |  |  |  |  |  |  |  |  |  |  |  |
| [RVFATITGSR](https://webs.iiitd.edu.in/raghava/toxinpred/pepsearch_S.php?seq=RVFATITGSR&thval=0.0) | -1.13 | Non-Toxin | -0.18 | 0.17 | -0.08 | 2.00 | 1107.41 |  |  |  |  |  |  |  |  |  |  |  |  |  |  |  |  |  |  |
| [VFATITGSRT](https://webs.iiitd.edu.in/raghava/toxinpred/pepsearch_S.php?seq=VFATITGSRT&thval=0.0) | -1.03 | Non-Toxin | -0.03 | 0.55 | -0.42 | 1.00 | 1052.33 |  |  |  |  |  |  |  |  |  |  |  |  |  |  |  |  |  |  |
| [FATITGSRTV](https://webs.iiitd.edu.in/raghava/toxinpred/pepsearch_S.php?seq=FATITGSRTV&thval=0.0) | -1.08 | Non-Toxin | -0.03 | 0.55 | -0.42 | 1.00 | 1052.33 |  |  |  |  |  |  |  |  |  |  |  |  |  |  |  |  |  |  |
| [ATITGSRTVV](https://webs.iiitd.edu.in/raghava/toxinpred/pepsearch_S.php?seq=ATITGSRTVV&thval=0.0) | -1.05 | Non-Toxin | -0.03 | 0.69 | -0.32 | 1.00 | 1004.29 |  |  |  |  |  |  |  |  |  |  |  |  |  |  |  |  |  |  |
| [TITGSRTVVV](https://webs.iiitd.edu.in/raghava/toxinpred/pepsearch_S.php?seq=TITGSRTVVV&thval=0.0) | -1.06 | Non-Toxin | -0.00 | 0.93 | -0.42 | 1.00 | 1032.35 |  |  |  |  |  |  |  |  |  |  |  |  |  |  |  |  |  |  |
| [ITGSRTVVVQ](https://webs.iiitd.edu.in/raghava/toxinpred/pepsearch_S.php?seq=ITGSRTVVVQ&thval=0.0) | -1.09 | Non-Toxin | -0.06 | 0.65 | -0.36 | 1.00 | 1059.38 |  |  |  |  |  |  |  |  |  |  |  |  |  |  |  |  |  |  |
| [TGSRTVVVQP](https://webs.iiitd.edu.in/raghava/toxinpred/pepsearch_S.php?seq=TGSRTVVVQP&thval=0.0) | -1.09 | Non-Toxin | -0.14 | 0.04 | -0.18 | 1.00 | 1043.33 |  |  |  |  |  |  |  |  |  |  |  |  |  |  |  |  |  |  |
| [GSRTVVVQPG](https://webs.iiitd.edu.in/raghava/toxinpred/pepsearch_S.php?seq=GSRTVVVQPG&thval=0.0) | -1.28 | Non-Toxin | -0.10 | 0.07 | -0.14 | 1.00 | 999.28 |  |  |  |  |  |  |  |  |  |  |  |  |  |  |  |  |  |  |
| [SRTVVVQPGQ](https://webs.iiitd.edu.in/raghava/toxinpred/pepsearch_S.php?seq=SRTVVVQPGQ&thval=0.0) | -1.18 | Non-Toxin | -0.19 | -0.24 | -0.12 | 1.00 | 1070.36 |  |  |  |  |  |  |  |  |  |  |  |  |  |  |  |  |  |  |
| [RTVVVQPGQE](https://webs.iiitd.edu.in/raghava/toxinpred/pepsearch_S.php?seq=RTVVVQPGQE&thval=0.0) | -1.22 | Non-Toxin | -0.22 | -0.51 | 0.15 | 0.00 | 1112.40 |  |  |  |  |  |  |  |  |  |  |  |  |  |  |  |  |  |  |
| [TVVVQPGQEA](https://webs.iiitd.edu.in/raghava/toxinpred/pepsearch_S.php?seq=TVVVQPGQEA&thval=0.0) | -1.23 | Non-Toxin | -0.02 | 0.12 | -0.20 | -1.00 | 1027.29 |  |  |  |  |  |  |  |  |  |  |  |  |  |  |  |  |  |  |
| [VVVQPGQEAA](https://webs.iiitd.edu.in/raghava/toxinpred/pepsearch_S.php?seq=VVVQPGQEAA&thval=0.0) | -1.29 | Non-Toxin | 0.02 | 0.37 | -0.21 | -1.00 | 997.26 |  |  |  |  |  |  |  |  |  |  |  |  |  |  |  |  |  |  |

| **Peptides Scanned from Original Protein** | | | | |  | | | | | | | | | | | | | | | | | | | | |
| --- | --- | --- | --- | --- | --- | --- | --- | --- | --- | --- | --- | --- | --- | --- | --- | --- | --- | --- | --- | --- | --- | --- | --- | --- | --- |
| [**Peptide Sequence**](https://webs.iiitd.edu.in/raghava/toxinpred/prot_submitfreq_S.php?ran=30051) | [**SVM score**](https://webs.iiitd.edu.in/raghava/toxinpred/prot_submitfreq_S.php?ran=30051) | [**Prediction**](https://webs.iiitd.edu.in/raghava/toxinpred/prot_submitfreq_S.php?ran=30051) | [**Hydrophobicity**](https://webs.iiitd.edu.in/raghava/toxinpred/prot_submitfreq_S.php?ran=30051) | [**Hydropathicity**](https://webs.iiitd.edu.in/raghava/toxinpred/prot_submitfreq_S.php?ran=30051) | [**Hydrophilicity**](https://webs.iiitd.edu.in/raghava/toxinpred/prot_submitfreq_S.php?ran=30051) | [**Charge**](https://webs.iiitd.edu.in/raghava/toxinpred/prot_submitfreq_S.php?ran=30051) | [**Mol wt**](https://webs.iiitd.edu.in/raghava/toxinpred/prot_submitfreq_S.php?ran=30051) |  |  |  |  |  |  |  |  |  |  |  |  |  |  |  |  |  |  |
| [IITEPAWWGG](https://webs.iiitd.edu.in/raghava/toxinpred/pepsearch_S.php?seq=IITEPAWWGG&thval=0.0) | -1.24 | Non-Toxin | 0.19 | 0.24 | -0.83 | -1.00 | 1129.43 |  |  |  |  |  |  |  |  |  |  |  |  |  |  |  |  |  |  |
| [ITEPAWWGGN](https://webs.iiitd.edu.in/raghava/toxinpred/pepsearch_S.php?seq=ITEPAWWGGN&thval=0.0) | -1.51 | Non-Toxin | 0.05 | -0.56 | -0.63 | -1.00 | 1130.37 |  |  |  |  |  |  |  |  |  |  |  |  |  |  |  |  |  |  |
| [TEPAWWGGNP](https://webs.iiitd.edu.in/raghava/toxinpred/pepsearch_S.php?seq=TEPAWWGGNP&thval=0.0) | -1.00 | Non-Toxin | -0.03 | -1.17 | -0.45 | -1.00 | 1114.32 |  |  |  |  |  |  |  |  |  |  |  |  |  |  |  |  |  |  |
| [EPAWWGGNPD](https://webs.iiitd.edu.in/raghava/toxinpred/pepsearch_S.php?seq=EPAWWGGNPD&thval=0.0) | -0.91 | Non-Toxin | -0.08 | -1.45 | -0.11 | -2.00 | 1128.30 |  |  |  |  |  |  |  |  |  |  |  |  |  |  |  |  |  |  |
| [PAWWGGNPDL](https://webs.iiitd.edu.in/raghava/toxinpred/pepsearch_S.php?seq=PAWWGGNPDL&thval=0.0) | -0.91 | Non-Toxin | 0.03 | -0.72 | -0.59 | -1.00 | 1112.35 |  |  |  |  |  |  |  |  |  |  |  |  |  |  |  |  |  |  |
| [AWWGGNPDLK](https://webs.iiitd.edu.in/raghava/toxinpred/pepsearch_S.php?seq=AWWGGNPDLK&thval=0.0) | -1.04 | Non-Toxin | -0.07 | -0.95 | -0.29 | 0.00 | 1143.41 |  |  |  |  |  |  |  |  |  |  |  |  |  |  |  |  |  |  |
| [WWGGNPDLKP](https://webs.iiitd.edu.in/raghava/toxinpred/pepsearch_S.php?seq=WWGGNPDLKP&thval=0.0) | -1.04 | Non-Toxin | -0.10 | -1.29 | -0.24 | 0.00 | 1169.45 |  |  |  |  |  |  |  |  |  |  |  |  |  |  |  |  |  |  |
| [WGGNPDLKPE](https://webs.iiitd.edu.in/raghava/toxinpred/pepsearch_S.php?seq=WGGNPDLKPE&thval=0.0) | -1.05 | Non-Toxin | -0.20 | -1.55 | 0.40 | -1.00 | 1112.35 |  |  |  |  |  |  |  |  |  |  |  |  |  |  |  |  |  |  |
| [GGNPDLKPEE](https://webs.iiitd.edu.in/raghava/toxinpred/pepsearch_S.php?seq=GGNPDLKPEE&thval=0.0) | -0.83 | Non-Toxin | -0.30 | -1.81 | 1.04 | -2.00 | 1055.25 |  |  |  |  |  |  |  |  |  |  |  |  |  |  |  |  |  |  |
| [GNPDLKPEES](https://webs.iiitd.edu.in/raghava/toxinpred/pepsearch_S.php?seq=GNPDLKPEES&thval=0.0) | -0.87 | Non-Toxin | -0.34 | -1.85 | 1.07 | -2.00 | 1085.27 |  |  |  |  |  |  |  |  |  |  |  |  |  |  |  |  |  |  |
| [NPDLKPEESV](https://webs.iiitd.edu.in/raghava/toxinpred/pepsearch_S.php?seq=NPDLKPEESV&thval=0.0) | -0.70 | Non-Toxin | -0.30 | -1.39 | 0.92 | -2.00 | 1127.35 |  |  |  |  |  |  |  |  |  |  |  |  |  |  |  |  |  |  |
| [PDLKPEESVS](https://webs.iiitd.edu.in/raghava/toxinpred/pepsearch_S.php?seq=PDLKPEESVS&thval=0.0) | -1.11 | Non-Toxin | -0.27 | -1.12 | 0.93 | -2.00 | 1100.32 |  |  |  |  |  |  |  |  |  |  |  |  |  |  |  |  |  |  |
| [DLKPEESVSY](https://webs.iiitd.edu.in/raghava/toxinpred/pepsearch_S.php?seq=DLKPEESVSY&thval=0.0) | -1.20 | Non-Toxin | -0.26 | -1.09 | 0.70 | -2.00 | 1166.38 |  |  |  |  |  |  |  |  |  |  |  |  |  |  |  |  |  |  |
| [LKPEESVSYE](https://webs.iiitd.edu.in/raghava/toxinpred/pepsearch_S.php?seq=LKPEESVSYE&thval=0.0) | -1.14 | Non-Toxin | -0.25 | -1.09 | 0.70 | -2.00 | 1180.41 |  |  |  |  |  |  |  |  |  |  |  |  |  |  |  |  |  |  |
| [KPEESVSYEL](https://webs.iiitd.edu.in/raghava/toxinpred/pepsearch_S.php?seq=KPEESVSYEL&thval=0.0) | -1.01 | Non-Toxin | -0.25 | -1.09 | 0.70 | -2.00 | 1180.41 |  |  |  |  |  |  |  |  |  |  |  |  |  |  |  |  |  |  |
| [PEESVSYELG](https://webs.iiitd.edu.in/raghava/toxinpred/pepsearch_S.php?seq=PEESVSYELG&thval=0.0) | -1.02 | Non-Toxin | -0.12 | -0.74 | 0.40 | -3.00 | 1109.29 |  |  |  |  |  |  |  |  |  |  |  |  |  |  |  |  |  |  |
| [EESVSYELGF](https://webs.iiitd.edu.in/raghava/toxinpred/pepsearch_S.php?seq=EESVSYELGF&thval=0.0) | -1.03 | Non-Toxin | -0.05 | -0.30 | 0.15 | -3.00 | 1159.35 |  |  |  |  |  |  |  |  |  |  |  |  |  |  |  |  |  |  |
| [ESVSYELGFD](https://webs.iiitd.edu.in/raghava/toxinpred/pepsearch_S.php?seq=ESVSYELGFD&thval=0.0) | -1.36 | Non-Toxin | -0.06 | -0.30 | 0.15 | -3.00 | 1145.32 |  |  |  |  |  |  |  |  |  |  |  |  |  |  |  |  |  |  |
| [SVSYELGFDQ](https://webs.iiitd.edu.in/raghava/toxinpred/pepsearch_S.php?seq=SVSYELGFDQ&thval=0.0) | -1.23 | Non-Toxin | -0.07 | -0.30 | -0.13 | -2.00 | 1144.34 |  |  |  |  |  |  |  |  |  |  |  |  |  |  |  |  |  |  |
| [VSYELGFDQK](https://webs.iiitd.edu.in/raghava/toxinpred/pepsearch_S.php?seq=VSYELGFDQK&thval=0.0) | -1.20 | Non-Toxin | -0.15 | -0.61 | 0.14 | -1.00 | 1185.44 |  |  |  |  |  |  |  |  |  |  |  |  |  |  |  |  |  |  |
| [SYELGFDQKL](https://webs.iiitd.edu.in/raghava/toxinpred/pepsearch_S.php?seq=SYELGFDQKL&thval=0.0) | -1.07 | Non-Toxin | -0.15 | -0.65 | 0.11 | -1.00 | 1199.47 |  |  |  |  |  |  |  |  |  |  |  |  |  |  |  |  |  |  |
| [YELGFDQKLN](https://webs.iiitd.edu.in/raghava/toxinpred/pepsearch_S.php?seq=YELGFDQKLN&thval=0.0) | -1.14 | Non-Toxin | -0.19 | -0.92 | 0.10 | -1.00 | 1226.50 |  |  |  |  |  |  |  |  |  |  |  |  |  |  |  |  |  |  |
| [ELGFDQKLNH](https://webs.iiitd.edu.in/raghava/toxinpred/pepsearch_S.php?seq=ELGFDQKLNH&thval=0.0) | -1.01 | Non-Toxin | -0.23 | -1.11 | 0.28 | -0.50 | 1200.47 |  |  |  |  |  |  |  |  |  |  |  |  |  |  |  |  |  |  |
| [LGFDQKLNHG](https://webs.iiitd.edu.in/raghava/toxinpred/pepsearch_S.php?seq=LGFDQKLNHG&thval=0.0) | -0.99 | Non-Toxin | -0.16 | -0.80 | -0.02 | 0.50 | 1128.41 |  |  |  |  |  |  |  |  |  |  |  |  |  |  |  |  |  |  |
| [GFDQKLNHGF](https://webs.iiitd.edu.in/raghava/toxinpred/pepsearch_S.php?seq=GFDQKLNHGF&thval=0.0) | -0.99 | Non-Toxin | -0.15 | -0.90 | -0.09 | 0.50 | 1162.42 |  |  |  |  |  |  |  |  |  |  |  |  |  |  |  |  |  |  |
| [FDQKLNHGFN](https://webs.iiitd.edu.in/raghava/toxinpred/pepsearch_S.php?seq=FDQKLNHGFN&thval=0.0) | -0.98 | Non-Toxin | -0.23 | -1.21 | -0.07 | 0.50 | 1219.47 |  |  |  |  |  |  |  |  |  |  |  |  |  |  |  |  |  |  |
| [DQKLNHGFNV](https://webs.iiitd.edu.in/raghava/toxinpred/pepsearch_S.php?seq=DQKLNHGFNV&thval=0.0) | -0.96 | Non-Toxin | -0.23 | -1.07 | 0.03 | 0.50 | 1171.43 |  |  |  |  |  |  |  |  |  |  |  |  |  |  |  |  |  |  |
| [QKLNHGFNVY](https://webs.iiitd.edu.in/raghava/toxinpred/pepsearch_S.php?seq=QKLNHGFNVY&thval=0.0) | -1.20 | Non-Toxin | -0.16 | -0.85 | -0.50 | 1.50 | 1219.52 |  |  |  |  |  |  |  |  |  |  |  |  |  |  |  |  |  |  |
| [KLNHGFNVYG](https://webs.iiitd.edu.in/raghava/toxinpred/pepsearch_S.php?seq=KLNHGFNVYG&thval=0.0) | -1.18 | Non-Toxin | -0.08 | -0.54 | -0.52 | 1.50 | 1148.44 |  |  |  |  |  |  |  |  |  |  |  |  |  |  |  |  |  |  |
| [LNHGFNVYGS](https://webs.iiitd.edu.in/raghava/toxinpred/pepsearch_S.php?seq=LNHGFNVYGS&thval=0.0) | -0.98 | Non-Toxin | 0.01 | -0.23 | -0.79 | 0.50 | 1107.34 |  |  |  |  |  |  |  |  |  |  |  |  |  |  |  |  |  |  |
| [NHGFNVYGSV](https://webs.iiitd.edu.in/raghava/toxinpred/pepsearch_S.php?seq=NHGFNVYGSV&thval=0.0) | -0.80 | Non-Toxin | 0.01 | -0.19 | -0.76 | 0.50 | 1093.31 |  |  |  |  |  |  |  |  |  |  |  |  |  |  |  |  |  |  |
| [HGFNVYGSVY](https://webs.iiitd.edu.in/raghava/toxinpred/pepsearch_S.php?seq=HGFNVYGSVY&thval=0.0) | -1.05 | Non-Toxin | 0.08 | 0.03 | -1.01 | 0.50 | 1142.38 |  |  |  |  |  |  |  |  |  |  |  |  |  |  |  |  |  |  |
| [GFNVYGSVYQ](https://webs.iiitd.edu.in/raghava/toxinpred/pepsearch_S.php?seq=GFNVYGSVYQ&thval=0.0) | -0.87 | Non-Toxin | 0.05 | 0.00 | -0.94 | 0.00 | 1133.37 |  |  |  |  |  |  |  |  |  |  |  |  |  |  |  |  |  |  |
| [FNVYGSVYQT](https://webs.iiitd.edu.in/raghava/toxinpred/pepsearch_S.php?seq=FNVYGSVYQT&thval=0.0) | -0.83 | Non-Toxin | 0.01 | -0.03 | -0.98 | 0.00 | 1177.42 |  |  |  |  |  |  |  |  |  |  |  |  |  |  |  |  |  |  |
| [NVYGSVYQTK](https://webs.iiitd.edu.in/raghava/toxinpred/pepsearch_S.php?seq=NVYGSVYQTK&thval=0.0) | -1.04 | Non-Toxin | -0.16 | -0.70 | -0.43 | 1.00 | 1158.42 |  |  |  |  |  |  |  |  |  |  |  |  |  |  |  |  |  |  |
| [VYGSVYQTKV](https://webs.iiitd.edu.in/raghava/toxinpred/pepsearch_S.php?seq=VYGSVYQTKV&thval=0.0) | -1.09 | Non-Toxin | -0.04 | 0.07 | -0.60 | 1.00 | 1143.45 |  |  |  |  |  |  |  |  |  |  |  |  |  |  |  |  |  |  |
| [YGSVYQTKVD](https://webs.iiitd.edu.in/raghava/toxinpred/pepsearch_S.php?seq=YGSVYQTKVD&thval=0.0) | -0.93 | Non-Toxin | -0.17 | -0.70 | -0.15 | 0.00 | 1159.40 |  |  |  |  |  |  |  |  |  |  |  |  |  |  |  |  |  |  |
| [GSVYQTKVDN](https://webs.iiitd.edu.in/raghava/toxinpred/pepsearch_S.php?seq=GSVYQTKVDN&thval=0.0) | -1.04 | Non-Toxin | -0.23 | -0.92 | 0.10 | 0.00 | 1110.33 |  |  |  |  |  |  |  |  |  |  |  |  |  |  |  |  |  |  |
| [SVYQTKVDNL](https://webs.iiitd.edu.in/raghava/toxinpred/pepsearch_S.php?seq=SVYQTKVDNL&thval=0.0) | -0.93 | Non-Toxin | -0.20 | -0.50 | -0.08 | 0.00 | 1166.44 |  |  |  |  |  |  |  |  |  |  |  |  |  |  |  |  |  |  |
| [VYQTKVDNLM](https://webs.iiitd.edu.in/raghava/toxinpred/pepsearch_S.php?seq=VYQTKVDNLM&thval=0.0) | -1.06 | Non-Toxin | -0.14 | -0.23 | -0.24 | 0.00 | 1210.56 |  |  |  |  |  |  |  |  |  |  |  |  |  |  |  |  |  |  |
| [YQTKVDNLMV](https://webs.iiitd.edu.in/raghava/toxinpred/pepsearch_S.php?seq=YQTKVDNLMV&thval=0.0) | -0.86 | Non-Toxin | -0.14 | -0.23 | -0.24 | 0.00 | 1210.56 |  |  |  |  |  |  |  |  |  |  |  |  |  |  |  |  |  |  |
| [QTKVDNLMVS](https://webs.iiitd.edu.in/raghava/toxinpred/pepsearch_S.php?seq=QTKVDNLMVS&thval=0.0) | -1.11 | Non-Toxin | -0.17 | -0.18 | 0.02 | 0.00 | 1134.46 |  |  |  |  |  |  |  |  |  |  |  |  |  |  |  |  |  |  |
| [TKVDNLMVSS](https://webs.iiitd.edu.in/raghava/toxinpred/pepsearch_S.php?seq=TKVDNLMVSS&thval=0.0) | -0.88 | Non-Toxin | -0.13 | 0.09 | 0.03 | 0.00 | 1093.40 |  |  |  |  |  |  |  |  |  |  |  |  |  |  |  |  |  |  |
| [KVDNLMVSSA](https://webs.iiitd.edu.in/raghava/toxinpred/pepsearch_S.php?seq=KVDNLMVSSA&thval=0.0) | -0.56 | Non-Toxin | -0.09 | 0.34 | 0.02 | 0.00 | 1063.37 |  |  |  |  |  |  |  |  |  |  |  |  |  |  |  |  |  |  |
| [VDNLMVSSAA](https://webs.iiitd.edu.in/raghava/toxinpred/pepsearch_S.php?seq=VDNLMVSSAA&thval=0.0) | -0.65 | Non-Toxin | 0.05 | 0.91 | -0.33 | -1.00 | 1006.27 |  |  |  |  |  |  |  |  |  |  |  |  |  |  |  |  |  |  |
| [DNLMVSSAAT](https://webs.iiitd.edu.in/raghava/toxinpred/pepsearch_S.php?seq=DNLMVSSAAT&thval=0.0) | -0.76 | Non-Toxin | -0.02 | 0.42 | -0.22 | -1.00 | 1008.24 |  |  |  |  |  |  |  |  |  |  |  |  |  |  |  |  |  |  |
| [NLMVSSAATN](https://webs.iiitd.edu.in/raghava/toxinpred/pepsearch_S.php?seq=NLMVSSAATN&thval=0.0) | -0.78 | Non-Toxin | -0.01 | 0.42 | -0.50 | 0.00 | 1007.26 |  |  |  |  |  |  |  |  |  |  |  |  |  |  |  |  |  |  |
| [LMVSSAATNF](https://webs.iiitd.edu.in/raghava/toxinpred/pepsearch_S.php?seq=LMVSSAATNF&thval=0.0) | -1.10 | Non-Toxin | 0.11 | 1.05 | -0.77 | 0.00 | 1040.33 |  |  |  |  |  |  |  |  |  |  |  |  |  |  |  |  |  |  |
| [MVSSAATNFV](https://webs.iiitd.edu.in/raghava/toxinpred/pepsearch_S.php?seq=MVSSAATNFV&thval=0.0) | -1.09 | Non-Toxin | 0.11 | 1.09 | -0.74 | 0.00 | 1026.30 |  |  |  |  |  |  |  |  |  |  |  |  |  |  |  |  |  |  |
| [VSSAATNFVF](https://webs.iiitd.edu.in/raghava/toxinpred/pepsearch_S.php?seq=VSSAATNFVF&thval=0.0) | -1.14 | Non-Toxin | 0.15 | 1.18 | -0.86 | 0.00 | 1042.28 |  |  |  |  |  |  |  |  |  |  |  |  |  |  |  |  |  |  |
| [SSAATNFVFY](https://webs.iiitd.edu.in/raghava/toxinpred/pepsearch_S.php?seq=SSAATNFVFY&thval=0.0) | -1.13 | Non-Toxin | 0.09 | 0.63 | -0.94 | 0.00 | 1106.32 |  |  |  |  |  |  |  |  |  |  |  |  |  |  |  |  |  |  |
| [SAATNFVFYN](https://webs.iiitd.edu.in/raghava/toxinpred/pepsearch_S.php?seq=SAATNFVFYN&thval=0.0) | -1.45 | Non-Toxin | 0.06 | 0.36 | -0.95 | 0.00 | 1133.35 |  |  |  |  |  |  |  |  |  |  |  |  |  |  |  |  |  |  |
| [AATNFVFYNI](https://webs.iiitd.edu.in/raghava/toxinpred/pepsearch_S.php?seq=AATNFVFYNI&thval=0.0) | -1.49 | Non-Toxin | 0.15 | 0.89 | -1.16 | 0.00 | 1159.44 |  |  |  |  |  |  |  |  |  |  |  |  |  |  |  |  |  |  |
| [ATNFVFYNID](https://webs.iiitd.edu.in/raghava/toxinpred/pepsearch_S.php?seq=ATNFVFYNID&thval=0.0) | -1.42 | Non-Toxin | 0.06 | 0.36 | -0.81 | -1.00 | 1203.45 |  |  |  |  |  |  |  |  |  |  |  |  |  |  |  |  |  |  |
| [TNFVFYNIDK](https://webs.iiitd.edu.in/raghava/toxinpred/pepsearch_S.php?seq=TNFVFYNIDK&thval=0.0) | -1.37 | Non-Toxin | -0.08 | -0.21 | -0.46 | 0.00 | 1260.55 |  |  |  |  |  |  |  |  |  |  |  |  |  |  |  |  |  |  |
| [NFVFYNIDKA](https://webs.iiitd.edu.in/raghava/toxinpred/pepsearch_S.php?seq=NFVFYNIDKA&thval=0.0) | -1.33 | Non-Toxin | -0.03 | 0.04 | -0.47 | 0.00 | 1230.52 |  |  |  |  |  |  |  |  |  |  |  |  |  |  |  |  |  |  |
| [FVFYNIDKAT](https://webs.iiitd.edu.in/raghava/toxinpred/pepsearch_S.php?seq=FVFYNIDKAT&thval=0.0) | -1.11 | Non-Toxin | 0.01 | 0.32 | -0.53 | 0.00 | 1217.52 |  |  |  |  |  |  |  |  |  |  |  |  |  |  |  |  |  |  |
| [VFYNIDKATL](https://webs.iiitd.edu.in/raghava/toxinpred/pepsearch_S.php?seq=VFYNIDKATL&thval=0.0) | -1.17 | Non-Toxin | 0.00 | 0.42 | -0.46 | 0.00 | 1183.51 |  |  |  |  |  |  |  |  |  |  |  |  |  |  |  |  |  |  |
| [FYNIDKATLT](https://webs.iiitd.edu.in/raghava/toxinpred/pepsearch_S.php?seq=FYNIDKATLT&thval=0.0) | -1.27 | Non-Toxin | -0.07 | -0.07 | -0.35 | 0.00 | 1185.48 |  |  |  |  |  |  |  |  |  |  |  |  |  |  |  |  |  |  |
| [YNIDKATLTG](https://webs.iiitd.edu.in/raghava/toxinpred/pepsearch_S.php?seq=YNIDKATLTG&thval=0.0) | -1.19 | Non-Toxin | -0.11 | -0.39 | -0.10 | 0.00 | 1095.36 |  |  |  |  |  |  |  |  |  |  |  |  |  |  |  |  |  |  |
| [NIDKATLTGA](https://webs.iiitd.edu.in/raghava/toxinpred/pepsearch_S.php?seq=NIDKATLTGA&thval=0.0) | -0.96 | Non-Toxin | -0.09 | -0.08 | 0.08 | 0.00 | 1003.26 |  |  |  |  |  |  |  |  |  |  |  |  |  |  |  |  |  |  |
| [IDKATLTGAE](https://webs.iiitd.edu.in/raghava/toxinpred/pepsearch_S.php?seq=IDKATLTGAE&thval=0.0) | -0.80 | Non-Toxin | -0.09 | -0.08 | 0.36 | -1.00 | 1018.27 |  |  |  |  |  |  |  |  |  |  |  |  |  |  |  |  |  |  |
| [DKATLTGAEL](https://webs.iiitd.edu.in/raghava/toxinpred/pepsearch_S.php?seq=DKATLTGAEL&thval=0.0) | -0.92 | Non-Toxin | -0.11 | -0.15 | 0.36 | -1.00 | 1018.27 |  |  |  |  |  |  |  |  |  |  |  |  |  |  |  |  |  |  |
| [KATLTGAELG](https://webs.iiitd.edu.in/raghava/toxinpred/pepsearch_S.php?seq=KATLTGAELG&thval=0.0) | -1.12 | Non-Toxin | -0.02 | 0.16 | 0.06 | 0.00 | 960.24 |  |  |  |  |  |  |  |  |  |  |  |  |  |  |  |  |  |  |
| [ATLTGAELGL](https://webs.iiitd.edu.in/raghava/toxinpred/pepsearch_S.php?seq=ATLTGAELGL&thval=0.0) | -1.06 | Non-Toxin | 0.14 | 0.93 | -0.42 | -1.00 | 945.23 |  |  |  |  |  |  |  |  |  |  |  |  |  |  |  |  |  |  |
| [TLTGAELGLK](https://webs.iiitd.edu.in/raghava/toxinpred/pepsearch_S.php?seq=TLTGAELGLK&thval=0.0) | -1.26 | Non-Toxin | 0.01 | 0.36 | -0.07 | 0.00 | 1002.33 |  |  |  |  |  |  |  |  |  |  |  |  |  |  |  |  |  |  |
| [LTGAELGLKW](https://webs.iiitd.edu.in/raghava/toxinpred/pepsearch_S.php?seq=LTGAELGLKW&thval=0.0) | -0.94 | Non-Toxin | 0.06 | 0.34 | -0.37 | 0.00 | 1087.44 |  |  |  |  |  |  |  |  |  |  |  |  |  |  |  |  |  |  |
| [TGAELGLKWS](https://webs.iiitd.edu.in/raghava/toxinpred/pepsearch_S.php?seq=TGAELGLKWS&thval=0.0) | -1.00 | Non-Toxin | -0.02 | -0.12 | -0.16 | 0.00 | 1061.35 |  |  |  |  |  |  |  |  |  |  |  |  |  |  |  |  |  |  |
| [GAELGLKWSL](https://webs.iiitd.edu.in/raghava/toxinpred/pepsearch_S.php?seq=GAELGLKWSL&thval=0.0) | -0.99 | Non-Toxin | 0.06 | 0.33 | -0.30 | 0.00 | 1073.41 |  |  |  |  |  |  |  |  |  |  |  |  |  |  |  |  |  |  |
| [AELGLKWSLD](https://webs.iiitd.edu.in/raghava/toxinpred/pepsearch_S.php?seq=AELGLKWSLD&thval=0.0) | -1.20 | Non-Toxin | -0.03 | 0.02 | 0.00 | -1.00 | 1131.44 |  |  |  |  |  |  |  |  |  |  |  |  |  |  |  |  |  |  |
| [ELGLKWSLDN](https://webs.iiitd.edu.in/raghava/toxinpred/pepsearch_S.php?seq=ELGLKWSLDN&thval=0.0) | -1.26 | Non-Toxin | -0.12 | -0.51 | 0.07 | -1.00 | 1174.47 |  |  |  |  |  |  |  |  |  |  |  |  |  |  |  |  |  |  |
| [LGLKWSLDNW](https://webs.iiitd.edu.in/raghava/toxinpred/pepsearch_S.php?seq=LGLKWSLDNW&thval=0.0) | -1.03 | Non-Toxin | -0.02 | -0.25 | -0.57 | 0.00 | 1231.57 |  |  |  |  |  |  |  |  |  |  |  |  |  |  |  |  |  |  |

| **Peptides Scanned from Original Protein** | | | | |  | | | | | | | | | | | | | | | | | | | | |
| --- | --- | --- | --- | --- | --- | --- | --- | --- | --- | --- | --- | --- | --- | --- | --- | --- | --- | --- | --- | --- | --- | --- | --- | --- | --- |
| [**Peptide Sequence**](https://webs.iiitd.edu.in/raghava/toxinpred/prot_submitfreq_S.php?ran=76630) | [**SVM score**](https://webs.iiitd.edu.in/raghava/toxinpred/prot_submitfreq_S.php?ran=76630) | [**Prediction**](https://webs.iiitd.edu.in/raghava/toxinpred/prot_submitfreq_S.php?ran=76630) | [**Hydrophobicity**](https://webs.iiitd.edu.in/raghava/toxinpred/prot_submitfreq_S.php?ran=76630) | [**Hydropathicity**](https://webs.iiitd.edu.in/raghava/toxinpred/prot_submitfreq_S.php?ran=76630) | [**Hydrophilicity**](https://webs.iiitd.edu.in/raghava/toxinpred/prot_submitfreq_S.php?ran=76630) | [**Charge**](https://webs.iiitd.edu.in/raghava/toxinpred/prot_submitfreq_S.php?ran=76630) | [**Mol wt**](https://webs.iiitd.edu.in/raghava/toxinpred/prot_submitfreq_S.php?ran=76630) |  |  |  |  |  |  |  |  |  |  |  |  |  |  |  |  |  |  |
| [TATVAGQNIH](https://webs.iiitd.edu.in/raghava/toxinpred/pepsearch_S.php?seq=TATVAGQNIH&thval=0.0) | -1.24 | Non-Toxin | -0.02 | 0.03 | -0.52 | 0.50 | 1011.25 |  |  |  |  |  |  |  |  |  |  |  |  |  |  |  |  |  |  |
| [ATVAGQNIHL](https://webs.iiitd.edu.in/raghava/toxinpred/pepsearch_S.php?seq=ATVAGQNIHL&thval=0.0) | -1.01 | Non-Toxin | 0.06 | 0.48 | -0.66 | 0.50 | 1023.31 |  |  |  |  |  |  |  |  |  |  |  |  |  |  |  |  |  |  |
| [TVAGQNIHLF](https://webs.iiitd.edu.in/raghava/toxinpred/pepsearch_S.php?seq=TVAGQNIHLF&thval=0.0) | -0.99 | Non-Toxin | 0.09 | 0.58 | -0.86 | 0.50 | 1099.41 |  |  |  |  |  |  |  |  |  |  |  |  |  |  |  |  |  |  |
| [VAGQNIHLFD](https://webs.iiitd.edu.in/raghava/toxinpred/pepsearch_S.php?seq=VAGQNIHLFD&thval=0.0) | -1.02 | Non-Toxin | 0.04 | 0.30 | -0.52 | -0.50 | 1113.39 |  |  |  |  |  |  |  |  |  |  |  |  |  |  |  |  |  |  |
| [AGQNIHLFDT](https://webs.iiitd.edu.in/raghava/toxinpred/pepsearch_S.php?seq=AGQNIHLFDT&thval=0.0) | -0.88 | Non-Toxin | -0.03 | -0.19 | -0.41 | -0.50 | 1115.36 |  |  |  |  |  |  |  |  |  |  |  |  |  |  |  |  |  |  |
| [GQNIHLFDTT](https://webs.iiitd.edu.in/raghava/toxinpred/pepsearch_S.php?seq=GQNIHLFDTT&thval=0.0) | -0.81 | Non-Toxin | -0.08 | -0.44 | -0.40 | -0.50 | 1145.39 |  |  |  |  |  |  |  |  |  |  |  |  |  |  |  |  |  |  |
| [QNIHLFDTTD](https://webs.iiitd.edu.in/raghava/toxinpred/pepsearch_S.php?seq=QNIHLFDTTD&thval=0.0) | -0.84 | Non-Toxin | -0.17 | -0.75 | -0.10 | -1.50 | 1203.42 |  |  |  |  |  |  |  |  |  |  |  |  |  |  |  |  |  |  |
| [NIHLFDTTDI](https://webs.iiitd.edu.in/raghava/toxinpred/pepsearch_S.php?seq=NIHLFDTTDI&thval=0.0) | -0.90 | Non-Toxin | -0.02 | 0.05 | -0.30 | -1.50 | 1188.45 |  |  |  |  |  |  |  |  |  |  |  |  |  |  |  |  |  |  |
| [IHLFDTTDIK](https://webs.iiitd.edu.in/raghava/toxinpred/pepsearch_S.php?seq=IHLFDTTDIK&thval=0.0) | -0.86 | Non-Toxin | -0.07 | 0.01 | -0.02 | -0.50 | 1202.52 |  |  |  |  |  |  |  |  |  |  |  |  |  |  |  |  |  |  |
| [HLFDTTDIKQ](https://webs.iiitd.edu.in/raghava/toxinpred/pepsearch_S.php?seq=HLFDTTDIKQ&thval=0.0) | -0.70 | Non-Toxin | -0.21 | -0.79 | 0.18 | -0.50 | 1217.49 |  |  |  |  |  |  |  |  |  |  |  |  |  |  |  |  |  |  |
| [LFDTTDIKQI](https://webs.iiitd.edu.in/raghava/toxinpred/pepsearch_S.php?seq=LFDTTDIKQI&thval=0.0) | -0.71 | Non-Toxin | -0.10 | -0.02 | 0.05 | -1.00 | 1193.51 |  |  |  |  |  |  |  |  |  |  |  |  |  |  |  |  |  |  |
| [FDTTDIKQIE](https://webs.iiitd.edu.in/raghava/toxinpred/pepsearch_S.php?seq=FDTTDIKQIE&thval=0.0) | -0.74 | Non-Toxin | -0.21 | -0.75 | 0.53 | -2.00 | 1209.46 |  |  |  |  |  |  |  |  |  |  |  |  |  |  |  |  |  |  |
| [DTTDIKQIEI](https://webs.iiitd.edu.in/raghava/toxinpred/pepsearch_S.php?seq=DTTDIKQIEI&thval=0.0) | -0.74 | Non-Toxin | -0.20 | -0.58 | 0.60 | -2.00 | 1175.45 |  |  |  |  |  |  |  |  |  |  |  |  |  |  |  |  |  |  |
| [TTDIKQIEIL](https://webs.iiitd.edu.in/raghava/toxinpred/pepsearch_S.php?seq=TTDIKQIEIL&thval=0.0) | -0.81 | Non-Toxin | -0.08 | 0.15 | 0.12 | -1.00 | 1173.53 |  |  |  |  |  |  |  |  |  |  |  |  |  |  |  |  |  |  |
| [TDIKQIEILR](https://webs.iiitd.edu.in/raghava/toxinpred/pepsearch_S.php?seq=TDIKQIEILR&thval=0.0) | -0.96 | Non-Toxin | -0.24 | -0.23 | 0.46 | 0.00 | 1228.61 |  |  |  |  |  |  |  |  |  |  |  |  |  |  |  |  |  |  |
| [DIKQIEILRG](https://webs.iiitd.edu.in/raghava/toxinpred/pepsearch_S.php?seq=DIKQIEILRG&thval=0.0) | -1.04 | Non-Toxin | -0.20 | -0.20 | 0.50 | 0.00 | 1184.56 |  |  |  |  |  |  |  |  |  |  |  |  |  |  |  |  |  |  |
| [IKQIEILRGP](https://webs.iiitd.edu.in/raghava/toxinpred/pepsearch_S.php?seq=IKQIEILRGP&thval=0.0) | -0.84 | Non-Toxin | -0.14 | -0.01 | 0.20 | 1.00 | 1166.59 |  |  |  |  |  |  |  |  |  |  |  |  |  |  |  |  |  |  |
| [KQIEILRGPA](https://webs.iiitd.edu.in/raghava/toxinpred/pepsearch_S.php?seq=KQIEILRGPA&thval=0.0) | -0.78 | Non-Toxin | -0.18 | -0.28 | 0.33 | 1.00 | 1124.50 |  |  |  |  |  |  |  |  |  |  |  |  |  |  |  |  |  |  |
| [QIEILRGPAS](https://webs.iiitd.edu.in/raghava/toxinpred/pepsearch_S.php?seq=QIEILRGPAS&thval=0.0) | -0.93 | Non-Toxin | -0.10 | 0.03 | 0.06 | 0.00 | 1083.40 |  |  |  |  |  |  |  |  |  |  |  |  |  |  |  |  |  |  |
| [IEILRGPASV](https://webs.iiitd.edu.in/raghava/toxinpred/pepsearch_S.php?seq=IEILRGPASV&thval=0.0) | -0.87 | Non-Toxin | 0.02 | 0.80 | -0.11 | 0.00 | 1054.40 |  |  |  |  |  |  |  |  |  |  |  |  |  |  |  |  |  |  |
| [EILRGPASVQ](https://webs.iiitd.edu.in/raghava/toxinpred/pepsearch_S.php?seq=EILRGPASVQ&thval=0.0) | -0.84 | Non-Toxin | -0.12 | 0.00 | 0.09 | 0.00 | 1069.37 |  |  |  |  |  |  |  |  |  |  |  |  |  |  |  |  |  |  |
| [ILRGPASVQY](https://webs.iiitd.edu.in/raghava/toxinpred/pepsearch_S.php?seq=ILRGPASVQY&thval=0.0) | -0.92 | Non-Toxin | -0.05 | 0.22 | -0.44 | 1.00 | 1103.43 |  |  |  |  |  |  |  |  |  |  |  |  |  |  |  |  |  |  |
| [LRGPASVQYG](https://webs.iiitd.edu.in/raghava/toxinpred/pepsearch_S.php?seq=LRGPASVQYG&thval=0.0) | -0.93 | Non-Toxin | -0.11 | -0.27 | -0.26 | 1.00 | 1047.32 |  |  |  |  |  |  |  |  |  |  |  |  |  |  |  |  |  |  |
| [RGPASVQYGT](https://webs.iiitd.edu.in/raghava/toxinpred/pepsearch_S.php?seq=RGPASVQYGT&thval=0.0) | -0.81 | Non-Toxin | -0.18 | -0.72 | -0.12 | 1.00 | 1035.26 |  |  |  |  |  |  |  |  |  |  |  |  |  |  |  |  |  |  |
| [GPASVQYGTD](https://webs.iiitd.edu.in/raghava/toxinpred/pepsearch_S.php?seq=GPASVQYGTD&thval=0.0) | -0.78 | Non-Toxin | -0.08 | -0.62 | -0.12 | -1.00 | 994.16 |  |  |  |  |  |  |  |  |  |  |  |  |  |  |  |  |  |  |
| [PASVQYGTDA](https://webs.iiitd.edu.in/raghava/toxinpred/pepsearch_S.php?seq=PASVQYGTDA&thval=0.0) | -0.81 | Non-Toxin | -0.07 | -0.40 | -0.17 | -1.00 | 1008.18 |  |  |  |  |  |  |  |  |  |  |  |  |  |  |  |  |  |  |
| [ASVQYGTDAI](https://webs.iiitd.edu.in/raghava/toxinpred/pepsearch_S.php?seq=ASVQYGTDAI&thval=0.0) | -0.82 | Non-Toxin | 0.01 | 0.21 | -0.35 | -1.00 | 1024.23 |  |  |  |  |  |  |  |  |  |  |  |  |  |  |  |  |  |  |
| [SVQYGTDAIG](https://webs.iiitd.edu.in/raghava/toxinpred/pepsearch_S.php?seq=SVQYGTDAIG&thval=0.0) | -0.72 | Non-Toxin | 0.00 | -0.01 | -0.30 | -1.00 | 1010.21 |  |  |  |  |  |  |  |  |  |  |  |  |  |  |  |  |  |  |
| [VQYGTDAIGG](https://webs.iiitd.edu.in/raghava/toxinpred/pepsearch_S.php?seq=VQYGTDAIGG&thval=0.0) | -0.86 | Non-Toxin | 0.04 | 0.03 | -0.33 | -1.00 | 980.19 |  |  |  |  |  |  |  |  |  |  |  |  |  |  |  |  |  |  |
| [QYGTDAIGGV](https://webs.iiitd.edu.in/raghava/toxinpred/pepsearch_S.php?seq=QYGTDAIGGV&thval=0.0) | -1.02 | Non-Toxin | 0.04 | 0.03 | -0.33 | -1.00 | 980.19 |  |  |  |  |  |  |  |  |  |  |  |  |  |  |  |  |  |  |
| [YGTDAIGGVI](https://webs.iiitd.edu.in/raghava/toxinpred/pepsearch_S.php?seq=YGTDAIGGVI&thval=0.0) | -0.90 | Non-Toxin | 0.18 | 0.83 | -0.53 | -1.00 | 965.22 |  |  |  |  |  |  |  |  |  |  |  |  |  |  |  |  |  |  |
| [GTDAIGGVIQ](https://webs.iiitd.edu.in/raghava/toxinpred/pepsearch_S.php?seq=GTDAIGGVIQ&thval=0.0) | -1.15 | Non-Toxin | 0.11 | 0.61 | -0.28 | -1.00 | 930.18 |  |  |  |  |  |  |  |  |  |  |  |  |  |  |  |  |  |  |
| [TDAIGGVIQL](https://webs.iiitd.edu.in/raghava/toxinpred/pepsearch_S.php?seq=TDAIGGVIQL&thval=0.0) | -1.38 | Non-Toxin | 0.15 | 1.03 | -0.46 | -1.00 | 986.29 |  |  |  |  |  |  |  |  |  |  |  |  |  |  |  |  |  |  |
| [DAIGGVIQLI](https://webs.iiitd.edu.in/raghava/toxinpred/pepsearch_S.php?seq=DAIGGVIQLI&thval=0.0) | -1.27 | Non-Toxin | 0.24 | 1.55 | -0.60 | -1.00 | 998.35 |  |  |  |  |  |  |  |  |  |  |  |  |  |  |  |  |  |  |
| [AIGGVIQLIS](https://webs.iiitd.edu.in/raghava/toxinpred/pepsearch_S.php?seq=AIGGVIQLIS&thval=0.0) | -1.42 | Non-Toxin | 0.29 | 1.82 | -0.87 | 0.00 | 970.34 |  |  |  |  |  |  |  |  |  |  |  |  |  |  |  |  |  |  |
| [IGGVIQLISK](https://webs.iiitd.edu.in/raghava/toxinpred/pepsearch_S.php?seq=IGGVIQLISK&thval=0.0) | -1.50 | Non-Toxin | 0.15 | 1.25 | -0.52 | 1.00 | 1027.44 |  |  |  |  |  |  |  |  |  |  |  |  |  |  |  |  |  |  |
| [GGVIQLISKT](https://webs.iiitd.edu.in/raghava/toxinpred/pepsearch_S.php?seq=GGVIQLISKT&thval=0.0) | -1.48 | Non-Toxin | 0.06 | 0.73 | -0.38 | 1.00 | 1015.38 |  |  |  |  |  |  |  |  |  |  |  |  |  |  |  |  |  |  |
| [GVIQLISKTP](https://webs.iiitd.edu.in/raghava/toxinpred/pepsearch_S.php?seq=GVIQLISKTP&thval=0.0) | -1.49 | Non-Toxin | 0.04 | 0.61 | -0.38 | 1.00 | 1055.44 |  |  |  |  |  |  |  |  |  |  |  |  |  |  |  |  |  |  |
| [VIQLISKTPT](https://webs.iiitd.edu.in/raghava/toxinpred/pepsearch_S.php?seq=VIQLISKTPT&thval=0.0) | -1.41 | Non-Toxin | 0.00 | 0.58 | -0.42 | 1.00 | 1099.49 |  |  |  |  |  |  |  |  |  |  |  |  |  |  |  |  |  |  |
| [IQLISKTPTQ](https://webs.iiitd.edu.in/raghava/toxinpred/pepsearch_S.php?seq=IQLISKTPTQ&thval=0.0) | -1.68 | Non-Toxin | -0.12 | -0.19 | -0.25 | 1.00 | 1128.49 |  |  |  |  |  |  |  |  |  |  |  |  |  |  |  |  |  |  |
| [QLISKTPTQN](https://webs.iiitd.edu.in/raghava/toxinpred/pepsearch_S.php?seq=QLISKTPTQN&thval=0.0) | -1.52 | Non-Toxin | -0.26 | -0.99 | -0.05 | 1.00 | 1129.43 |  |  |  |  |  |  |  |  |  |  |  |  |  |  |  |  |  |  |
| [LISKTPTQNK](https://webs.iiitd.edu.in/raghava/toxinpred/pepsearch_S.php?seq=LISKTPTQNK&thval=0.0) | -1.21 | Non-Toxin | -0.30 | -1.03 | 0.23 | 2.00 | 1129.47 |  |  |  |  |  |  |  |  |  |  |  |  |  |  |  |  |  |  |
| [ISKTPTQNKI](https://webs.iiitd.edu.in/raghava/toxinpred/pepsearch_S.php?seq=ISKTPTQNKI&thval=0.0) | -1.20 | Non-Toxin | -0.28 | -0.96 | 0.23 | 2.00 | 1129.47 |  |  |  |  |  |  |  |  |  |  |  |  |  |  |  |  |  |  |
| [SKTPTQNKIF](https://webs.iiitd.edu.in/raghava/toxinpred/pepsearch_S.php?seq=SKTPTQNKIF&thval=0.0) | -1.22 | Non-Toxin | -0.29 | -1.13 | 0.16 | 2.00 | 1163.48 |  |  |  |  |  |  |  |  |  |  |  |  |  |  |  |  |  |  |
| [KTPTQNKIFT](https://webs.iiitd.edu.in/raghava/toxinpred/pepsearch_S.php?seq=KTPTQNKIFT&thval=0.0) | -1.26 | Non-Toxin | -0.28 | -1.12 | 0.09 | 2.00 | 1177.51 |  |  |  |  |  |  |  |  |  |  |  |  |  |  |  |  |  |  |
| [TPTQNKIFTT](https://webs.iiitd.edu.in/raghava/toxinpred/pepsearch_S.php?seq=TPTQNKIFTT&thval=0.0) | -1.27 | Non-Toxin | -0.19 | -0.80 | -0.25 | 1.00 | 1150.44 |  |  |  |  |  |  |  |  |  |  |  |  |  |  |  |  |  |  |
| [PTQNKIFTTI](https://webs.iiitd.edu.in/raghava/toxinpred/pepsearch_S.php?seq=PTQNKIFTTI&thval=0.0) | -1.23 | Non-Toxin | -0.10 | -0.28 | -0.39 | 1.00 | 1162.50 |  |  |  |  |  |  |  |  |  |  |  |  |  |  |  |  |  |  |
| [TQNKIFTTIE](https://webs.iiitd.edu.in/raghava/toxinpred/pepsearch_S.php?seq=TQNKIFTTIE&thval=0.0) | -1.10 | Non-Toxin | -0.15 | -0.47 | -0.09 | 0.00 | 1194.50 |  |  |  |  |  |  |  |  |  |  |  |  |  |  |  |  |  |  |
| [QNKIFTTIEA](https://webs.iiitd.edu.in/raghava/toxinpred/pepsearch_S.php?seq=QNKIFTTIEA&thval=0.0) | -1.02 | Non-Toxin | -0.11 | -0.22 | -0.10 | 0.00 | 1164.47 |  |  |  |  |  |  |  |  |  |  |  |  |  |  |  |  |  |  |
| [NKIFTTIEAG](https://webs.iiitd.edu.in/raghava/toxinpred/pepsearch_S.php?seq=NKIFTTIEAG&thval=0.0) | -0.93 | Non-Toxin | -0.02 | 0.09 | -0.12 | 0.00 | 1093.39 |  |  |  |  |  |  |  |  |  |  |  |  |  |  |  |  |  |  |
| [KIFTTIEAGE](https://webs.iiitd.edu.in/raghava/toxinpred/pepsearch_S.php?seq=KIFTTIEAGE&thval=0.0) | -1.17 | Non-Toxin | -0.02 | 0.09 | 0.16 | -1.00 | 1108.40 |  |  |  |  |  |  |  |  |  |  |  |  |  |  |  |  |  |  |
| [IFTTIEAGEK](https://webs.iiitd.edu.in/raghava/toxinpred/pepsearch_S.php?seq=IFTTIEAGEK&thval=0.0) | -1.21 | Non-Toxin | -0.02 | 0.09 | 0.16 | -1.00 | 1108.40 |  |  |  |  |  |  |  |  |  |  |  |  |  |  |  |  |  |  |
| [FTTIEAGEKN](https://webs.iiitd.edu.in/raghava/toxinpred/pepsearch_S.php?seq=FTTIEAGEKN&thval=0.0) | -1.07 | Non-Toxin | -0.16 | -0.71 | 0.36 | -1.00 | 1109.34 |  |  |  |  |  |  |  |  |  |  |  |  |  |  |  |  |  |  |
| [TTIEAGEKNT](https://webs.iiitd.edu.in/raghava/toxinpred/pepsearch_S.php?seq=TTIEAGEKNT&thval=0.0) | -0.99 | Non-Toxin | -0.24 | -1.06 | 0.57 | -1.00 | 1063.27 |  |  |  |  |  |  |  |  |  |  |  |  |  |  |  |  |  |  |
| [TIEAGEKNTY](https://webs.iiitd.edu.in/raghava/toxinpred/pepsearch_S.php?seq=TIEAGEKNTY&thval=0.0) | -1.02 | Non-Toxin | -0.22 | -1.12 | 0.38 | -1.00 | 1125.34 |  |  |  |  |  |  |  |  |  |  |  |  |  |  |  |  |  |  |
| [IEAGEKNTYK](https://webs.iiitd.edu.in/raghava/toxinpred/pepsearch_S.php?seq=IEAGEKNTYK&thval=0.0) | -0.81 | Non-Toxin | -0.31 | -1.44 | 0.72 | 0.00 | 1152.41 |  |  |  |  |  |  |  |  |  |  |  |  |  |  |  |  |  |  |
| [EAGEKNTYKS](https://webs.iiitd.edu.in/raghava/toxinpred/pepsearch_S.php?seq=EAGEKNTYKS&thval=0.0) | -0.93 | Non-Toxin | -0.41 | -1.97 | 0.93 | 0.00 | 1126.32 |  |  |  |  |  |  |  |  |  |  |  |  |  |  |  |  |  |  |
| [AGEKNTYKSI](https://webs.iiitd.edu.in/raghava/toxinpred/pepsearch_S.php?seq=AGEKNTYKSI&thval=0.0) | -0.97 | Non-Toxin | -0.27 | -1.17 | 0.45 | 1.00 | 1110.37 |  |  |  |  |  |  |  |  |  |  |  |  |  |  |  |  |  |  |
| [GEKNTYKSIL](https://webs.iiitd.edu.in/raghava/toxinpred/pepsearch_S.php?seq=GEKNTYKSIL&thval=0.0) | -0.99 | Non-Toxin | -0.25 | -0.97 | 0.32 | 1.00 | 1152.46 |  |  |  |  |  |  |  |  |  |  |  |  |  |  |  |  |  |  |
| [EKNTYKSILG](https://webs.iiitd.edu.in/raghava/toxinpred/pepsearch_S.php?seq=EKNTYKSILG&thval=0.0) | -0.85 | Non-Toxin | -0.25 | -0.97 | 0.32 | 1.00 | 1152.46 |  |  |  |  |  |  |  |  |  |  |  |  |  |  |  |  |  |  |
| [KNTYKSILGI](https://webs.iiitd.edu.in/raghava/toxinpred/pepsearch_S.php?seq=KNTYKSILGI&thval=0.0) | -0.93 | Non-Toxin | -0.11 | -0.17 | -0.16 | 2.00 | 1136.51 |  |  |  |  |  |  |  |  |  |  |  |  |  |  |  |  |  |  |
| [NTYKSILGID](https://webs.iiitd.edu.in/raghava/toxinpred/pepsearch_S.php?seq=NTYKSILGID&thval=0.0) | -0.89 | Non-Toxin | -0.07 | -0.13 | -0.16 | 0.00 | 1123.42 |  |  |  |  |  |  |  |  |  |  |  |  |  |  |  |  |  |  |
| [TYKSILGIDL](https://webs.iiitd.edu.in/raghava/toxinpred/pepsearch_S.php?seq=TYKSILGIDL&thval=0.0) | -0.97 | Non-Toxin | 0.04 | 0.60 | -0.36 | 0.00 | 1122.48 |  |  |  |  |  |  |  |  |  |  |  |  |  |  |  |  |  |  |
| [YKSILGIDLA](https://webs.iiitd.edu.in/raghava/toxinpred/pepsearch_S.php?seq=YKSILGIDLA&thval=0.0) | -1.09 | Non-Toxin | 0.09 | 0.85 | -0.37 | 0.00 | 1092.45 |  |  |  |  |  |  |  |  |  |  |  |  |  |  |  |  |  |  |
| [KSILGIDLAQ](https://webs.iiitd.edu.in/raghava/toxinpred/pepsearch_S.php?seq=KSILGIDLAQ&thval=0.0) | -1.09 | Non-Toxin | 0.02 | 0.63 | -0.12 | 0.00 | 1057.41 |  |  |  |  |  |  |  |  |  |  |  |  |  |  |  |  |  |  |
| [SILGIDLAQD](https://webs.iiitd.edu.in/raghava/toxinpred/pepsearch_S.php?seq=SILGIDLAQD&thval=0.0) | -1.29 | Non-Toxin | 0.05 | 0.67 | -0.12 | -2.00 | 1044.32 |  |  |  |  |  |  |  |  |  |  |  |  |  |  |  |  |  |  |
| [ILGIDLAQDG](https://webs.iiitd.edu.in/raghava/toxinpred/pepsearch_S.php?seq=ILGIDLAQDG&thval=0.0) | -1.07 | Non-Toxin | 0.10 | 0.71 | -0.15 | -2.00 | 1014.30 |  |  |  |  |  |  |  |  |  |  |  |  |  |  |  |  |  |  |
| [LGIDLAQDGY](https://webs.iiitd.edu.in/raghava/toxinpred/pepsearch_S.php?seq=LGIDLAQDGY&thval=0.0) | -0.88 | Non-Toxin | 0.03 | 0.13 | -0.20 | -2.00 | 1064.31 |  |  |  |  |  |  |  |  |  |  |  |  |  |  |  |  |  |  |
| [GIDLAQDGYY](https://webs.iiitd.edu.in/raghava/toxinpred/pepsearch_S.php?seq=GIDLAQDGYY&thval=0.0) | -0.87 | Non-Toxin | -0.03 | -0.38 | -0.25 | -2.00 | 1114.32 |  |  |  |  |  |  |  |  |  |  |  |  |  |  |  |  |  |  |
| [IDLAQDGYYA](https://webs.iiitd.edu.in/raghava/toxinpred/pepsearch_S.php?seq=IDLAQDGYYA&thval=0.0) | -0.86 | Non-Toxin | -0.02 | -0.16 | -0.30 | -2.00 | 1128.34 |  |  |  |  |  |  |  |  |  |  |  |  |  |  |  |  |  |  |
| [DLAQDGYYAQ](https://webs.iiitd.edu.in/raghava/toxinpred/pepsearch_S.php?seq=DLAQDGYYAQ&thval=0.0) | -0.80 | Non-Toxin | -0.16 | -0.96 | -0.10 | -2.00 | 1143.31 |  |  |  |  |  |  |  |  |  |  |  |  |  |  |  |  |  |  |
| [LAQDGYYAQI](https://webs.iiitd.edu.in/raghava/toxinpred/pepsearch_S.php?seq=LAQDGYYAQI&thval=0.0) | -0.79 | Non-Toxin | -0.01 | -0.16 | -0.58 | -1.00 | 1141.39 |  |  |  |  |  |  |  |  |  |  |  |  |  |  |  |  |  |  |
| [AQDGYYAQIR](https://webs.iiitd.edu.in/raghava/toxinpred/pepsearch_S.php?seq=AQDGYYAQIR&thval=0.0) | -0.56 | Non-Toxin | -0.24 | -0.99 | -0.10 | 0.00 | 1184.41 |  |  |  |  |  |  |  |  |  |  |  |  |  |  |  |  |  |  |
| [QDGYYAQIRG](https://webs.iiitd.edu.in/raghava/toxinpred/pepsearch_S.php?seq=QDGYYAQIRG&thval=0.0) | -0.61 | Non-Toxin | -0.25 | -1.21 | -0.05 | 0.00 | 1170.39 |  |  |  |  |  |  |  |  |  |  |  |  |  |  |  |  |  |  |
| [DGYYAQIRGQ](https://webs.iiitd.edu.in/raghava/toxinpred/pepsearch_S.php?seq=DGYYAQIRGQ&thval=0.0) | -0.45 | Non-Toxin | -0.25 | -1.21 | -0.05 | 0.00 | 1170.39 |  |  |  |  |  |  |  |  |  |  |  |  |  |  |  |  |  |  |
| [GYYAQIRGQR](https://webs.iiitd.edu.in/raghava/toxinpred/pepsearch_S.php?seq=GYYAQIRGQR&thval=0.0) | -0.53 | Non-Toxin | -0.36 | -1.31 | -0.05 | 2.00 | 1211.49 |  |  |  |  |  |  |  |  |  |  |  |  |  |  |  |  |  |  |
| [YYAQIRGQRF](https://webs.iiitd.edu.in/raghava/toxinpred/pepsearch_S.php?seq=YYAQIRGQRF&thval=0.0) | -0.70 | Non-Toxin | -0.31 | -0.99 | -0.30 | 2.00 | 1301.61 |  |  |  |  |  |  |  |  |  |  |  |  |  |  |  |  |  |  |
| [YAQIRGQRFE](https://webs.iiitd.edu.in/raghava/toxinpred/pepsearch_S.php?seq=YAQIRGQRFE&thval=0.0) | -0.79 | Non-Toxin | -0.38 | -1.21 | 0.23 | 1.00 | 1267.55 |  |  |  |  |  |  |  |  |  |  |  |  |  |  |  |  |  |  |
| [AQIRGQRFET](https://webs.iiitd.edu.in/raghava/toxinpred/pepsearch_S.php?seq=AQIRGQRFET&thval=0.0) | -0.80 | Non-Toxin | -0.40 | -1.15 | 0.42 | 1.00 | 1205.48 |  |  |  |  |  |  |  |  |  |  |  |  |  |  |  |  |  |  |
| [QIRGQRFETD](https://webs.iiitd.edu.in/raghava/toxinpred/pepsearch_S.php?seq=QIRGQRFETD&thval=0.0) | -0.87 | Non-Toxin | -0.49 | -1.68 | 0.77 | 0.00 | 1249.49 |  |  |  |  |  |  |  |  |  |  |  |  |  |  |  |  |  |  |
| [IRGQRFETDG](https://webs.iiitd.edu.in/raghava/toxinpred/pepsearch_S.php?seq=IRGQRFETDG&thval=0.0) | -0.90 | Non-Toxin | -0.41 | -1.37 | 0.75 | 0.00 | 1178.41 |  |  |  |  |  |  |  |  |  |  |  |  |  |  |  |  |  |  |
| [RGQRFETDGD](https://webs.iiitd.edu.in/raghava/toxinpred/pepsearch_S.php?seq=RGQRFETDGD&thval=0.0) | -1.02 | Non-Toxin | -0.55 | -2.17 | 1.23 | -1.00 | 1180.33 |  |  |  |  |  |  |  |  |  |  |  |  |  |  |  |  |  |  |
| [GQRFETDGDQ](https://webs.iiitd.edu.in/raghava/toxinpred/pepsearch_S.php?seq=GQRFETDGDQ&thval=0.0) | -0.96 | Non-Toxin | -0.44 | -2.07 | 0.95 | -2.00 | 1152.28 |  |  |  |  |  |  |  |  |  |  |  |  |  |  |  |  |  |  |
| [QRFETDGDQI](https://webs.iiitd.edu.in/raghava/toxinpred/pepsearch_S.php?seq=QRFETDGDQI&thval=0.0) | -0.85 | Non-Toxin | -0.39 | -1.58 | 0.77 | -2.00 | 1208.39 |  |  |  |  |  |  |  |  |  |  |  |  |  |  |  |  |  |  |
| [RFETDGDQII](https://webs.iiitd.edu.in/raghava/toxinpred/pepsearch_S.php?seq=RFETDGDQII&thval=0.0) | -0.69 | Non-Toxin | -0.25 | -0.78 | 0.57 | -2.00 | 1193.42 |  |  |  |  |  |  |  |  |  |  |  |  |  |  |  |  |  |  |
| [FETDGDQIIS](https://webs.iiitd.edu.in/raghava/toxinpred/pepsearch_S.php?seq=FETDGDQIIS&thval=0.0) | -0.78 | Non-Toxin | -0.10 | -0.41 | 0.30 | -3.00 | 1124.31 |  |  |  |  |  |  |  |  |  |  |  |  |  |  |  |  |  |  |
| [ETDGDQIISN](https://webs.iiitd.edu.in/raghava/toxinpred/pepsearch_S.php?seq=ETDGDQIISN&thval=0.0) | -0.83 | Non-Toxin | -0.22 | -1.04 | 0.57 | -3.00 | 1091.24 |  |  |  |  |  |  |  |  |  |  |  |  |  |  |  |  |  |  |
| [TDGDQIISND](https://webs.iiitd.edu.in/raghava/toxinpred/pepsearch_S.php?seq=TDGDQIISND&thval=0.0) | -0.68 | Non-Toxin | -0.23 | -1.04 | 0.57 | -3.00 | 1077.21 |  |  |  |  |  |  |  |  |  |  |  |  |  |  |  |  |  |  |
| [DGDQIISNDD](https://webs.iiitd.edu.in/raghava/toxinpred/pepsearch_S.php?seq=DGDQIISNDD&thval=0.0) | -0.63 | Non-Toxin | -0.28 | -1.32 | 0.91 | -4.00 | 1091.19 |  |  |  |  |  |  |  |  |  |  |  |  |  |  |  |  |  |  |
| [GDQIISNDDR](https://webs.iiitd.edu.in/raghava/toxinpred/pepsearch_S.php?seq=GDQIISNDDR&thval=0.0) | -0.79 | Non-Toxin | -0.39 | -1.42 | 0.91 | -2.00 | 1132.29 |  |  |  |  |  |  |  |  |  |  |  |  |  |  |  |  |  |  |
| [DQIISNDDRK](https://webs.iiitd.edu.in/raghava/toxinpred/pepsearch_S.php?seq=DQIISNDDRK&thval=0.0) | -0.74 | Non-Toxin | -0.52 | -1.77 | 1.21 | -1.00 | 1203.41 |  |  |  |  |  |  |  |  |  |  |  |  |  |  |  |  |  |  |
| [QIISNDDRKA](https://webs.iiitd.edu.in/raghava/toxinpred/pepsearch_S.php?seq=QIISNDDRKA&thval=0.0) | -0.85 | Non-Toxin | -0.42 | -1.24 | 0.86 | 0.00 | 1159.40 |  |  |  |  |  |  |  |  |  |  |  |  |  |  |  |  |  |  |
| [IISNDDRKAG](https://webs.iiitd.edu.in/raghava/toxinpred/pepsearch_S.php?seq=IISNDDRKAG&thval=0.0) | -0.89 | Non-Toxin | -0.33 | -0.93 | 0.84 | 0.00 | 1088.32 |  |  |  |  |  |  |  |  |  |  |  |  |  |  |  |  |  |  |
| [ISNDDRKAGF](https://webs.iiitd.edu.in/raghava/toxinpred/pepsearch_S.php?seq=ISNDDRKAGF&thval=0.0) | -1.05 | Non-Toxin | -0.35 | -1.10 | 0.77 | 0.00 | 1122.33 |  |  |  |  |  |  |  |  |  |  |  |  |  |  |  |  |  |  |
| [SNDDRKAGFD](https://webs.iiitd.edu.in/raghava/toxinpred/pepsearch_S.php?seq=SNDDRKAGFD&thval=0.0) | -1.02 | Non-Toxin | -0.49 | -1.90 | 1.25 | -1.00 | 1124.25 |  |  |  |  |  |  |  |  |  |  |  |  |  |  |  |  |  |  |
| [NDDRKAGFDQ](https://webs.iiitd.edu.in/raghava/toxinpred/pepsearch_S.php?seq=NDDRKAGFDQ&thval=0.0) | -0.91 | Non-Toxin | -0.53 | -2.17 | 1.24 | -1.00 | 1165.31 |  |  |  |  |  |  |  |  |  |  |  |  |  |  |  |  |  |  |
| [DDRKAGFDQK](https://webs.iiitd.edu.in/raghava/toxinpred/pepsearch_S.php?seq=DDRKAGFDQK&thval=0.0) | -0.94 | Non-Toxin | -0.58 | -2.21 | 1.52 | 0.00 | 1179.38 |  |  |  |  |  |  |  |  |  |  |  |  |  |  |  |  |  |  |
| [DRKAGFDQKG](https://webs.iiitd.edu.in/raghava/toxinpred/pepsearch_S.php?seq=DRKAGFDQKG&thval=0.0) | -0.88 | Non-Toxin | -0.49 | -1.90 | 1.22 | 1.00 | 1121.35 |  |  |  |  |  |  |  |  |  |  |  |  |  |  |  |  |  |  |
| [RKAGFDQKGY](https://webs.iiitd.edu.in/raghava/toxinpred/pepsearch_S.php?seq=RKAGFDQKGY&thval=0.0) | -0.66 | Non-Toxin | -0.42 | -1.68 | 0.69 | 2.00 | 1169.44 |  |  |  |  |  |  |  |  |  |  |  |  |  |  |  |  |  |  |
| [KAGFDQKGYS](https://webs.iiitd.edu.in/raghava/toxinpred/pepsearch_S.php?seq=KAGFDQKGYS&thval=0.0) | -0.68 | Non-Toxin | -0.27 | -1.31 | 0.42 | 1.00 | 1100.33 |  |  |  |  |  |  |  |  |  |  |  |  |  |  |  |  |  |  |
| [AGFDQKGYSA](https://webs.iiitd.edu.in/raghava/toxinpred/pepsearch_S.php?seq=AGFDQKGYSA&thval=0.0) | -0.68 | Non-Toxin | -0.13 | -0.74 | 0.07 | 0.00 | 1043.23 |  |  |  |  |  |  |  |  |  |  |  |  |  |  |  |  |  |  |
| [GFDQKGYSAK](https://webs.iiitd.edu.in/raghava/toxinpred/pepsearch_S.php?seq=GFDQKGYSAK&thval=0.0) | -0.36 | Non-Toxin | -0.27 | -1.31 | 0.42 | 1.00 | 1100.33 |  |  |  |  |  |  |  |  |  |  |  |  |  |  |  |  |  |  |
| [FDQKGYSAKV](https://webs.iiitd.edu.in/raghava/toxinpred/pepsearch_S.php?seq=FDQKGYSAKV&thval=0.0) | -0.28 | Non-Toxin | -0.23 | -0.85 | 0.27 | 1.00 | 1142.41 |  |  |  |  |  |  |  |  |  |  |  |  |  |  |  |  |  |  |
| [DQKGYSAKVG](https://webs.iiitd.edu.in/raghava/toxinpred/pepsearch_S.php?seq=DQKGYSAKVG&thval=0.0) | -0.33 | Non-Toxin | -0.27 | -1.17 | 0.52 | 1.00 | 1052.29 |  |  |  |  |  |  |  |  |  |  |  |  |  |  |  |  |  |  |
| [QKGYSAKVGV](https://webs.iiitd.edu.in/raghava/toxinpred/pepsearch_S.php?seq=QKGYSAKVGV&thval=0.0) | -0.62 | Non-Toxin | -0.15 | -0.40 | 0.07 | 2.00 | 1036.34 |  |  |  |  |  |  |  |  |  |  |  |  |  |  |  |  |  |  |
| [KGYSAKVGVD](https://webs.iiitd.edu.in/raghava/toxinpred/pepsearch_S.php?seq=KGYSAKVGVD&thval=0.0) | -0.69 | Non-Toxin | -0.15 | -0.40 | 0.35 | 1.00 | 1023.29 |  |  |  |  |  |  |  |  |  |  |  |  |  |  |  |  |  |  |
| [GYSAKVGVDK](https://webs.iiitd.edu.in/raghava/toxinpred/pepsearch_S.php?seq=GYSAKVGVDK&thval=0.0) | -0.81 | Non-Toxin | -0.15 | -0.40 | 0.35 | 1.00 | 1023.29 |  |  |  |  |  |  |  |  |  |  |  |  |  |  |  |  |  |  |
| [YSAKVGVDKE](https://webs.iiitd.edu.in/raghava/toxinpred/pepsearch_S.php?seq=YSAKVGVDKE&thval=0.0) | -0.91 | Non-Toxin | -0.23 | -0.71 | 0.65 | 0.00 | 1095.35 |  |  |  |  |  |  |  |  |  |  |  |  |  |  |  |  |  |  |
| [SAKVGVDKEQ](https://webs.iiitd.edu.in/raghava/toxinpred/pepsearch_S.php?seq=SAKVGVDKEQ&thval=0.0) | -1.00 | Non-Toxin | -0.30 | -0.93 | 0.90 | 0.00 | 1060.31 |  |  |  |  |  |  |  |  |  |  |  |  |  |  |  |  |  |  |
| [AKVGVDKEQY](https://webs.iiitd.edu.in/raghava/toxinpred/pepsearch_S.php?seq=AKVGVDKEQY&thval=0.0) | -0.93 | Non-Toxin | -0.27 | -0.98 | 0.64 | 0.00 | 1136.41 |  |  |  |  |  |  |  |  |  |  |  |  |  |  |  |  |  |  |
| [KVGVDKEQYA](https://webs.iiitd.edu.in/raghava/toxinpred/pepsearch_S.php?seq=KVGVDKEQYA&thval=0.0) | -1.18 | Non-Toxin | -0.27 | -0.98 | 0.64 | 0.00 | 1136.41 |  |  |  |  |  |  |  |  |  |  |  |  |  |  |  |  |  |  |
| [VGVDKEQYAL](https://webs.iiitd.edu.in/raghava/toxinpred/pepsearch_S.php?seq=VGVDKEQYAL&thval=0.0) | -0.99 | Non-Toxin | -0.11 | -0.21 | 0.16 | -1.00 | 1121.40 |  |  |  |  |  |  |  |  |  |  |  |  |  |  |  |  |  |  |
| [GVDKEQYALS](https://webs.iiitd.edu.in/raghava/toxinpred/pepsearch_S.php?seq=GVDKEQYALS&thval=0.0) | -1.00 | Non-Toxin | -0.19 | -0.71 | 0.34 | -1.00 | 1109.34 |  |  |  |  |  |  |  |  |  |  |  |  |  |  |  |  |  |  |
| [VDKEQYALSA](https://webs.iiitd.edu.in/raghava/toxinpred/pepsearch_S.php?seq=VDKEQYALSA&thval=0.0) | -0.78 | Non-Toxin | -0.18 | -0.49 | 0.29 | -1.00 | 1123.36 |  |  |  |  |  |  |  |  |  |  |  |  |  |  |  |  |  |  |
| [DKEQYALSAE](https://webs.iiitd.edu.in/raghava/toxinpred/pepsearch_S.php?seq=DKEQYALSAE&thval=0.0) | -0.80 | Non-Toxin | -0.30 | -1.26 | 0.74 | -2.00 | 1153.34 |  |  |  |  |  |  |  |  |  |  |  |  |  |  |  |  |  |  |
| [KEQYALSAEI](https://webs.iiitd.edu.in/raghava/toxinpred/pepsearch_S.php?seq=KEQYALSAEI&thval=0.0) | -0.87 | Non-Toxin | -0.15 | -0.46 | 0.26 | -1.00 | 1151.42 |  |  |  |  |  |  |  |  |  |  |  |  |  |  |  |  |  |  |
| [EQYALSAEIK](https://webs.iiitd.edu.in/raghava/toxinpred/pepsearch_S.php?seq=EQYALSAEIK&thval=0.0) | -0.92 | Non-Toxin | -0.15 | -0.46 | 0.26 | -1.00 | 1151.42 |  |  |  |  |  |  |  |  |  |  |  |  |  |  |  |  |  |  |
| [QYALSAEIKE](https://webs.iiitd.edu.in/raghava/toxinpred/pepsearch_S.php?seq=QYALSAEIKE&thval=0.0) | -0.82 | Non-Toxin | -0.15 | -0.46 | 0.26 | -1.00 | 1151.42 |  |  |  |  |  |  |  |  |  |  |  |  |  |  |  |  |  |  |
| [YALSAEIKEN](https://webs.iiitd.edu.in/raghava/toxinpred/pepsearch_S.php?seq=YALSAEIKEN&thval=0.0) | -0.85 | Non-Toxin | -0.15 | -0.46 | 0.26 | -1.00 | 1137.39 |  |  |  |  |  |  |  |  |  |  |  |  |  |  |  |  |  |  |
| [ALSAEIKENK](https://webs.iiitd.edu.in/raghava/toxinpred/pepsearch_S.php?seq=ALSAEIKENK&thval=0.0) | -0.56 | Non-Toxin | -0.26 | -0.72 | 0.79 | 0.00 | 1102.39 |  |  |  |  |  |  |  |  |  |  |  |  |  |  |  |  |  |  |
| [LSAEIKENKG](https://webs.iiitd.edu.in/raghava/toxinpred/pepsearch_S.php?seq=LSAEIKENKG&thval=0.0) | -0.60 | Non-Toxin | -0.27 | -0.94 | 0.84 | 0.00 | 1088.37 |  |  |  |  |  |  |  |  |  |  |  |  |  |  |  |  |  |  |
| [SAEIKENKGT](https://webs.iiitd.edu.in/raghava/toxinpred/pepsearch_S.php?seq=SAEIKENKGT&thval=0.0) | -0.61 | Non-Toxin | -0.34 | -1.39 | 0.98 | 0.00 | 1076.31 |  |  |  |  |  |  |  |  |  |  |  |  |  |  |  |  |  |  |
| [AEIKENKGTG](https://webs.iiitd.edu.in/raghava/toxinpred/pepsearch_S.php?seq=AEIKENKGTG&thval=0.0) | -0.55 | Non-Toxin | -0.30 | -1.35 | 0.95 | 0.00 | 1046.29 |  |  |  |  |  |  |  |  |  |  |  |  |  |  |  |  |  |  |
| [EIKENKGTGD](https://webs.iiitd.edu.in/raghava/toxinpred/pepsearch_S.php?seq=EIKENKGTGD&thval=0.0) | -0.52 | Non-Toxin | -0.39 | -1.88 | 1.30 | -1.00 | 1090.30 |  |  |  |  |  |  |  |  |  |  |  |  |  |  |  |  |  |  |
| [IKENKGTGDF](https://webs.iiitd.edu.in/raghava/toxinpred/pepsearch_S.php?seq=IKENKGTGDF&thval=0.0) | -0.65 | Non-Toxin | -0.27 | -1.25 | 0.75 | 0.00 | 1108.36 |  |  |  |  |  |  |  |  |  |  |  |  |  |  |  |  |  |  |
| [KENKGTGDFF](https://webs.iiitd.edu.in/raghava/toxinpred/pepsearch_S.php?seq=KENKGTGDFF&thval=0.0) | -0.52 | Non-Toxin | -0.28 | -1.42 | 0.68 | 0.00 | 1142.37 |  |  |  |  |  |  |  |  |  |  |  |  |  |  |  |  |  |  |

| **Peptides Scanned from Original Protein** | | | | |  | | | | | | | | | | | | | | | | | | | | |
| --- | --- | --- | --- | --- | --- | --- | --- | --- | --- | --- | --- | --- | --- | --- | --- | --- | --- | --- | --- | --- | --- | --- | --- | --- | --- |
| [**Peptide Sequence**](https://webs.iiitd.edu.in/raghava/toxinpred/prot_submitfreq_S.php?ran=8943) | [**SVM score**](https://webs.iiitd.edu.in/raghava/toxinpred/prot_submitfreq_S.php?ran=8943) | [**Prediction**](https://webs.iiitd.edu.in/raghava/toxinpred/prot_submitfreq_S.php?ran=8943) | [**Hydrophobicity**](https://webs.iiitd.edu.in/raghava/toxinpred/prot_submitfreq_S.php?ran=8943) | [**Hydropathicity**](https://webs.iiitd.edu.in/raghava/toxinpred/prot_submitfreq_S.php?ran=8943) | [**Hydrophilicity**](https://webs.iiitd.edu.in/raghava/toxinpred/prot_submitfreq_S.php?ran=8943) | [**Charge**](https://webs.iiitd.edu.in/raghava/toxinpred/prot_submitfreq_S.php?ran=8943) | [**Mol wt**](https://webs.iiitd.edu.in/raghava/toxinpred/prot_submitfreq_S.php?ran=8943) |  |  |  |  |  |  |  |  |  |  |  |  |  |  |  |  |  |  |
| [MNIGWDNGVY](https://webs.iiitd.edu.in/raghava/toxinpred/pepsearch_S.php?seq=MNIGWDNGVY&thval=0.0) | -1.04 | Non-Toxin | 0.02 | -0.29 | -0.69 | -1.00 | 1168.44 |  |  |  |  |  |  |  |  |  |  |  |  |  |  |  |  |  |  |
| [NIGWDNGVYG](https://webs.iiitd.edu.in/raghava/toxinpred/pepsearch_S.php?seq=NIGWDNGVYG&thval=0.0) | -0.92 | Non-Toxin | 0.01 | -0.52 | -0.56 | -1.00 | 1094.30 |  |  |  |  |  |  |  |  |  |  |  |  |  |  |  |  |  |  |
| [IGWDNGVYGF](https://webs.iiitd.edu.in/raghava/toxinpred/pepsearch_S.php?seq=IGWDNGVYGF&thval=0.0) | -0.86 | Non-Toxin | 0.14 | 0.11 | -0.83 | -1.00 | 1127.37 |  |  |  |  |  |  |  |  |  |  |  |  |  |  |  |  |  |  |
| [GWDNGVYGFN](https://webs.iiitd.edu.in/raghava/toxinpred/pepsearch_S.php?seq=GWDNGVYGFN&thval=0.0) | -0.85 | Non-Toxin | 0.00 | -0.69 | -0.63 | -1.00 | 1128.31 |  |  |  |  |  |  |  |  |  |  |  |  |  |  |  |  |  |  |
| [WDNGVYGFNT](https://webs.iiitd.edu.in/raghava/toxinpred/pepsearch_S.php?seq=WDNGVYGFNT&thval=0.0) | -0.84 | Non-Toxin | -0.03 | -0.72 | -0.67 | -1.00 | 1172.36 |  |  |  |  |  |  |  |  |  |  |  |  |  |  |  |  |  |  |
| [DNGVYGFNTA](https://webs.iiitd.edu.in/raghava/toxinpred/pepsearch_S.php?seq=DNGVYGFNTA&thval=0.0) | -1.06 | Non-Toxin | -0.04 | -0.45 | -0.38 | -1.00 | 1057.22 |  |  |  |  |  |  |  |  |  |  |  |  |  |  |  |  |  |  |
| [NGVYGFNTAF](https://webs.iiitd.edu.in/raghava/toxinpred/pepsearch_S.php?seq=NGVYGFNTAF&thval=0.0) | -1.17 | Non-Toxin | 0.09 | 0.18 | -0.93 | 0.00 | 1089.31 |  |  |  |  |  |  |  |  |  |  |  |  |  |  |  |  |  |  |
| [GVYGFNTAFV](https://webs.iiitd.edu.in/raghava/toxinpred/pepsearch_S.php?seq=GVYGFNTAFV&thval=0.0) | -1.26 | Non-Toxin | 0.21 | 0.95 | -1.10 | 0.00 | 1074.34 |  |  |  |  |  |  |  |  |  |  |  |  |  |  |  |  |  |  |
| [VYGFNTAFVA](https://webs.iiitd.edu.in/raghava/toxinpred/pepsearch_S.php?seq=VYGFNTAFVA&thval=0.0) | -1.16 | Non-Toxin | 0.22 | 1.17 | -1.15 | 0.00 | 1088.36 |  |  |  |  |  |  |  |  |  |  |  |  |  |  |  |  |  |  |
| [YGFNTAFVAK](https://webs.iiitd.edu.in/raghava/toxinpred/pepsearch_S.php?seq=YGFNTAFVAK&thval=0.0) | -0.76 | Non-Toxin | 0.05 | 0.36 | -0.70 | 1.00 | 1117.40 |  |  |  |  |  |  |  |  |  |  |  |  |  |  |  |  |  |  |
| [GFNTAFVAKG](https://webs.iiitd.edu.in/raghava/toxinpred/pepsearch_S.php?seq=GFNTAFVAKG&thval=0.0) | -0.58 | Non-Toxin | 0.07 | 0.45 | -0.47 | 1.00 | 1011.28 |  |  |  |  |  |  |  |  |  |  |  |  |  |  |  |  |  |  |
| [FNTAFVAKGK](https://webs.iiitd.edu.in/raghava/toxinpred/pepsearch_S.php?seq=FNTAFVAKGK&thval=0.0) | -0.48 | Non-Toxin | -0.06 | 0.10 | -0.17 | 2.00 | 1082.40 |  |  |  |  |  |  |  |  |  |  |  |  |  |  |  |  |  |  |
| [NTAFVAKGKA](https://webs.iiitd.edu.in/raghava/toxinpred/pepsearch_S.php?seq=NTAFVAKGKA&thval=0.0) | -0.38 | Non-Toxin | -0.10 | -0.00 | 0.03 | 2.00 | 1006.30 |  |  |  |  |  |  |  |  |  |  |  |  |  |  |  |  |  |  |
| [TAFVAKGKAK](https://webs.iiitd.edu.in/raghava/toxinpred/pepsearch_S.php?seq=TAFVAKGKAK&thval=0.0) | 0.06 | Toxin | -0.14 | -0.04 | 0.31 | 3.00 | 1020.37 |  |  |  |  |  |  |  |  |  |  |  |  |  |  |  |  |  |  |
| [AFVAKGKAKD](https://webs.iiitd.edu.in/raghava/toxinpred/pepsearch_S.php?seq=AFVAKGKAKD&thval=0.0) | -0.00 | Non-Toxin | -0.20 | -0.32 | 0.65 | 2.00 | 1034.35 |  |  |  |  |  |  |  |  |  |  |  |  |  |  |  |  |  |  |
| [FVAKGKAKDI](https://webs.iiitd.edu.in/raghava/toxinpred/pepsearch_S.php?seq=FVAKGKAKDI&thval=0.0) | -0.00 | Non-Toxin | -0.15 | -0.05 | 0.52 | 2.00 | 1076.44 |  |  |  |  |  |  |  |  |  |  |  |  |  |  |  |  |  |  |
| [VAKGKAKDIQ](https://webs.iiitd.edu.in/raghava/toxinpred/pepsearch_S.php?seq=VAKGKAKDIQ&thval=0.0) | -0.09 | Non-Toxin | -0.28 | -0.68 | 0.79 | 2.00 | 1057.40 |  |  |  |  |  |  |  |  |  |  |  |  |  |  |  |  |  |  |
| [AKGKAKDIQD](https://webs.iiitd.edu.in/raghava/toxinpred/pepsearch_S.php?seq=AKGKAKDIQD&thval=0.0) | -0.28 | Non-Toxin | -0.40 | -1.45 | 1.24 | 1.00 | 1073.35 |  |  |  |  |  |  |  |  |  |  |  |  |  |  |  |  |  |  |
| [KGKAKDIQDV](https://webs.iiitd.edu.in/raghava/toxinpred/pepsearch_S.php?seq=KGKAKDIQDV&thval=0.0) | -0.78 | Non-Toxin | -0.38 | -1.21 | 1.14 | 1.00 | 1101.41 |  |  |  |  |  |  |  |  |  |  |  |  |  |  |  |  |  |  |
| [GKAKDIQDVP](https://webs.iiitd.edu.in/raghava/toxinpred/pepsearch_S.php?seq=GKAKDIQDVP&thval=0.0) | -1.01 | Non-Toxin | -0.27 | -0.98 | 0.84 | 0.00 | 1070.35 |  |  |  |  |  |  |  |  |  |  |  |  |  |  |  |  |  |  |
| [KAKDIQDVPG](https://webs.iiitd.edu.in/raghava/toxinpred/pepsearch_S.php?seq=KAKDIQDVPG&thval=0.0) | -1.11 | Non-Toxin | -0.27 | -0.98 | 0.84 | 0.00 | 1070.35 |  |  |  |  |  |  |  |  |  |  |  |  |  |  |  |  |  |  |
| [AKDIQDVPGY](https://webs.iiitd.edu.in/raghava/toxinpred/pepsearch_S.php?seq=AKDIQDVPGY&thval=0.0) | -1.07 | Non-Toxin | -0.16 | -0.72 | 0.31 | -1.00 | 1105.35 |  |  |  |  |  |  |  |  |  |  |  |  |  |  |  |  |  |  |
| [KDIQDVPGYT](https://webs.iiitd.edu.in/raghava/toxinpred/pepsearch_S.php?seq=KDIQDVPGYT&thval=0.0) | -1.31 | Non-Toxin | -0.20 | -0.97 | 0.32 | -1.00 | 1135.38 |  |  |  |  |  |  |  |  |  |  |  |  |  |  |  |  |  |  |
| [DIQDVPGYTT](https://webs.iiitd.edu.in/raghava/toxinpred/pepsearch_S.php?seq=DIQDVPGYTT&thval=0.0) | -1.16 | Non-Toxin | -0.11 | -0.65 | -0.02 | -2.00 | 1108.31 |  |  |  |  |  |  |  |  |  |  |  |  |  |  |  |  |  |  |
| [IQDVPGYTTL](https://webs.iiitd.edu.in/raghava/toxinpred/pepsearch_S.php?seq=IQDVPGYTTL&thval=0.0) | -1.26 | Non-Toxin | 0.01 | 0.08 | -0.50 | -1.00 | 1106.39 |  |  |  |  |  |  |  |  |  |  |  |  |  |  |  |  |  |  |
| [QDVPGYTTLD](https://webs.iiitd.edu.in/raghava/toxinpred/pepsearch_S.php?seq=QDVPGYTTLD&thval=0.0) | -1.21 | Non-Toxin | -0.13 | -0.72 | -0.02 | -2.00 | 1108.31 |  |  |  |  |  |  |  |  |  |  |  |  |  |  |  |  |  |  |
| [DVPGYTTLDF](https://webs.iiitd.edu.in/raghava/toxinpred/pepsearch_S.php?seq=DVPGYTTLDF&thval=0.0) | -1.11 | Non-Toxin | -0.00 | -0.09 | -0.29 | -2.00 | 1127.35 |  |  |  |  |  |  |  |  |  |  |  |  |  |  |  |  |  |  |
| [VPGYTTLDFN](https://webs.iiitd.edu.in/raghava/toxinpred/pepsearch_S.php?seq=VPGYTTLDFN&thval=0.0) | -1.32 | Non-Toxin | 0.01 | -0.09 | -0.57 | -1.00 | 1126.37 |  |  |  |  |  |  |  |  |  |  |  |  |  |  |  |  |  |  |
| [PGYTTLDFNA](https://webs.iiitd.edu.in/raghava/toxinpred/pepsearch_S.php?seq=PGYTTLDFNA&thval=0.0) | -1.08 | Non-Toxin | -0.02 | -0.33 | -0.47 | -1.00 | 1098.31 |  |  |  |  |  |  |  |  |  |  |  |  |  |  |  |  |  |  |
| [GYTTLDFNAY](https://webs.iiitd.edu.in/raghava/toxinpred/pepsearch_S.php?seq=GYTTLDFNAY&thval=0.0) | -1.07 | Non-Toxin | -0.01 | -0.30 | -0.70 | -1.00 | 1164.37 |  |  |  |  |  |  |  |  |  |  |  |  |  |  |  |  |  |  |
| [YTTLDFNAYW](https://webs.iiitd.edu.in/raghava/toxinpred/pepsearch_S.php?seq=YTTLDFNAYW&thval=0.0) | -1.26 | Non-Toxin | 0.01 | -0.35 | -1.04 | -1.00 | 1293.53 |  |  |  |  |  |  |  |  |  |  |  |  |  |  |  |  |  |  |
| [TTLDFNAYWQ](https://webs.iiitd.edu.in/raghava/toxinpred/pepsearch_S.php?seq=TTLDFNAYWQ&thval=0.0) | -1.27 | Non-Toxin | -0.06 | -0.57 | -0.79 | -1.00 | 1258.49 |  |  |  |  |  |  |  |  |  |  |  |  |  |  |  |  |  |  |
| [TLDFNAYWQM](https://webs.iiitd.edu.in/raghava/toxinpred/pepsearch_S.php?seq=TLDFNAYWQM&thval=0.0) | -1.28 | Non-Toxin | -0.02 | -0.31 | -0.88 | -1.00 | 1288.58 |  |  |  |  |  |  |  |  |  |  |  |  |  |  |  |  |  |  |
| [LDFNAYWQMS](https://webs.iiitd.edu.in/raghava/toxinpred/pepsearch_S.php?seq=LDFNAYWQMS&thval=0.0) | -1.26 | Non-Toxin | -0.03 | -0.32 | -0.81 | -1.00 | 1274.55 |  |  |  |  |  |  |  |  |  |  |  |  |  |  |  |  |  |  |
| [DFNAYWQMSP](https://webs.iiitd.edu.in/raghava/toxinpred/pepsearch_S.php?seq=DFNAYWQMSP&thval=0.0) | -1.00 | Non-Toxin | -0.09 | -0.86 | -0.63 | -1.00 | 1258.50 |  |  |  |  |  |  |  |  |  |  |  |  |  |  |  |  |  |  |
| [FNAYWQMSPN](https://webs.iiitd.edu.in/raghava/toxinpred/pepsearch_S.php?seq=FNAYWQMSPN&thval=0.0) | -0.83 | Non-Toxin | -0.08 | -0.86 | -0.91 | 0.00 | 1257.52 |  |  |  |  |  |  |  |  |  |  |  |  |  |  |  |  |  |  |
